# Psychological Well-Being and Treatment Adherence in COPD Patients: A Correspondence Analysis of WHO-5, MARS, and GOLD Severity

**DOI:** 10.3390/healthcare14040514

**Published:** 2026-02-18

**Authors:** Adina Deliu, Luana Alexandrescu, Bogdan Cimpineanu, Oana Cristina Arghir, Sanda Jurja, Ioan Tiberiu Tofolean, Rodica Gabriela Enache, Ioana Gherghisan, Ionela Preotesoiu, Ionut Valentin Stanciu, Andreea Nelson Twakor, Monica Cordos, Alexandra Herlo, Daria Maria Alexandrescu, Doina Ecaterina Tofolean

**Affiliations:** 1 Doctoral School, Faculty of General Medicine, “Ovidius” University, 900470 Constanta, Romania; adina.deliu@365.univ-ovidius.ro (A.D.); oana.arghir@365.univ-ovidius.ro (O.C.A.); sanda.jurja@365.univ-ovidius.ro (S.J.); ioana.husaru@gmail.com (I.G.); ionela.preotesoiu@365.univ-ovidius.ro (I.P.); ionutstvalentin@gmail.com (I.V.S.); doina.tofolean@365.univ-ovidius.ro (D.E.T.); 2 Faculty of General Medicine, “Ovidius” University, 900470 Constanta, Romania; bogdan.cimpineanu@365.univ-ovidius.ro (B.C.); ioan.tofolean@365.univ-ovidius.ro (I.T.T.); 3 Gastroenterology Department, “Sf. Apostol Andrei” Emergency County Hospital, 145 Tomis Blvd., 900591 Constanta, Romania; 4 Department of Internal Medicine, “Sf. Apostol Andrei” Emergency County Hospital, 145 Tomis Blvd., 900591 Constanta, Romania; andreea.purcaru@365.univ-ovidius.ro; 5 Nephrology Department, “Sf. Apostol Andrei” Emergency County Hospital, 145 Tomis Blvd., 900591 Constanta, Romania; 6 Pneumology Department, Pneumo-phthisiology Hospital Palazu Mare, 40 Santinelei Street, 900002 Constanta, Romania; 7 Faculty of Psychology and Educational Sciences, “Ovidius” University, 900470 Constanta, Romania; rodica-gabriela.enache@365.univ-ovidius.ro; 8 Pneumology Department, “Sf. Apostol Andrei” Emergency County Hospital, 145 Tomis Blvd., 900591 Constanta, Romania; 9 Nephrology Department, Clinical Emergency County Hospital Saint John the New in Suceava, 720229 Suceava, Romania; cordos.monica@gmail.com; 10 Department XIII, Discipline of Infectious Diseases, “Victor Babes” University of Medicine and Pharmacy Timisoara, 2 Eftimie Murgu Square, 300041 Timisoara, Romania; alexandra.mocanu@umft.ro; 11 Faculty of Medicine, Titu Maiorescu University, 040051 Bucharest, Romania; alexandrescu_daria@yahoo.com

**Keywords:** chronic obstructive pulmonary disease, COPD severity, anxiety and depression, psychological well-being, WHO-5 well-being index, medication adherence, MARS scale, patient-reported outcomes, GOLD classification, correspondence analysis

## Abstract

**Background:** Chronic obstructive pulmonary disease (COPD) is frequently complicated by psychological distress and suboptimal treatment adherence, both of which adversely affect clinical outcomes. However, the relationship between disease severity, emotional well-being, and adherence behavior remains insufficiently characterized. **Methods:** This multicenter observational study included 285 patients with COPD recruited from two primary care clinics. COPD severity was classified according to GOLD criteria. Psychological well-being was assessed using the World Health Organization–Five Well-Being Index (WHO-5), and treatment adherence was evaluated using the Medication Adherence Report Scale (MARS). Multidimensional relationships between clinical variables, WHO-5, and MARS were explored using correspondence analysis with symmetrical normalization. **Results:** Most patients had severe or very severe COPD (GOLD 3–4, 65.6%). WHO-5 scores showed marked heterogeneity across all GOLD stages, with very low values (20–26%) contributing disproportionately to the overall data structure (total contributions up to 0.97), indicating substantial anxiety and depression-related burden. Non-adherent patients represented 28.4% of the cohort but accounted for 71.6% of the inertia in the adherence-related correspondence analysis, forming a distinct psychological and behavioral profile. Mean GOLD stage was comparable between adherent and non-adherent patients (approximately 2.8–3.0). **Conclusions:** Correspondence analysis demonstrated substantial heterogeneity in WHO-5 and MARS profiles across all GOLD stages. Notably, mean GOLD severity was comparable between adherent and non-adherent patients (approximately 2.8–3.0), indicating that differences in adherence behavior were not primarily driven by spirometric disease severity.

## 1. Introduction

Chronic obstructive pulmonary disease is a progressive respiratory condition characterized by persistent airflow limitation, chronic symptoms, and a high burden of comorbidities. Beyond its pulmonary manifestations, COPD has increasingly been recognized as a systemic disease with substantial psychological consequences. Anxiety and depression are among the most prevalent and clinically relevant comorbidities in COPD, significantly affecting symptom perception, treatment adherence, quality of life, and long-term outcomes [1,2].

Patients with COPD frequently experience dyspnea, exercise intolerance, and recurrent exacerbations, which contribute to emotional distress and maladaptive coping strategies. Psychological symptoms may emerge early in the disease course and tend to intensify with increasing disease severity, functional limitation, and comorbidity burden [3]. Importantly, anxiety and depression are associated with poorer participation in pulmonary rehabilitation, reduced adherence to inhaled therapy, and increased healthcare utilization [4].

Recent evidence suggests that integrated disease management and tailored psychological interventions can improve both mental health outcomes and overall disease control in COPD patients [5,6]. However, anxiety and depression remain underdiagnosed and undertreated in routine clinical practice.

The assessment of psychological well-being and treatment adherence represents a critical component of comprehensive COPD management. The World Health Organization–Five Well-Being Index is a validated, patient-reported outcome measure designed to capture subjective well-being and to screen for depressive symptoms, with lower scores indicating impaired emotional health [7]. The raw score is calculated by totaling the figures of the five answers (Table S2 from Supplementary Material S1). The raw score ranges from 0 to 25, with 0 representing worst possible and 25 representing best possible quality of life. To obtain a percentage score ranging from 0 to 100, the raw score is multiplied by 4. A percentage score of 0 represents worst possible, whereas a score of 100 represents best possible quality of life [8].

In COPD populations, WHO-5 has demonstrated wide score dispersion, reflecting substantial heterogeneity in psychological status even among patients with comparable levels of airflow limitation [8]. Reduced WHO-5 scores have been associated with increased symptom burden, functional limitation, and poorer perceived health status [9].

Medication adherence, evaluated using the Medication Adherence Report Scale, provides complementary insight into patient behavior and self-management capacity. MARS captures intentional and unintentional non-adherence patterns, which are particularly relevant in COPD due to complex inhaler regimens and symptom-driven medication use [10].

This is a 5-item self-report tool where patients rate behaviors on a 5-point Likert scale (1 = Always to 5 = Never). Scores range from 5 to 25, with higher scores indicating better adherence. Common scoring includes Excellent/Good (25 or ≥24 in some studies), Moderate (20–25), and Poor (≤20 or ≤19) [10] (Table S2 from Supplementary Material S1).

In the analyzed datasets, adherence status was heterogeneous and not strictly dependent on GOLD stage; thus, behavioral and psychological factors play a substantial role in treatment implementation [11]. Importantly, lower adherence scores frequently coexisted with reduced WHO-5 values, supporting the bidirectional relationship between psychological distress and suboptimal disease management. Together, WHO-5 and MARS offer a multidimensional framework for identifying vulnerable COPD patients who may benefit from integrated psychological and behavioral interventions [12].

**Figure 1:**
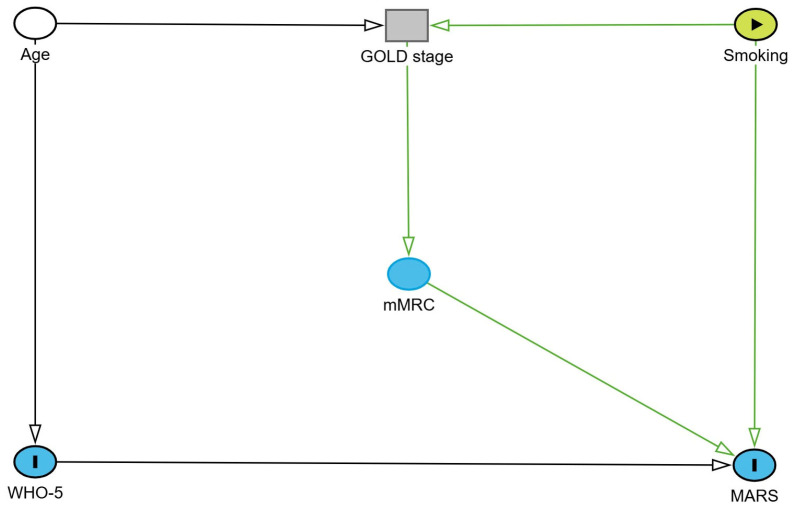
Directed acyclic graph illustrating the conceptual relationships between COPD severity, psychological well-being, symptom burden, treatment adherence, and key confounders. Created with https://dagitty.net/ (accessed on 1 February 2026).

Figure 1 shows a directed acyclic graph (DAG), which represents the hypothesized relationships between age, smoking status, COPD severity, symptom burden (mMRC), psychological well-being (WHO-5), and treatment adherence (MARS), based on clinical reasoning and the existing literature.

The diagram was developed a priori to guide covariate selection and interpretation of multivariable analyses.

The aim of this study was to investigate the relationship between COPD severity, functional impairment, psychological well-being, and treatment adherence in this cohort of patients with COPD. Specifically, we sought to explore how anxiety and depression-related symptoms, assessed using the WHO-5 Well-Being Index, and medication adherence, evaluated through the Medication Adherence Report Scale, are distributed across GOLD stages and relate to clinical indicators such as dyspnea, airflow limitation, and smoking exposure. By applying correspondence analysis, this study aimed to identify structured patterns linking disease severity with psychological burden and adherence behavior, thereby highlighting clinically relevant patient profiles. Ultimately, the study seeks to support a more integrated, patient-centered approach to COPD management that incorporates systematic psychological assessment alongside traditional respiratory evaluation.

## 2. Materials and Methods

### 2.1. Study Design and Population

This multicenter observational study was conducted across two primary care clinics (Constanța Pneumophthisiology Hospital “Palazu Mare” and County Emergency Hospital “Sf. Apostol Andrei” Constanta where Medical Clinic 1, and Medical Clinic 2 are located). A total of 285 patients, covering the period 2020–2025, with a confirmed diagnosis of chronic obstructive pulmonary disease were included. The study was conducted in accordance with the Declaration of Helsinki and approved by the Ethics Committee of Constanța Pneumophthisiology Hospital “Palazu Mare” protocol code 2400/21 March 2023 and County Emergency Hospital “Sf. Apostol Andrei” protocol code 24907/26 April 2023. This was a multicenter retrospective observational study. Data were collected from routinely recorded clinical evaluations, and no protocol-driven interventions or modifications to standard care were applied. All assessments reflected real-world clinical practice.

### 2.2. Inclusion and Exclusion Criteria

Inclusion criteria

Patients were eligible for inclusion if they met all of the following criteria: (1) age ≥ 18 years; (2) confirmed diagnosis of chronic obstructive pulmonary disease, established according to GOLD criteria based on spirometric evidence of persistent airflow limitation (post-bronchodilator FEV_1_/FVC < 0.70); (3) clinical stability at the time of assessment, defined as absence of acute exacerbation requiring hospitalization or systemic corticosteroid therapy; and (4) availability of complete clinical, functional, and questionnaire-based data, including spirometry, mMRC, CAT, WHO-5, and MARS scores.

Exclusion criteria

Patients were excluded if they had: (1) respiratory diseases other than COPD as the primary diagnosis, including asthma, interstitial lung disease, bronchiectasis as the dominant condition, pulmonary fibrosis, or active pulmonary tuberculosis; (2) acute COPD exacerbation at the time of evaluation; (3) severe cognitive impairment or psychiatric conditions precluding reliable completion of patient-reported outcome measures; (4) incomplete or missing data for key study variables; or (5) inability or refusal to provide informed consent.

### 2.3. Clinical and Functional Assessment

COPD severity was classified according to the Global Initiative for Chronic Obstructive Lung Disease criteria [13], based on spirometric evaluation. Forced expiratory volume in one second (FEV_1_), forced vital capacity (FVC), and the FEV_1_/FVC ratio were recorded for all patients [14]. Dyspnea severity was assessed using the modified Medical Research Council (mMRC) scale [15], while symptom burden was evaluated using the COPD Assessment Test (CAT) [16]. Smoking exposure was quantified using smoking status and cumulative exposure expressed as pack-years.

### 2.4. Psychological Well-Being and Treatment Adherence

Psychological well-being was evaluated using the World Health Organization–Five Well-Being Index, expressed as percentage scores, with lower values indicating impaired well-being and a higher likelihood of anxiety and depressive symptoms. Treatment adherence was assessed using the Medication Adherence Report Scale, categorizing patients as adherent or non-adherent.

### 2.5. Statistical Analysis

Descriptive statistics were used to summarize demographic characteristics, comorbidities, clinical variables, and questionnaire scores. Distribution normality was assessed using skewness and kurtosis. Associations between categorical variables were explored using correspondence analysis with symmetrical normalization, allowing visualization of multidimensional relationships between GOLD stages, symptom scores, adherence status, and psychological well-being. Correlation analyses were performed to assess relationships between GOLD stage, mMRC, WHO-5, and MARS. A random-effects meta-regression model (REML method) was applied to explore trends between GOLD severity and key clinical and behavioral variables. Statistical analyses were conducted using SPSS version 29, with a significance level set at *p* < 0.05.

## 3. Results

### 3.1. Population Characteristics

Table 1 presents the distributional characteristics of the main demographic, clinical, and comorbidity-related variables in the study cohort. For all variables, complete data were available, with no missing values recorded. The table summarizes measures of skewness and kurtosis for each variable [17], providing an overview of data distribution and normality assumptions. Other population characteristics are included in Supplementary Material S2.

The tables include 285 patients, with no missing values for any variable, indicating a complete and reliable database for subsequent statistical analyses. The analyzed variables encompass demographic data, comorbidities, and COPD severity indicators. The absence of missing data eliminates the risk of exclusion-related bias, supporting the validity of the results.

Most variables show skewness values between −1 and +1 (for example, age −0.328, obesity 0.471, diabetes 0.677), indicating relatively balanced distributions close to normality. This suggests that these characteristics are fairly uniformly distributed within the studied population and can be analyzed using standard statistical methods. In contrast, GOLD stage (skewness = 6.708), pulmonary embolism (2.414), and atrial fibrillation/flutter (1.961) display marked positive skewness, indicating that most patients fall into a dominant category, with a smaller number of severe or rare cases.

The very high kurtosis observed for the GOLD stage (43.298) indicates a marked clustering of patients within specific COPD severity stages, reflecting the clinical structure of the study cohort. Similarly, pulmonary embolism (kurtosis = 3.856) and atrial fibrillation/flutter (1.859) exhibit “peaked” distributions, which are characteristic of relatively rare clinical events. In contrast, the negative kurtosis values observed for obesity (−1.791) and diabetes (−2.014) indicate more uniform distributions of these comorbidities. More case report summaries are included in Tables S1 and S2 from Supplementary Material S3.

The distribution of patients according to the clinic of origin shows that the majority were recruited from Medicala 2, with 122 cases (42.8%), followed by the Palazu clinic with 113 patients (39.6%), while Medicala 1 was represented by 50 patients (17.5%) (data contained in Tables S1–S12 from Supplementary Material S2). This distribution indicates a predominant contribution from the Medicala 2 and Palazu clinics to the study cohort, with a higher volume of COPD patients hospitalized or monitored in these units.

The age distribution shows that the cohort is dominated by elderly patients, with the majority concentrated between 56 and 75 years of age, an interval that includes more than two-thirds of the total cases. The most frequent ages were 68 years (18 patients, 6.3%), 73 years (14 patients, 4.9%), and 67, 69, 70, and 71 years (13 patients each, approximately 4.6%). Younger patients were poorly represented, with only isolated cases under the age of 50 (each accounting for less than 1.5%) (Figures S1–S11 from Supplementary Material S2).

The sex distribution highlights a clear predominance of male patients, who account for 76.2% of the cohort (217 patients), while females represent 23.8% (68 patients) (Figures S1–S11 from Supplementary Material S2).

Most patients are classified as GOLD 3, with 106 cases (37.2%), followed by GOLD 4, with 81 patients (28.4%), indicating that nearly two-thirds of the cohort (65.6%) have severe or very severe COPD. Milder stages are less represented, with 69 patients in GOLD 2 (24.2%) and only 29 in GOLD 1 (10.2%) (data contained in Tables S1–S12 from Supplementary Material S2).

### 3.2. COPD and Smoking Status

The correspondence table (Table 2) between GOLD stage and smoking status shows that the majority of patients are smokers, with 267 out of 285 patients, while only 18 patients are non-smokers.

Across all GOLD stages, smokers predominate; in GOLD 1, 18 out of 29 patients are smokers; in GOLD 2, 68 out of 69; in GOLD 3, 102 out of 106; and in GOLD 4, 79 out of 81. Non-smokers are few and are mainly distributed in GOLD 1 (11 cases), with minimal representation in GOLD stages 2–4.

Table 3 and Table 4 summarize the mass, dimensional coordinates, inertia, and relative contributions of each GOLD stage/pack-years index to the overall correspondence analysis structure, indicating how different levels of COPD severity contribute to the separation along the main analytical dimensions.

**Figure 2:**
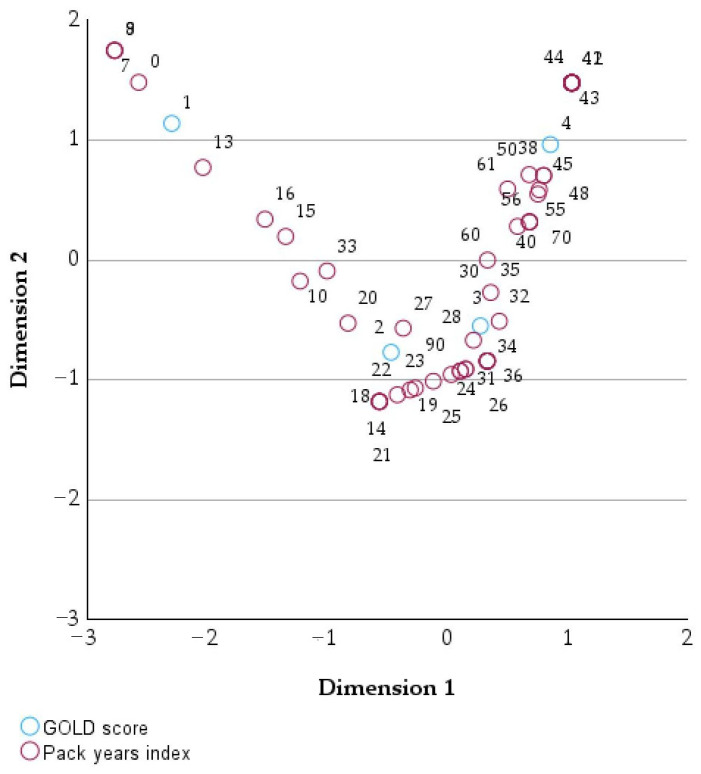
Two-dimensional correspondence analysis of GOLD stage and pack-years index.

The graphical representation in Figure 2 shows the two-dimensional correspondence analysis illustrates the simultaneous positioning of GOLD categories and pack-years index values according to the similarity of their profiles [18].

Dimension 1 clearly separates low pack-years values (0–10), located on the left side of the plot, from high values (≥40), grouped on the right, while intermediate values are positioned closer to the center. The GOLD categories are distributed along the same gradient, with GOLD 1 located in the area corresponding to low pack-years values and GOLD 3–4 positioned closer to regions associated with higher cumulative smoking exposure. Dimension 2 provides additional separation for certain extreme values, although with a smaller contribution than Dimension 1.

Table 5 and Table 6 present the mass, dimensional coordinates, inertia, and relative contributions of GOLD stages and mMRC categories, respectively, to the correspondence analysis.

**Figure 3:**
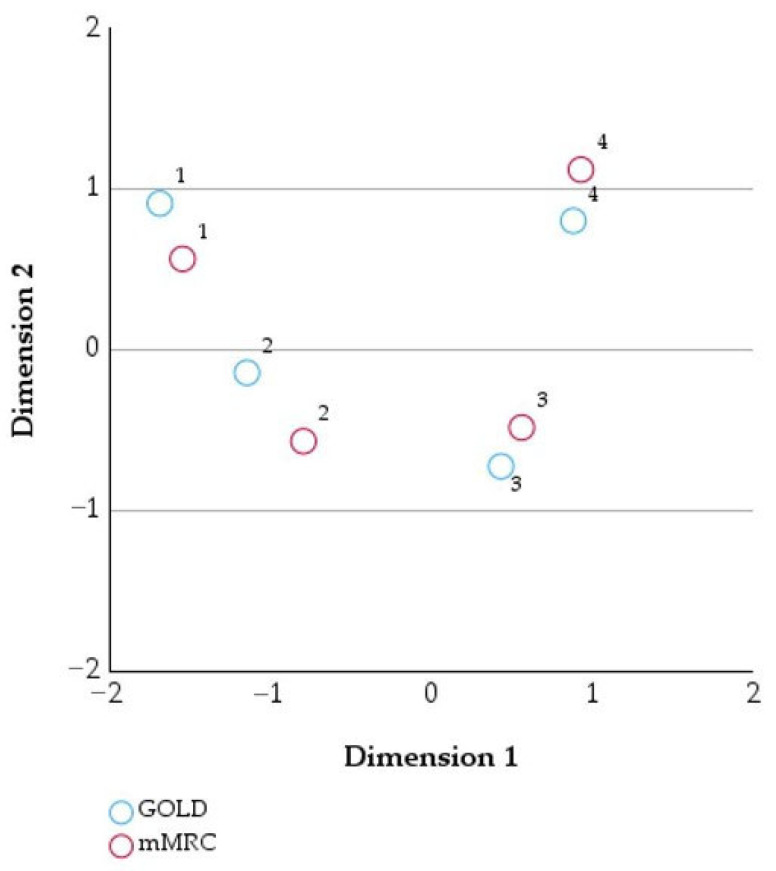
Two-dimensional correspondence analysis of GOLD stage and mMRC score (symmetrical normalization).

The graphical representation of the correspondence analysis (Figure 3) with symmetrical normalization shows a coherent and orderly distribution of the GOLD categories and mMRC scores across the two dimensions.

Along Dimension 1, a clear separation can be observed between the lower categories (GOLD 1–2 and mMRC 1–2), located on the left side of the plot, and the higher categories (GOLD 3–4 and mMRC 3–4), positioned on the right. Along Dimension 2, GOLD 1 and mMRC 1 are located in the upper part of the plot, GOLD 2 and mMRC 2 are close to the central axis, and GOLD 3 and mMRC 3 are positioned in the lower part, while GOLD 4 and mMRC 4 are again found in the upper region. The spatial proximity between categories with the same numerical level (1 with 1, 2 with 2, 3 with 3, and 4 with 4) indicates a clear correspondence between GOLD stage and mMRC score within the data structure, without overlaps or atypical positioning.

Table 7 and Table 8 present the mass, dimensional coordinates, inertia, and relative contributions of GOLD stages and FEV_1_/FVC categories in the correspondence analysis.

**Figure 4:**
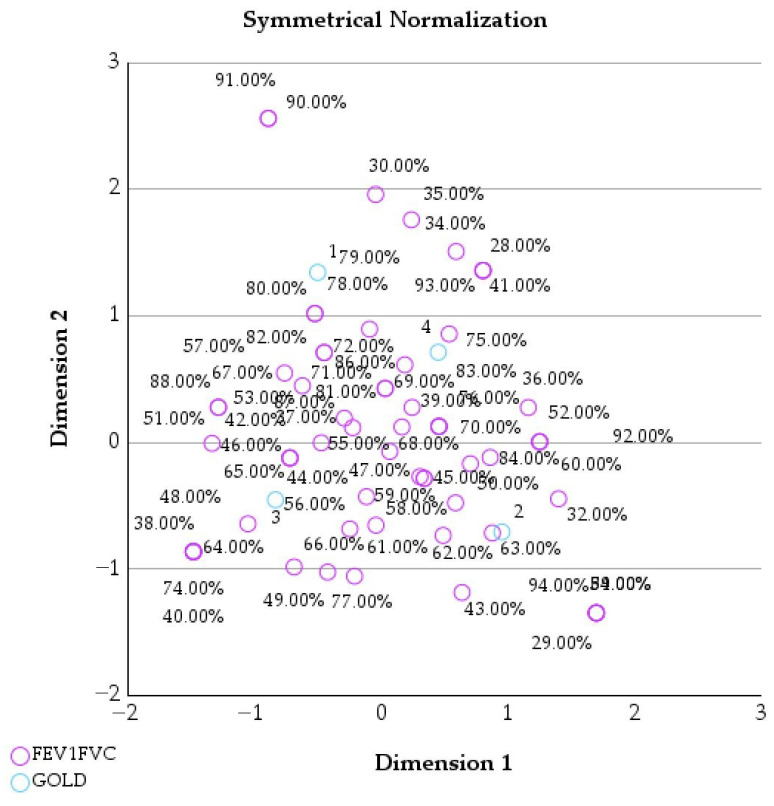
Joint correspondence analysis of GOLD stages and FEV1/FVC values (symmetrical normalization).

Figure 4 displays the joint correspondence analysis between GOLD stages and FEV1/FVC values, illustrating how airflow limitation relates to disease severity in the study cohort.

Lower FEV1/FVC values tend to cluster closer to higher GOLD stages, indicating more severe airflow obstruction as disease severity increases.

### 3.3. WHO-5, MARS and GOLD Stage

Table 9 summarizes the contribution of each GOLD stage to the correspondence analysis, illustrating how different levels of COPD severity shape the overall structure of the data. It provides insight into the relative influence of mild, moderate, and severe disease stages on the clinical patterns associated with psychological burden in COPD.

Patients in GOLD stage 1 represent a small proportion of the cohort (mass = 0.095) and have scores very close to zero on both dimensions (−0.048 on Dimension 1 and −0.092 on Dimension 2). Their total contribution to the overall structure is minimal (total contribution = 0.010), meaning that patients with mild COPD do not strongly influence the global pattern observed in the data. Clinically, this suggests that GOLD 1 patients are relatively homogeneous, with limited symptom burden and functional impairment, and therefore a lower and more uniform impact on anxiety and depressive symptoms.

In contrast, GOLD stage 2 patients show a marked differentiation. They account for almost a quarter of the cohort (mass = 0.233) and are clearly separated from the mild stage, with a strong negative position on Dimension 1 (−0.871) and a positive position on Dimension 2 (0.558). Importantly, GOLD 2 has an extremely high total contribution (0.992), indicating that this stage plays a central role in shaping the overall structure of the analysis. From a psychological perspective, this suggests that GOLD 2 represents a key transition point at which increasing symptoms and functional limitations begin to significantly affect emotional well-being.

GOLD stage 3 includes the largest proportion of patients (mass = 0.389) and is mainly differentiated along Dimension 2 (score = −0.614). Although its influence on Dimension 1 is minimal (contribution = 0.004), GOLD 3 contributes very strongly to Dimension 2 (contribution = 0.939), with a high total contribution (0.953). Clinically, this reflects a stage characterized by pronounced dyspnea, reduced physical capacity, and persistent symptoms. These features are closely linked to anxiety related to breathlessness and depressive symptoms associated with loss of autonomy and reduced quality of life.

Patients in GOLD stage 4 also show a strong and distinct profile. They represent a substantial part of the cohort (mass = 0.282) and are clearly positioned on the positive side of Dimension 1 (score = 0.823) and Dimension 2 (0.418). GOLD 4 has a very high total contribution (0.994), indicating a major influence on the overall data structure. This strong differentiation reflects the advanced and disabling nature of end-stage COPD, where severe airflow limitation, frequent exacerbations, and dependence on supportive therapies are likely to be accompanied by a high psychological burden, including elevated levels of anxiety and depression (Figure 4).

**Figure 5:**
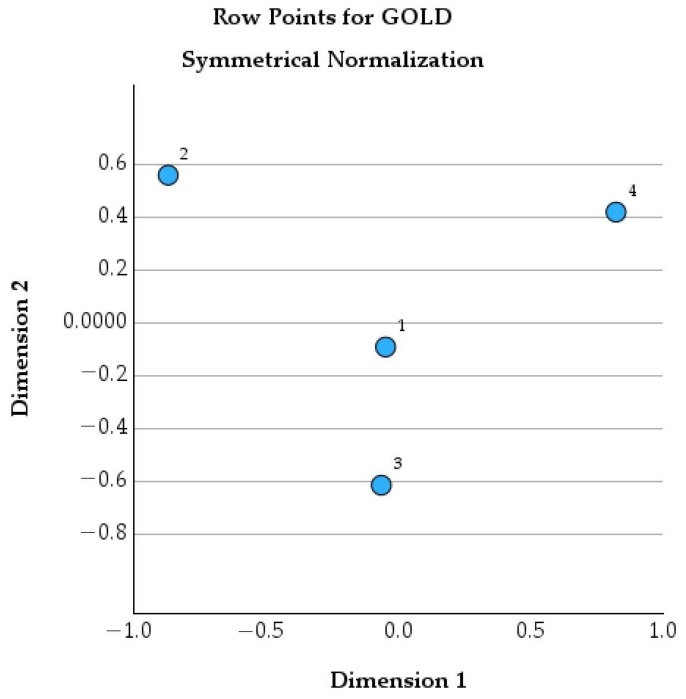
Row points for GOLD stages in the correspondence analysis (symmetrical normalization).

Figure 5 shows the correspondence analysis map for the GOLD classification using symmetrical normalization

Table 10 presents the contribution of different WHO-5 percentage scores to the correspondence analysis.

The WHO-5 scores show heterogeneous contributions to the overall structure of the analysis. Very low WHO-5 values, such as 20% (mass = 0.031) and 26% (mass = 0.023), are positioned far from the center of the analysis (Dimension 1 scores of 1.031 and −0.858, respectively) and have high total contributions (0.969 and 0.878). Thus, patients with markedly reduced well-being strongly shape the pattern of psychological distress.

Intermediate WHO-5 values, such as 40% (mass = 0.160) and 48% (mass = 0.080), also contribute substantially to the structure (total contributions of 0.942 and 0.818, respectively), reflecting a large subgroup of patients with moderate impairment in emotional well-being. This group represents patients with persistent symptoms and functional limitation who experience ongoing psychological strain without reaching the most severe levels of distress.

Higher WHO-5 scores, including 52% (mass = 0.084) and 56% (mass = 0.046), are mainly differentiated along Dimension 2, with high contributions to this dimension (100% and 74.0%, respectively). However, even these scores still contribute meaningfully to the overall inertia, highlighting that psychological well-being in COPD exists along a continuum rather than as a separation.

Extremely high or low WHO-5 scores, such as 42% and 70%, despite their small mass (0.004 each), show very high total contributions (0.977).

**Figure 6:**
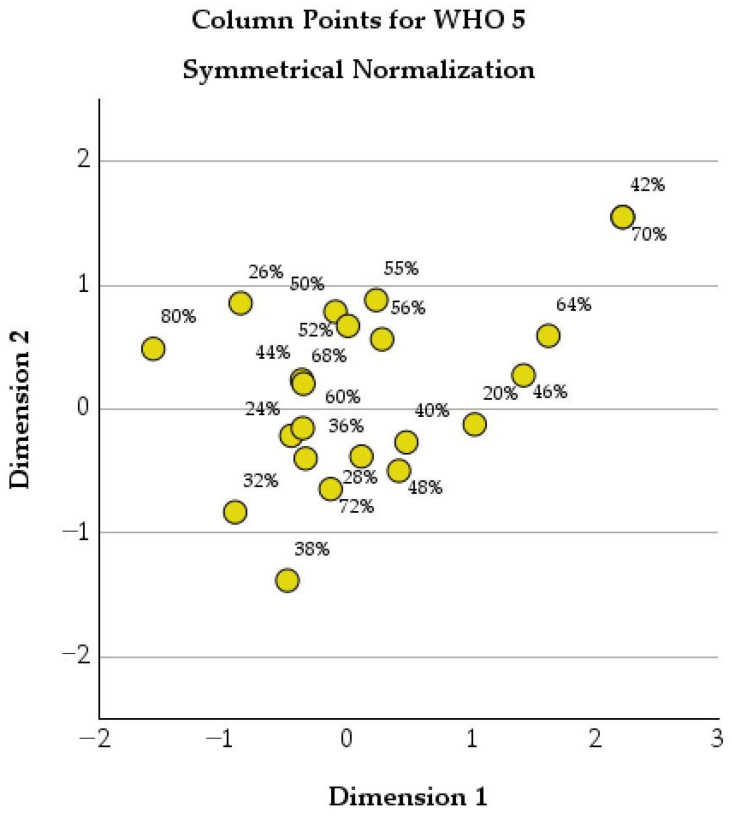
Two-dimensional correspondence analysis of WHO-5 percentage scores (symmetrical normalization).

Figure 6 below shows the two-dimensional correspondence analysis of WHO-5 percentage scores.

**Figure 7:**
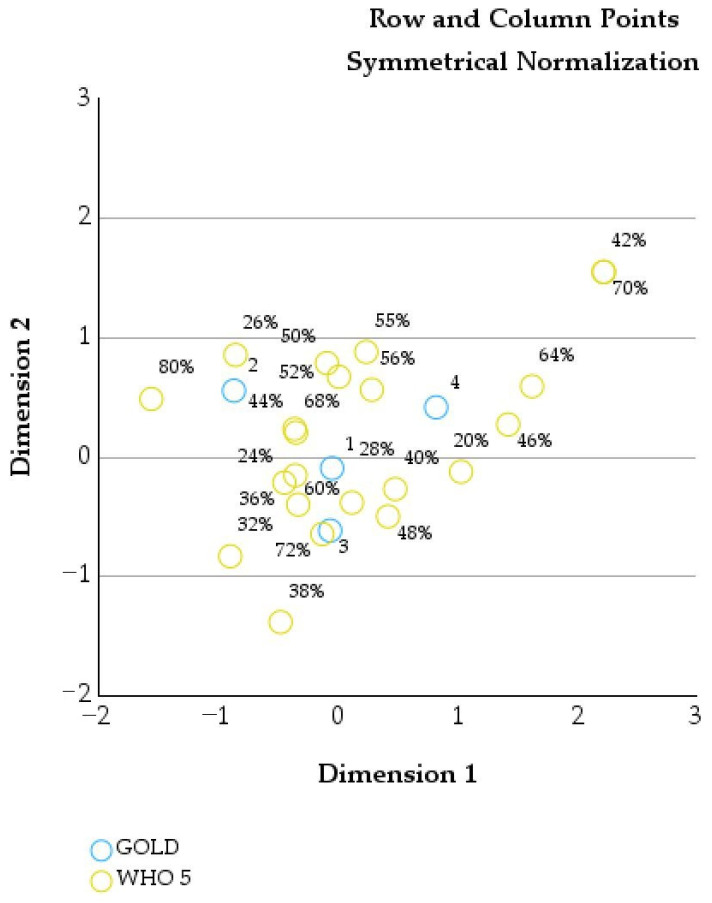
Joint correspondence analysis of GOLD stages and WHO-5 percentage scores (symmetrical normalization).

Figure 7 illustrates the joint correspondence analysis between GOLD stages and WHO-5 scores, showing how COPD severity relates to variations in patients’ psychological well-being and symptoms of anxiety and depression.

Patients with higher WHO-5 scores, indicating better emotional well-being and fewer depressive or anxiety symptoms, are grouped on one side of the graph. These higher scores (such as 42%, 64%, and 70%) tend to appear closer to GOLD stages 3 and 4. Hence, even among patients with more severe COPD, there is variability in psychological well-being and that not all severely affected patients experience the same level of emotional distress. Also, the presence of these scores near advanced GOLD stages highlights that emotional well-being becomes a more defining and structured aspect of patient experience as disease severity increases.

In contrast, GOLD stages 1 and 2 are positioned in areas associated with a broader spread of WHO-5 values, including lower scores. Patients with milder or moderate COPD show greater variability in emotional status, ranging from good well-being to significant psychological distress. Clinically, anxiety and depression can already be present early in the disease course and are not limited to advanced COPD.

The vertical spread of WHO-5 scores across the graph shows that patients within the same GOLD stage can have very different emotional experiences. This reflects individual differences in coping, symptom perception, comorbidities, and social factors [19].

For the MARS analysis, we summarized in Table 11 how each GOLD stage contributes to the single main dimension of the correspondence analysis. It illustrates the relative role of COPD severity in structuring the clinical–psychological profile of the study population.

This MARS one-dimensional correspondence analysis shows that GOLD 1 patients, although representing a small proportion of the cohort (mass = 0.102), have the strongest separation from the other stages (score = −0.579) and contribute most to the overall structure (63.4% of inertia). This indicates a distinct clinical and psychological profile. GOLD 2 also contributes meaningfully (22.4% of inertia; score = 0.223). This is a transition stage where symptom burden and psychological distress begin to increase. In contrast, GOLD 3 and GOLD 4, which together represent the majority of patients (mass = 0.656), cluster near the center (scores 0.102 and −0.116) with low individual contributions (7.1% and 7.0%), indicating a relatively uniform and consistently elevated burden of anxiety and depression-related symptoms in advanced COPD.

**Figure 8:**
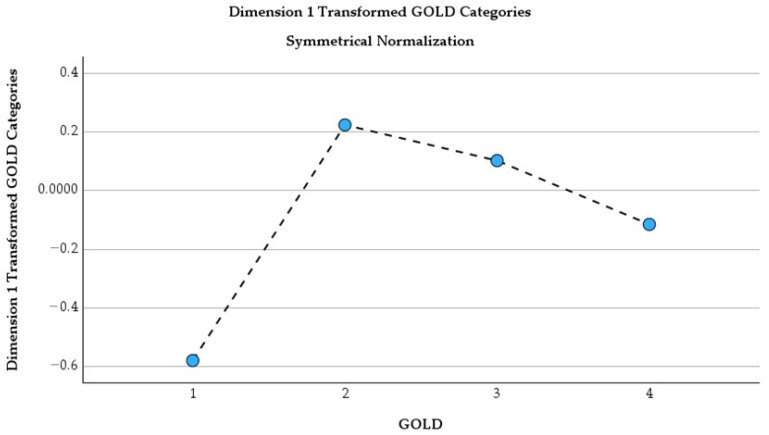
Dimension 1 transformed GOLD categories (symmetrical normalization).

Figure 8 shows a clear progression of GOLD stages along Dimension 1, with GOLD 1 positioned at the negative extreme (approximately −0.58), indicating a distinct profile compared with the other stages. GOLD 2 shifts sharply toward positive values (around 0.22). GOLD 3 and GOLD 4 move gradually back toward lower values (~0.10 and −0.12), indicating that in advanced COPD, the psychological impact, including anxiety and depressive symptoms, becomes more homogeneous across severe stages rather than progressively worsening.

Table 12 summarizes the contribution of treatment adherence status, as measured by the MARS scale, to the correspondence analysis. This scale is clinically and psychologically differentiating the profiles among our patients.

A distinct behavioral and clinical profile is represented by this one-dimensional correspondence analysis (Table 1) that shows a clear separation between non-adherent and adherent patients based on MARS. Non-adherent patients, who represent 28.4% of the cohort, are positioned at the negative end of the dimension (score = −0.368) and account for the majority of the explained structure (71.6% of inertia). In contrast, adherent patients (71.6% of the cohort) cluster closer to the center (score = 0.146) with a smaller contribution (28.4% of inertia). Consequently, non-adherence is a key differentiating factor and may be closely linked to higher psychological burden, including anxiety and depressive symptoms, in COPD patients.

**Figure 9:**
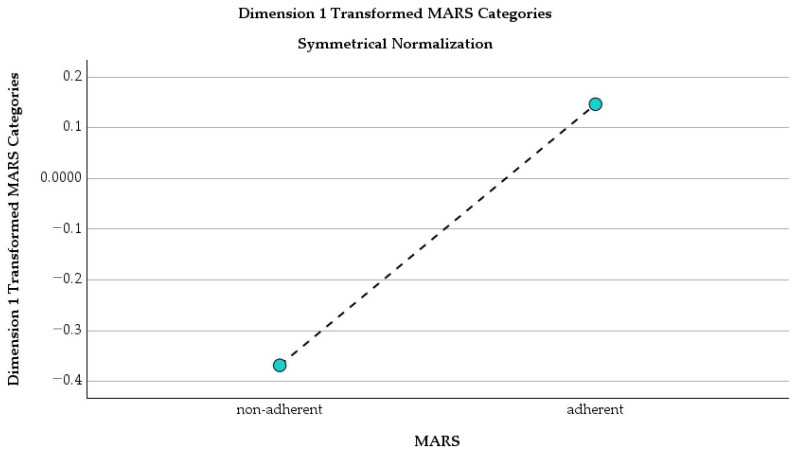
Dimension 1 transformed MARS categories (symmetrical normalization).

Figure 9 illustrates the separation between adherent and non-adherent patients along the main dimension of the correspondence analysis. We can go further to say that the treatment adherence is shaping patient profiles.

Non-adherent and adherent patients are distinctive groups due to their position at the negative end of Dimension 1 (approximately −0.37), while adherent patients are located at the positive end (~0.15). So, non-adherent individuals have a distinct clinical and psychological profile, which includes higher levels of anxiety and depressive symptoms, while adherent ones are linked to more stable disease management and better psychological well-being in patients with COPD [20].

### 3.4. Multivariable, Interaction Analysis and Model Assumptions

A multivariate logistic regression model was constructed to evaluate the independent associations between psychological well-being (WHO-5), COPD severity (GOLD stage), and key clinical variables with treatment adherence (Figure S1 and Tables S1–S19 from Supplementary Material S4).

In the multivariate logistic regression model including an interaction term, neither centered psychological well-being (WHO5_c) nor centered COPD severity (GOLD_c) showed a statistically significant independent association with treatment adherence. Importantly, the interaction between WHO-5 score and GOLD stage was not significant (*p* = 0.594). Demographic variables (age and sex) and spirometric impairment (FEV_1_/FVC) were also not significant predictors in the adjusted model. Dyspnea severity, assessed by the mMRC scale, demonstrated a borderline inverse association with adherence (OR = 0.64, *p* = 0.061). The full analysis is included in Figure 1 and Tables S1–S19 from Supplementary Material S4. Age was independently associated with higher COPD severity (β = −0.17, *p* = 0.004), indicating an association between increasing age and more advanced disease stages. Lung function, expressed as the FEV_1_/FVC ratio, showed a borderline inverse association with GOLD stage (*p* = 0.059). Symptom burden (CAT score), treatment adherence, and psychological well-being were not significantly associated with COPD severity in the adjusted model. Collinearity diagnostics indicated acceptable tolerance and variance inflation factor values for all predictors.

Collinearity diagnostics were performed to assess potential multicollinearity among predictors included in the multivariable regression model.

The condition indices remained below critical thresholds for severe multicollinearity, and the variance proportions were not simultaneously high (>0.50) for multiple predictors within the same dimension. Although a higher condition index was observed in the final dimension, the associated variance proportions were primarily attributable to the intercept rather than to substantive predictors.

**Figure 10:**
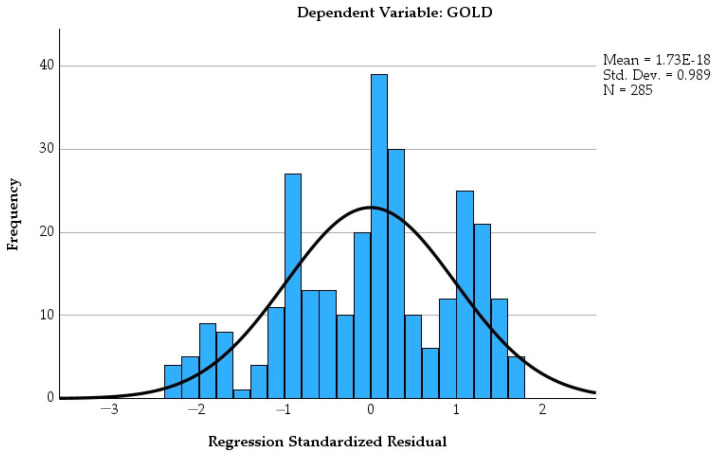
Histogram of standardized residuals for the multivariable regression model with GOLD stage as the dependent variable.

Figure 10 presents the histogram of regression standardized residuals for the dependent variable GOLD.

The histogram of standardized residuals demonstrates an approximately symmetric distribution centered around zero, with no marked deviations from normality. Residuals are reasonably normally distributed due to the overlaid normal curve. No extreme outliers were observed.

**Figure 11:**
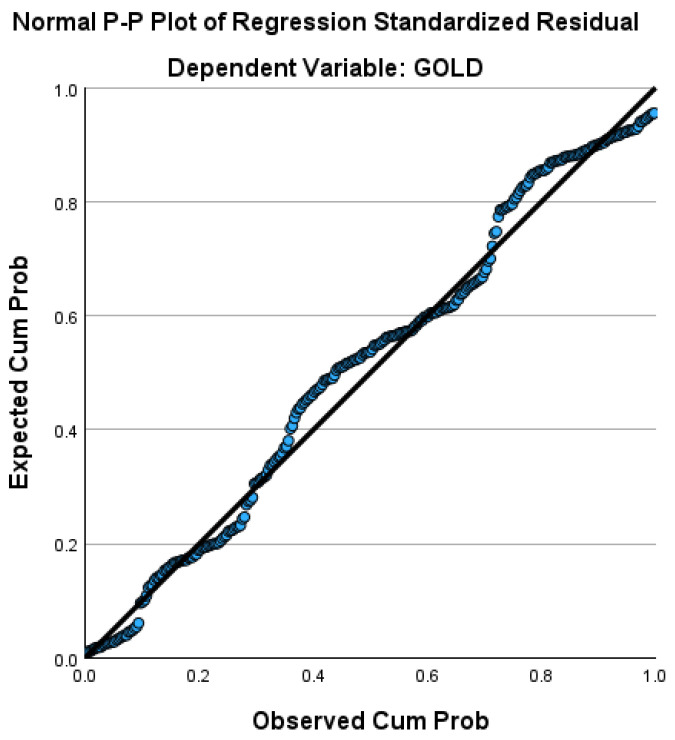
Normal P–P plot of standardized residuals for the multivariable regression model with GOLD stage as the dependent variable.

Figure 11 shows the normal P-P plot of regression.

The normal P–P plot shows that the standardized residuals closely follow the diagonal reference line, indicating an approximately normal distribution of residuals across the range of observed values. Minor deviations at the distribution tails are minimal and do not suggest substantial departures from normality.

**Figure 12:**
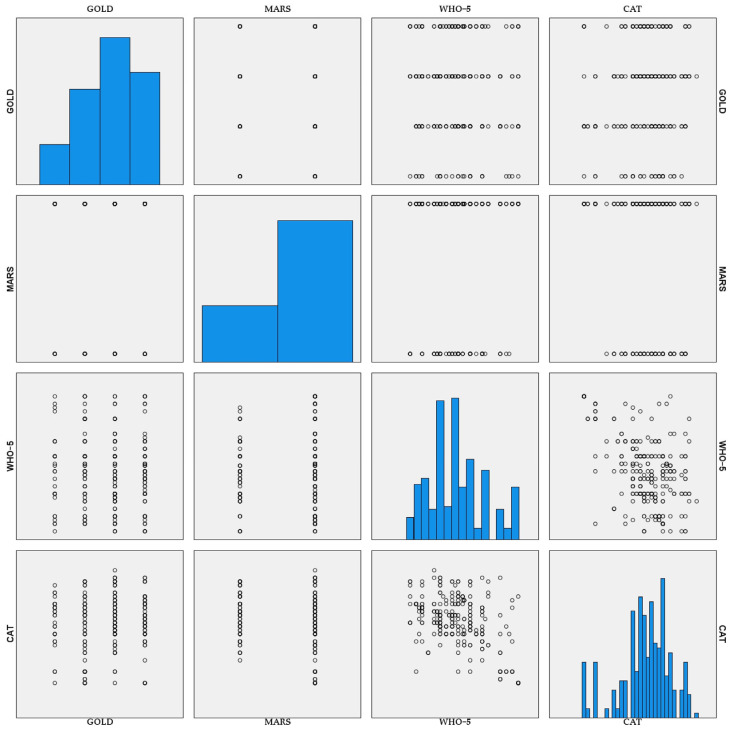
Scatterplot matrix illustrating the relationships among GOLD stage, MARS, WHO-5, and CAT.

The scatterplot matrix included in Figure 12 provides an integrated visual overview of the pairwise relationships among disease severity, adherence behavior, psychological well-being, and symptom burden in the study cohort.

The distribution plots along the diagonal illustrate the variability in each variable, while the off-diagonal panels highlight heterogeneous patterns across combinations of clinical and patient-reported measures. Notably, WHO-5 and CAT scores show a broad dispersion across GOLD stages. In contrast, adherence categories appear discretely distributed across disease severity and symptom scores.

## 4. Discussion

The present study highlights the complex interplay between psychological well-being and treatment adherence in patients with COPD. Previous evidence, such as that presented by Zoeckler et al. [21] and discussed by Asnaashari et al. [22], indicates that depressive and anxiety symptoms are prevalent among individuals with COPD and contribute substantially to impaired quality of life and increased morbidity. Depression and anxiety in COPD patients are challenging to identify due to overlapping respiratory and mood symptoms, yet their presence has been shown to increase fatigue, reduce physical functioning, and elevate healthcare utilization. Our findings add to this body of work by demonstrating that psychological well-being, as measured by WHO-5, and MARS scores are important dimensions of COPD burden that extend beyond spirometric severity.

In this research, we identified a structured relationship between disease severity, psychological well-being, and treatment adherence. The predominance of severe disease (GOLD 3–4 accounting for 65.6% of cases) was associated with a relatively homogeneous and consistently elevated psychological burden, as reflected by the correspondence analysis, where GOLD 3 and GOLD 4 clustered near the center with low individual contributions (7.1% and 7.0%, respectively). This finding supports previous reports indicating that anxiety and depression become pervasive once COPD reaches advanced stages, rather than increasing linearly with airflow limitation [23,24].

WHO-5 scores showed marked heterogeneity across all GOLD stages, with very low values (20–26%) contributing disproportionately to the overall structure (total contributions up to 0.97). Similar to Benso et al., reduced well-being was not confined to advanced COPD [23]. The wide distribution of WHO-5 scores within each GOLD stage further emphasizes the role of individual vulnerability, symptom perception, and coping mechanisms [25].

Clinically, these results have important implications for COPD management. As under-recognised anxiety and depression have been linked to poorer treatment adherence and worse functional outcomes, routine screening for psychological distress should be integrated into COPD care pathways to improve both mental health and adherence to pharmacological and non-pharmacological interventions [26]. Although some moderate evidence suggests relationships between psychological status and COPD severity, as reflected in prior work by Funk et al. [27] on psychological status in mixed airway disease populations, the present analysis emphasizes that such associations may not solely depend on airflow limitation.

Treatment adherence assessed by MARS revealed that non-adherent patients represented 28.4% of the cohort but accounted for 71.6% of the inertia in the correspondence analysis. This finding is consistent with prior studies reporting an association between non-adherence, anxiety, and depressive symptoms in COPD [25,26]. Notably, GOLD stage did not differ substantially between adherent and non-adherent patients (mean GOLD ≈ 2.8–3.0), reinforcing the concept that psychological and behavioral factors, rather than disease severity alone, drive adherence patterns.

The incorporation of validated mental health measures alongside traditional biomarkers and staging systems may enable early identification of at-risk individuals and prompt holistic management strategies that address both physical and psychological determinants of health [28,29]. Future research should build on these observations to test tailored interventions and clarify causal pathways linking psychological distress, adherence, and COPD outcomes in longitudinal cohorts.

## 5. Strengths and Limitations

This study has several strengths that support the relevance of the findings for clinical practice. The multicenter design and the inclusion of a relatively large cohort of patients with COPD provide a representative overview of disease severity encountered in routine care. The use of validated patient-reported outcome measures, the WHO-5 Well-Being Index and MARS, enabled standardized assessment of psychological well-being and treatment adherence. In addition, the absence of missing data across the analyzed variables enhances the robustness of the statistical analyses. The application of correspondence analysis further represents a methodological strength, allowing exploration of multidimensional associations between clinical severity, psychological status, and adherence behavior in an intuitive and interpretable manner.

Several limitations should be considered when interpreting the results. The observational and cross-sectional design does not allow causal inference and limits conclusions regarding temporal relationships between psychological well-being, adherence, and disease severity. Although multivariate and interaction analyses were conducted to complement the correspondence analysis, residual confounding by unmeasured factors, such as treatment complexity, exacerbation history, or socioeconomic status, cannot be excluded. Treatment adherence was assessed using a self-reported questionnaire, which may be influenced by recall or social desirability bias. Consequently, the sample size was determined by feasibility rather than by hypothesis-driven power estimation. Although the final cohort of 285 patients is considered adequate for correspondence analysis and multivariate exploratory techniques, this should be regarded as a methodological limitation, and the findings should be interpreted as hypothesis-generating rather than confirmatory.

## 6. Conclusions

Psychological well-being and treatment adherence emerged as key dimensions of disease burden. WHO-5 scores demonstrated marked heterogeneity across all GOLD stages, with very low values (20–26%) exerting a disproportionate influence on the overall data structure (total contributions up to 0.97), highlighting the presence of significant anxiety and depression-related symptoms even in patients with similar spirometric severity. Although GOLD 3 and GOLD 4 accounted for the majority of cases (65.6%), psychological distress was not uniformly distributed.

Treatment adherence assessed by MARS further differentiated patient profiles. Non-adherent patients represented 28.4% of the cohort but accounted for 71.6% of the inertia in the correspondence analysis. This concludes that they have a distinct behavioral and psychological pattern. Notably, mean GOLD severity was comparable between adherent and non-adherent patients (approximately 2.8–3.0). Together, these findings support the routine integration of WHO-5 and MARS assessments into COPD management to identify vulnerable patients and guide targeted, patient-centered interventions.

## Figures and Tables

**Table 1:** Population characteristics.

				Clinic	Age	Sex	Obesity		COPD		GOLD		Cardiac Failure
*n*		Valid		285	285	285	285		285		285		285
		Missing		0	0	0	0		0		0		0
Median				2.05 ^a^	67.13 ^a^	1.24 ^a^	0.39 ^a^		.^a^		2.90 ^a^		0.40 ^a^
Variance				0.826	90.434	0.182	0.238		0.000		0.910		0.240
Skewness				−0.063	−0.328	1.233	0.471				−0.382		0.425
Std. Error of Skewness				0.144	0.144	0.144	0.144		0.144		0.144		0.144
Kurtosis				−1.793	0.280	−.483	−1.791				−0.807		−1.832
Std. Error of Kurtosis				0.288	0.288	0.288	0.288		0.288		0.288		0.288
Range				2	62	1	1		0		3		1
Minimum				1	34	1	0		1		1		0
Maximum				3	96	2	1		1		4		1
Percentiles		25		1.18 ^b^	58.91 ^b^	.^b,c^	.^b,c^		.^b^		2.09 ^b^		.^b,c^
		50		2.05	67.13	1.24	0.39		.		2.90		0.40
		75		2.88	72.40	1.74	0.89		.		3.67		0.90
		**BP**	**FEV1FVC**	**Smoker**	**Pack-Years** **Index**	**Smoking** **Cessation**	**SpO2**	**mMRC**	**O2 Therapy**		**CAT**	**MARS**	**WHO_5**
*n*	Valid	285	285	285	285	285	285	285	285		285	285	285
	Missing	0	0	0	0	0	0	0	0		0	0	0
Median		0.72 ^a^	61.0000% ^a^	0.94 ^a^	34.14 ^a^	0.16 ^a^	92.98% ^a^		2.69 ^a^	0.46 ^a^	24.16 ^a^	0.72 ^a^	49.24% ^a^
Variance		0.201	261.590	0.059	424.814	0.136	17.591		1.103	0.249	44.183	0.204	343.739
Skewness		−1.001	0.019	−3.611	0.484	1.850	−3.454		−0.486	0.177	−0.717	−0.962	0.498
Std. Error of Skewness		0.144	0.144	0.144	0.144	0.144	0.144		0.144	0.144	0.144	0.144	0.144
Kurtosis		−1.005	−0.943	11.116	−0.157	1.434	30.384		−0.628	−1.983	0.391	−1.082	−0.325
Std. Error of Kurtosis		0.288	0.288	0.288	0.288	0.288	0.288		0.288	0.288	0.288	0.288	0.288
Range		1	65.20%	1	100	1	47%		4	1	30	1	72%
Minimum		0	28.80%	0	0	0	52%		0	0	7	0	20%
Maximum		1	94.00%	1	100	1	99%		4	1	37	1	92%
Percentiles	25	0.22 ^b^	48.9750% ^b^	0.44 ^b^	22.70 ^b^	.^b,c^	90.53% ^b^	1.78 ^b^	.^b,c^		20.47 ^b^	0.22 ^b^	38.55% ^b^
	50	0.72	61.0000%	0.94	34.14	0.16	92.98%	2.69	0.46		24.16	0.72	49.24%
	75	.	75.5500%	.	50.93	0.66	95.35%	3.49	0.96		27.86	.	61.52%

^a^ Calculated from grouped data. ^b^ Percentiles are calculated from grouped data. ^c^ The lower bound of the first interval or the upper bound of the last interval is not known. Some percentiles are undefined.

**Table 2:** Distribution of smoking status across GOLD severity stages.

GOLD	Smoker		
	No	Yes	Active Margin
1	11	18	29
2	1	68	69
3	4	102	106
4	2	79	81
Active Margin	18	267	285

**Table 3:** Overview of row points for GOLD stages in the correspondence analysis (symmetrical normalization).

GOLD	Mass	Score in Dimension		Inertia	Contribution				
		1	2		of Point to Inertia of Dimension		of Dimension to Inertia of Point		
					1	2	1	2	Total
1	0.102	−2.297	1.141	0.543	0.648	0.203	0.818	0.159	0.977
2	0.242	−0.468	−0.769	0.265	0.064	0.220	0.166	0.352	0.518
3	0.372	0.276	−0.549	0.210	0.034	0.172	0.112	0.347	0.459
4	0.284	0.860	0.965	0.362	0.254	0.406	0.481	0.477	0.958
Active Total	1.000			1.380	1.000	1.000			

**Table 4:** Overview of column points for pack-years index in the correspondence analysis.

Pack-Years Index	Mass	Score in Dimension		Inertia	Contribution				
		1	2		of Point to Inertia of Dimension		of Dimension to Inertia of Point		
					1	2	1	2	Total
0	0.039	−2.572	1.484	0.276	0.308	0.130	0.766	0.200	0.966
7	0.004	−2.773	1.750	0.031	0.033	0.016	0.721	0.226	0.947
8	0.004	−2.773	1.750	0.031	0.033	0.016	0.721	0.226	0.947
9	0.004	−2.773	1.750	0.031	0.033	0.016	0.721	0.226	0.947
10	0.042	−1.226	−0.175	0.064	0.076	0.002	0.822	0.013	0.835
13	0.011	−2.037	0.773	0.040	0.053	0.010	0.898	0.102	1.000
14	0.007	−0.565	−1.180	0.022	0.003	0.015	0.085	0.290	0.374
15	0.032	−1.347	0.197	0.048	0.069	0.002	0.980	0.017	0.997
16	0.021	−1.519	0.341	0.042	0.059	0.004	0.960	0.038	0.999
18	0.011	−0.565	−1.180	0.033	0.004	0.022	0.085	0.290	0.374
19	0.025	−0.309	−1.083	0.033	0.003	0.044	0.059	0.576	0.636
20	0.018	−0.827	−0.526	0.017	0.014	0.007	0.574	0.183	0.756
21	0.007	−0.565	−1.180	0.022	0.003	0.015	0.085	0.290	0.374
22	0.021	−0.416	−1.123	0.041	0.004	0.041	0.074	0.423	0.497
23	0.032	−0.266	−1.067	0.036	0.003	0.055	0.052	0.654	0.706
24	0.018	0.154	−0.909	0.016	0.000	0.022	0.022	0.608	0.630
25	0.021	0.034	−0.954	0.014	0.000	0.029	0.001	0.908	0.909
26	0.018	0.154	−0.909	0.016	0.000	0.022	0.022	0.608	0.630
27	0.025	−0.367	−0.568	0.010	0.004	0.012	0.269	0.506	0.774
28	0.014	0.109	−0.926	0.011	0.000	0.018	0.013	0.725	0.738
29	0.014	0.109	−0.926	0.011	0.000	0.018	0.013	0.725	0.738
30	0.067	0.218	−0.668	0.032	0.004	0.046	0.082	0.606	0.688
31	0.007	0.333	−0.842	0.012	0.001	0.008	0.055	0.273	0.328
32	0.025	0.434	−0.510	0.026	0.006	0.010	0.149	0.162	0.311
33	0.011	−1.002	−0.090	0.009	0.013	0.000	0.978	0.006	0.984
34	0.011	0.333	−0.842	0.018	0.001	0.011	0.055	0.273	0.328
35	0.039	0.362	−0.270	0.008	0.006	0.004	0.548	0.240	0.788
36	0.011	0.333	−0.842	0.018	0.001	0.011	0.055	0.273	0.328
37	0.007	0.333	−0.842	0.012	0.001	0.008	0.055	0.273	0.328
38	0.011	0.803	0.706	0.009	0.008	0.008	0.620	0.377	0.996
40	0.063	0.586	0.281	0.021	0.026	0.008	0.843	0.153	0.996
41	0.004	1.038	1.480	0.009	0.005	0.012	0.354	0.567	0.921
42	0.004	1.038	1.480	0.009	0.005	0.012	0.354	0.567	0.921
43	0.004	1.038	1.480	0.009	0.005	0.012	0.354	0.567	0.921
44	0.007	1.038	1.480	0.018	0.009	0.024	0.354	0.567	0.921
45	0.046	0.767	0.587	0.033	0.032	0.024	0.667	0.307	0.975
46	0.004	1.038	1.480	0.009	0.005	0.012	0.354	0.567	0.921
47	0.004	1.038	1.480	0.009	0.005	0.012	0.354	0.567	0.921
48	0.011	0.803	0.706	0.009	0.008	0.008	0.620	0.377	0.996
49	0.004	1.038	1.480	0.009	0.005	0.012	0.354	0.567	0.921
50	0.046	0.683	0.713	0.039	0.026	0.036	0.456	0.391	0.847
52	0.004	1.038	1.480	0.009	0.005	0.012	0.354	0.567	0.921
53	0.004	1.038	1.480	0.009	0.005	0.012	0.354	0.567	0.921
55	0.018	0.756	0.551	0.012	0.012	0.008	0.680	0.284	0.964
56	0.007	0.686	0.319	0.004	0.004	0.001	0.706	0.120	0.826
58	0.004	1.038	1.480	0.009	0.005	0.012	0.354	0.567	0.921
60	0.077	0.336	0.000	0.016	0.011	0.000	0.451	0.000	0.451
61	0.011	0.504	0.593	0.011	0.003	0.006	0.205	0.224	0.430
62	0.004	1.038	1.480	0.009	0.005	0.012	0.354	0.567	0.921
63	0.004	1.038	1.480	0.009	0.005	0.012	0.354	0.567	0.921
65	0.014	1.038	1.480	0.035	0.018	0.047	0.354	0.567	0.921
68	0.004	1.038	1.480	0.009	0.005	0.012	0.354	0.567	0.921
70	0.042	0.686	0.319	0.023	0.024	0.007	0.706	0.120	0.826
78	0.011	1.038	1.480	0.027	0.014	0.035	0.354	0.567	0.921
80	0.021	0.686	0.319	0.012	0.012	0.003	0.706	0.120	0.826
86	0.004	−0.565	−1.180	0.011	0.001	0.007	0.085	0.290	0.374
90	0.007	−0.116	−1.011	0.005	0.000	0.011	0.016	0.945	0.960
100	0.007	0.333	−0.842	0.012	0.001	0.008	0.055	0.273	0.328
Active Total	1.000			1.380	1.000	1.000			

**Table 5:** Overview of raw points for GOLD stages in the correspondence analysis.

GOLD	Mass	Score in Dimension		Inertia	Contribution				
		1	2		of Point to Inertia of Dimension		of Dimension to Inertia of Point		
					1	2	1	2	Total
1	0.082	−1.687	0.913	0.266	0.273	0.149	0.756	0.119	0.875
2	0.247	−1.145	−0.140	0.305	0.378	0.011	0.912	0.007	0.920
3	0.380	0.435	−0.723	0.160	0.084	0.431	0.386	0.570	0.956
4	0.290	0.885	0.806	0.284	0.265	0.409	0.686	0.305	0.992
Active Total	1.000			1.016	1.000	1.000			

**Table 6:** Overview of column points for mMRC scale in the correspondence analysis.

mMRC	Mass	Score in Dimension		Inertia	Contribution				
		1	2		of Point to Inertia of Dimension		of Dimension to Inertia of Point		
					1	2	1	2	Total
1	0.183	−1.546	0.568	0.414	0.509	0.128	0.905	0.066	0.971
2	0.183	−0.794	−0.566	0.163	0.134	0.127	0.606	0.165	0.771
3	0.444	0.564	−0.481	0.180	0.165	0.223	0.674	0.263	0.937
4	0.190	0.932	1.124	0.259	0.192	0.521	0.548	0.427	0.975
Active Total	1.000			1.016	1.000	1.000			

**Table 7:** Overview of raw points for GOLD stages in the correspondence analysis.

GOLD	Mass	Score in Dimension		Inertia	Contribution				
		1	2		of Point to Inertia of Dimension		of Dimension to Inertia of Point		
					1	2	1	2	Total
1	0.102	0.499	−10.343	0.196	0.045	0.350	0.073	0.492	0.565
2	0.242	−0.956	0.706	0.210	0.393	0.230	0.590	0.301	0.892
3	0.372	0.831	0.452	0.189	0.458	0.145	0.766	0.212	0.977
4	0.284	−0.453	−0.713	0.166	0.104	0.275	0.198	0.458	0.656
Active Total	1.000			0.760	1.000	1.000			

**Table 8:** Overview of column points for FEV1/FVC values in the correspondence analysis.

FEV1FVC	Mass	Score in Dimension		Inertia	Contribution				
		1	2		of Point to Inertia of Dimension		of Dimension to Inertia of Point		
					1	2	1	2	Total
28	0.004	−0.806	−1.358	0.009	0.004	0.012	0.145	0.384	0.529
29	0.004	−1.701	1.346	0.011	0.018	0.012	0.519	0.304	0.823
30	0.007	0.041	−1.959	0.016	0.000	0.051	0.000	0.862	0.862
32	0.011	−1.403	0.445	0.013	0.037	0.004	0.901	0.085	0.985
34	0.028	−0.594	−1.508	0.052	0.018	0.122	0.107	0.646	0.754
35	0.011	−0.241	−1.758	0.017	0.001	0.062	0.020	0.980	1.000
36	0.018	−1.164	−0.277	0.016	0.042	0.003	0.821	0.043	0.864
37	0.011	0.718	0.122	0.006	0.010	0.000	0.494	0.013	0.508
38	0.004	1.480	0.862	0.006	0.014	0.005	0.729	0.231	0.960
39	0.014	−0.458	−0.127	0.004	0.005	0.000	0.386	0.028	0.413
40	0.004	1.480	0.862	0.006	0.014	0.005	0.729	0.231	0.960
41	0.007	−0.806	−1.358	0.018	0.008	0.025	0.145	0.384	0.529
42	0.021	0.470	0.002	0.003	0.008	0.000	0.788	0.000	0.788
43	0.011	−0.641	1.184	0.012	0.008	0.028	0.203	0.649	0.852
44	0.014	0.113	0.428	0.002	0.000	0.005	0.048	0.640	0.688
45	0.011	−0.342	0.283	0.002	0.002	0.002	0.443	0.283	0.726
46	0.011	0.718	0.122	0.006	0.010	0.000	0.494	0.013	0.508
47	0.021	−0.342	0.283	0.003	0.004	0.003	0.443	0.283	0.726
48	0.063	1.049	0.642	0.058	0.124	0.050	0.670	0.234	0.904
49	0.014	0.685	0.983	0.011	0.012	0.026	0.342	0.658	1.000
50	0.018	−0.707	0.167	0.006	0.016	0.001	0.847	0.044	0.891
51	0.014	1.332	0.007	0.016	0.044	0.000	0.885	0.000	0.885
52	0.014	−1.253	−0.006	0.013	0.039	0.000	0.968	0.000	0.968
53	0.021	0.619	−0.448	0.007	0.014	0.008	0.641	0.314	0.955
54	0.004	−1.701	1.346	0.011	0.018	0.012	0.519	0.304	0.823
55	0.025	−0.166	−0.123	0.001	0.001	0.001	0.350	0.178	0.528
56	0.025	0.245	0.683	0.007	0.003	0.022	0.117	0.853	0.970
57	0.014	0.761	−0.548	0.007	0.014	0.008	0.642	0.312	0.954
58	0.039	−0.589	0.476	0.012	0.024	0.017	0.619	0.378	0.997
59	0.035	−0.309	0.269	0.005	0.006	0.005	0.377	0.268	0.646
60	0.007	−1.253	−0.006	0.006	0.020	0.000	0.968	0.000	0.968
61	0.018	0.208	1.055	0.011	0.001	0.037	0.039	0.930	0.968
62	0.021	−0.491	0.734	0.009	0.009	0.022	0.316	0.659	0.975
63	0.042	−0.880	0.714	0.042	0.058	0.041	0.434	0.267	0.701
64	0.018	1.480	0.862	0.030	0.068	0.025	0.729	0.231	0.960
65	0.011	0.718	0.122	0.006	0.010	0.000	0.494	0.013	0.508
66	0.021	0.039	0.653	0.005	0.000	0.017	0.004	0.979	0.982
67	0.021	1.283	−0.278	0.027	0.062	0.003	0.718	0.032	0.750
68	0.018	−0.071	0.071	0.003	0.000	0.000	0.018	0.017	0.034
69	0.014	−0.035	−0.427	0.004	0.000	0.005	0.003	0.368	0.371
70	0.014	−0.458	−0.127	0.004	0.005	0.000	0.386	0.028	0.413
71	0.014	−0.035	−0.427	0.004	0.000	0.005	0.003	0.368	0.371
72	0.018	0.447	−0.710	0.007	0.006	0.017	0.291	0.686	0.977
74	0.007	1.480	0.862	0.012	0.027	0.010	0.729	0.231	0.960
75	0.021	−0.539	−0.857	0.020	0.011	0.029	0.174	0.409	0.583
76	0.014	−0.458	−0.127	0.004	0.005	0.000	0.386	0.028	0.413
77	0.011	0.420	1.023	0.007	0.003	0.021	0.151	0.840	0.992
78	0.011	0.521	−1.018	0.008	0.005	0.021	0.195	0.696	0.891
79	0.025	0.089	−0.895	0.014	0.000	0.038	0.008	0.740	0.747
80	0.021	0.521	−1.018	0.016	0.010	0.042	0.195	0.696	0.891
81	0.025	0.288	−0.192	0.002	0.004	0.002	0.707	0.293	1.000
82	0.018	0.447	−0.710	0.007	0.006	0.017	0.291	0.686	0.977
83	0.028	−0.246	−0.277	0.002	0.003	0.004	0.458	0.541	0.999
84	0.025	−0.863	0.118	0.011	0.033	0.001	0.910	0.016	0.926
85	0.004	1.480	0.862	0.006	0.014	0.005	0.729	0.231	0.960
86	0.018	−0.189	−0.613	0.004	0.001	0.013	0.088	0.863	0.951
87	0.011	0.223	−0.117	0.009	0.001	0.000	0.033	0.008	0.041
88	0.011	1.283	−0.278	0.014	0.031	0.002	0.718	0.032	0.750
89	0.007	−1.701	1.346	0.022	0.036	0.024	0.519	0.304	0.823
90	0.004	0.888	−2.559	0.031	0.005	0.044	0.050	0.389	0.440
91	0.004	0.888	−2.559	0.031	0.005	0.044	0.050	0.389	0.440
92	0.007	−1.253	−0.006	0.006	0.020	0.000	0.968	0.000	0.968
93	0.004	−0.806	−1.358	0.009	0.004	0.012	0.145	0.384	0.529
94	0.004	−1.701	1.346	0.011	0.018	0.012	0.519	0.304	0.823
Active Total	1.000			0.760	1.000	1.000			

**Table 9:** Overview of row points for GOLD categories in the correspondence analysis (symmetrical normalization).

WHO-5 Correspondence Analysis—Overview Row Points ^a^									
GOLD	Mass	Score in Dimension		Inertia	Contribution				
		1	2		of Point to Inertia of Dimension		of Dimension to Inertia of Point		
					1	2	1	2	Total
1	0.095	−0.048	−0.092	0.029	0.001	0.003	0.003	0.007	0.010
2	0.233	−0.871	0.558	0.085	0.477	0.269	0.764	0.228	0.992
3	0.389	−0.065	−0.614	0.042	0.004	0.545	0.014	0.939	0.953
4	0.282	0.823	0.418	0.085	0.518	0.183	0.836	0.157	0.994
Active Total	1.000			0.241	1.000	1.000			

a. Symmetrical normalization.

**Table 10:** Overview of column points for WHO-5 percentage scores in the correspondence analysis (symmetrical normalization).

WHO−5 Correspondence Analysis—Overview Column Points ^a^									
WHO 5%	Mass	Score in Dimension		Inertia	Contribution				
		1	2		of Point to Inertia of Dimension		of Dimension to Inertia of Point		
					1	2	1	2	Total
20	0.031	1.031	−0.122	0.013	0.088	0.002	0.959	0.010	0.969
24	0.053	−0.448	−0.213	0.006	0.029	0.009	0.685	0.112	0.797
26	0.023	−0.858	0.857	0.012	0.046	0.062	0.508	0.370	0.878
28	0.073	0.118	−0.381	0.003	0.003	0.039	0.114	0.873	0.987
32	0.011	−0.902	−0.830	0.007	0.025	0.029	0.486	0.300	0.785
36	0.042	−0.333	−0.397	0.008	0.013	0.025	0.204	0.211	0.415
38	0.027	−0.480	−1.382	0.016	0.017	0.189	0.139	0.840	0.979
40	0.160	0.480	−0.266	0.018	0.100	0.042	0.770	0.172	0.942
42	0.004	2.227	1.552	0.010	0.051	0.034	0.721	0.255	0.977
44	0.034	−0.363	0.237	0.002	0.012	0.007	0.729	0.226	0.955
46	0.011	1.426	0.274	0.010	0.063	0.003	0.875	0.024	0.899
48	0.080	0.420	−0.496	0.013	0.038	0.073	0.406	0.412	0.818
50	0.031	−0.092	0.788	0.008	0.001	0.070	0.012	0.638	0.649
52	0.084	0.009	0.674	0.010	0.000	0.141	0.000	1.000	1.000
55	0.027	0.238	0.882	0.007	0.004	0.077	0.078	0.784	0.862
56	0.046	0.284	0.566	0.005	0.010	0.054	0.257	0.740	0.997
60	0.111	−0.356	−0.152	0.008	0.038	0.009	0.658	0.087	0.745
64	0.015	1.626	0.594	0.018	0.109	0.020	0.849	0.082	0.931
68	0.073	−0.351	0.206	0.007	0.024	0.011	0.443	0.112	0.555
70	0.004	2.227	1.552	0.010	0.051	0.034	0.721	0.255	0.977
72	0.019	−0.131	−0.644	0.005	0.001	0.029	0.026	0.469	0.495
80	0.042	−1.562	0.488	0.045	0.277	0.037	0.836	0.059	0.896
Active Total	1.000			0.241	1.000	1.000			

a. Symmetrical normalization.

**Table 11:** Overview of row points for GOLD stages in the correspondence analysis (one-dimensional solution, symmetrical normalization).

MARS Correspondence Analysis—Overview Row Points ^a^						
GOLD	Mass	Score in Dimension	Inertia	Contribution		
		1		of Point to Inertia of Dimension	of Dimension to Inertia of Point	
				1	1	Total
1	0.102	−0.579	0.002	0.634	1.000	1.000
2	0.242	0.223	0.001	0.224	1.000	1.000
3	0.372	0.102	0.000	0.071	1.000	1.000
4	0.284	−0.116	0.000	0.070	1.000	1.000
Active Total	1.000		0.003	1.000		

a. Symmetrical normalization.

**Table 12:** Overview of column points for treatment adherence in the correspondence analysis (symmetrical normalization).

MARS Correspondence Analysis—Overview Column Points ^a^						
MARS	Mass	Score in Dimension	Inertia	Contribution		
		1		of Point to Inertia of Dimension	of Dimension to Inertia of Point	
				1	1	Total
non-adherent	0.284	−0.368	0.002	0.716	1.000	1.000
adherent	0.716	0.146	0.001	0.284	1.000	1.000
Active Total	1.000		0.003	1.000		

a. Symmetrical normalization.

## Data Availability

Data are contained within the article or Supplementary Material.

## Supplementary Materials

The following supporting information can be downloaded at: https://www.mdpi.com/article/10.3390/healthcare14040514/s1.
