# Supplementary material for: Psychological Well-Being and Treatment Adherence in COPD Patients: A Correspondence Analysis of WHO-5, MARS, and GOLD Severity
Source: Healthcare (Basel). 2026 Feb 18;14(4):514. doi: 10.3390/healthcare14040514 (PMC12940593; doi:10.3390/healthcare14040514)
Supplement: Supplementary file 1 [file healthcare-14-00514-s001.zip › healthcare-4119978-supplementary.pdf]

SUPPLEMENTARY MATERIAL 1

Table S1: WHO-5 well being index

Please respond to each item by marking <u>one box per row,</u> regarding how you felt in the last two weeks.		All of the time	Most of the time	More than half the time	Less than half the time	Some of the time	At no time
WHO 1	I have felt cheerful in good spirits.	☐ 5	☐ 4	☐ 3	☐ 2	☐ 1	☐ 0
WHO 2	I have felt calm and relaxed.	☐ 5	☐ 4	☐ 3	☐ 2	☐ 1	☐ 0
WHO 3	I have felt active and vigorous.	☐ 5	☐ 4	☐ 3	☐ 2	☐ 1	☐ 0
WHO 4	I woke up feeling fresh and rested.	☐ 5	☐ 4	☐ 3	☐ 2	☐ 1	☐ 0
WHO 5	My daily life has been filled with things that interest me.	☐ 5	☐ 4	☐ 3	☐ 2	☐ 1	☐ 0

Table S2: MARS questionnaire

	Question	Answer
1	Do you ever forget to take your medication?	Yes / No
2	Are you careless at times about taking your medication?	Yes / No
3	When you feel better, do you sometimes stop taking your medication?	Yes / No
4	Sometimes if you feel worse when you take the medication, do you stop taking it?	Yes / No
5	I take my medication only when I am sick	Yes / No
6	It is unnatural for my mind and body to be controlled by medication	Yes / No
7	My thoughts are clearer on medication	Yes / No
8	By staying on medication, I can prevent getting sick.	Yes / No
9	I feel weird, like a 'zombie' on medication	Yes / No
10	Medication makes me feel tired and sluggish	Yes / No

SUPPLEMENTARY MATERIAL 2

Table S1: Distribution by Age

Age

	N	%
34	1	0.4%
38	1	0.4%
39	1	0.4%
40	1	0.4%
45	1	0.4%
47	3	1.1%
48	3	1.1%
49	4	1.4%
50	3	1.1%
51	1	0.4%
52	8	2.8%
53	4	1.4%
54	4	1.4%
55	5	1.8%
56	12	4.2%
57	7	2.5%
58	9	3.2%
59	8	2.8%
60	6	2.1%
61	9	3.2%
62	3	1.1%
63	5	1.8%
64	12	4.2%
65	11	3.9%
66	12	4.2%
67	13	4.6%
68	18	6.3%
69	13	4.6%
70	13	4.6%
71	13	4.6%
72	10	3.5%
73	14	4.9%
74	11	3.9%
75	9	3.2%
76	7	2.5%
77	4	1.4%

78	8	2.8%
80	1	0.4%
81	8	2.8%
82	3	1.1%
a83	1	0.4%
84	2	0.7%
86	1	0.4%
89	1	0.4%
96	1	0.4%

Table S2: Distribution by Sex

Sex

	N	%
male	217	76.2%
female	68	23.8%

Table S3: Distribution by Obesity

Obesity

	N	%
no	175	61.4%
yes	110	38.6%

Figure S1: Histogram of Age

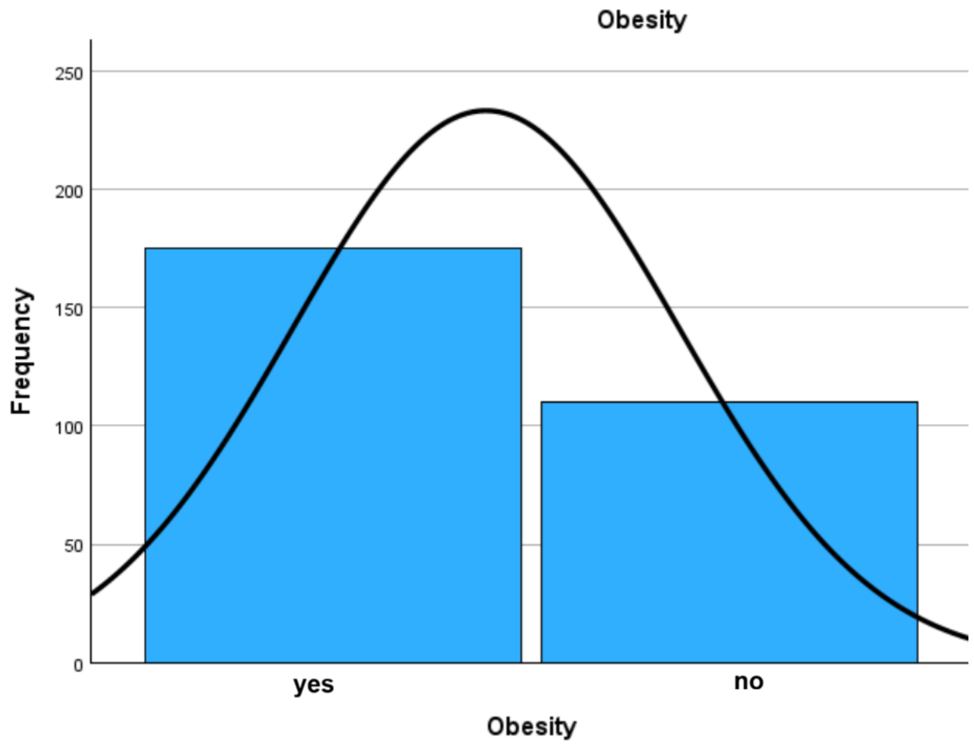

COPD

	N	%
yes	285	100.0%

Figure S2: Histogram of COPD

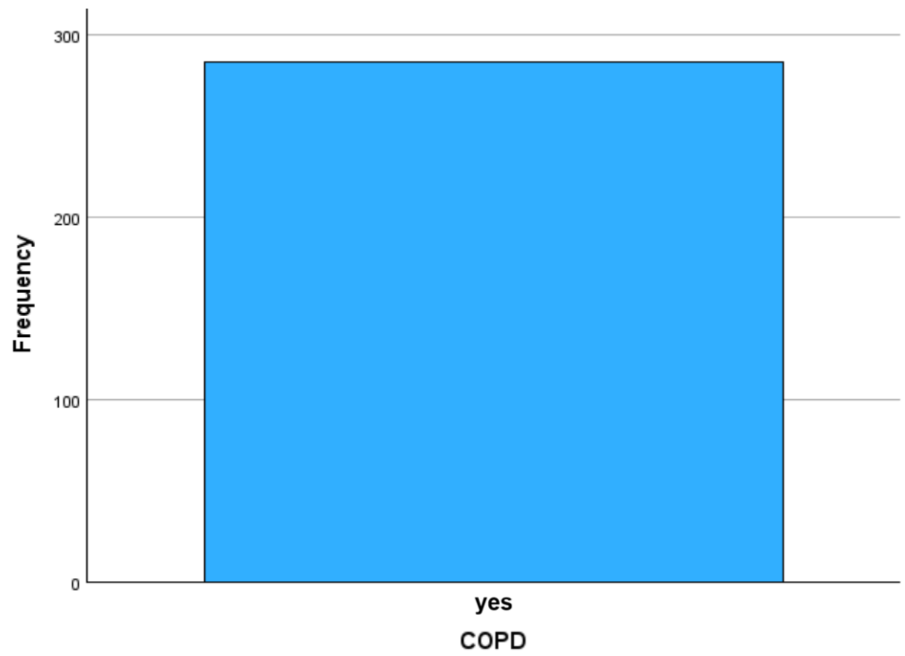

Table S4: Distribution by Other COPD

Other COPD

	N	%
no	271	95.1%
yes	14	4.9%

Figure S3: Histogram of Other COPD

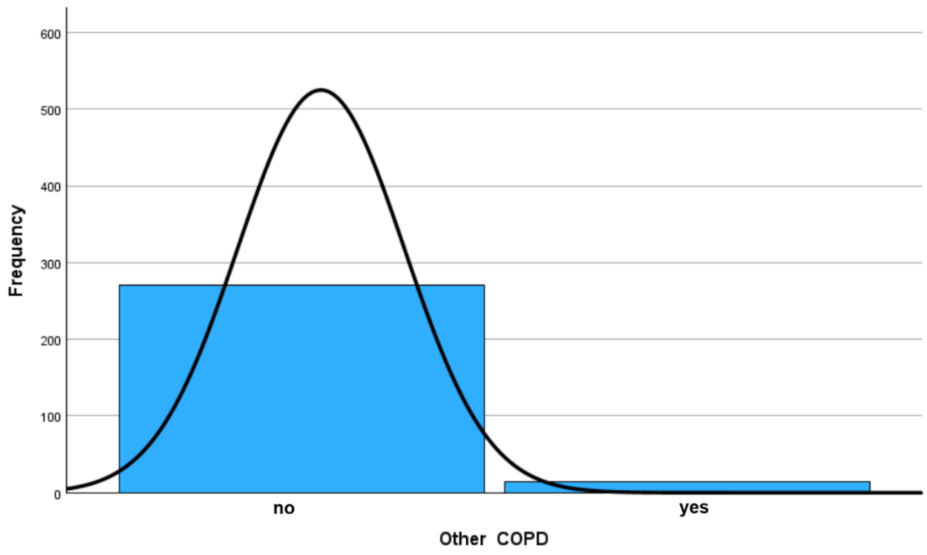

Table S5: Distribution by Respiratory failure

Respiratory failure

	N	%
no	110	38.6%
yes	175	61.4%

Figure S4: Histogram of Respiratory failure

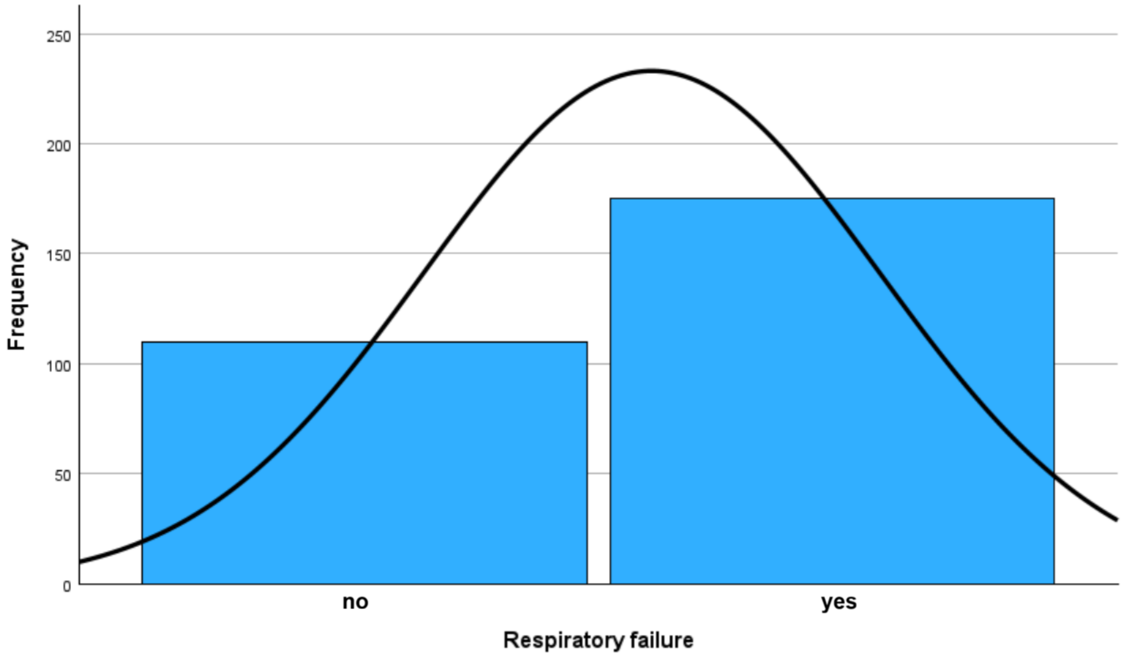

Table S6: Distribution by Other pulmonary diseases

Other pulmonary diseases

	N	%
no	187	65.6%
yes	98	34.4%

Figure S5: Histogram of Other pulmonary diseases

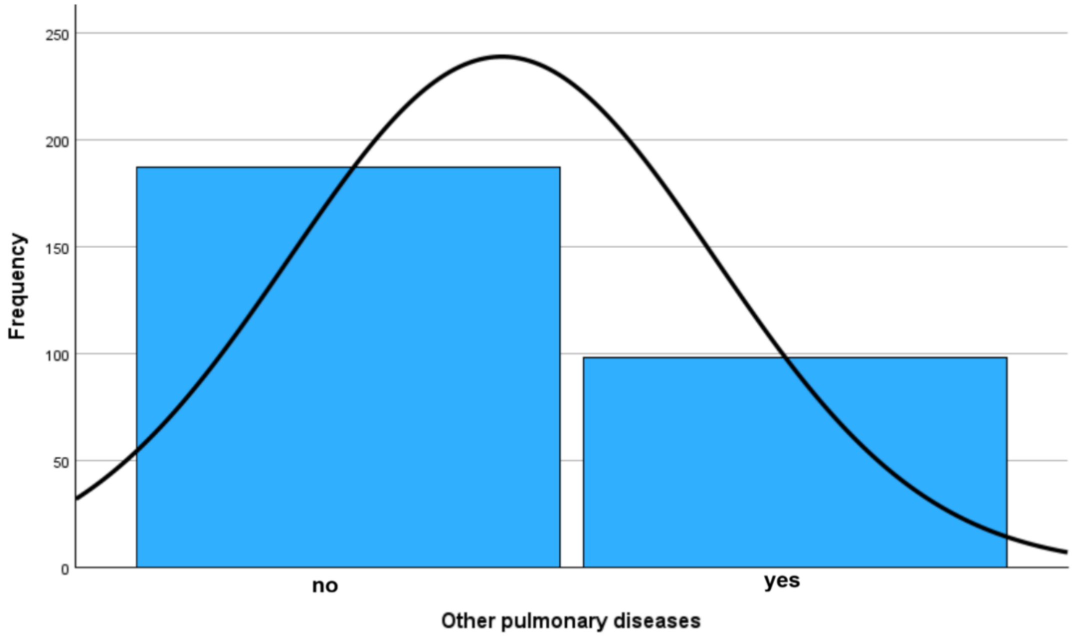

Table S7: Distribution by Pulmonary embolism

Pulmonary embolism

	N	%
no	236	82.8%
yes	49	17.2%

Figure S6: Histogram of Pulmonary embolism

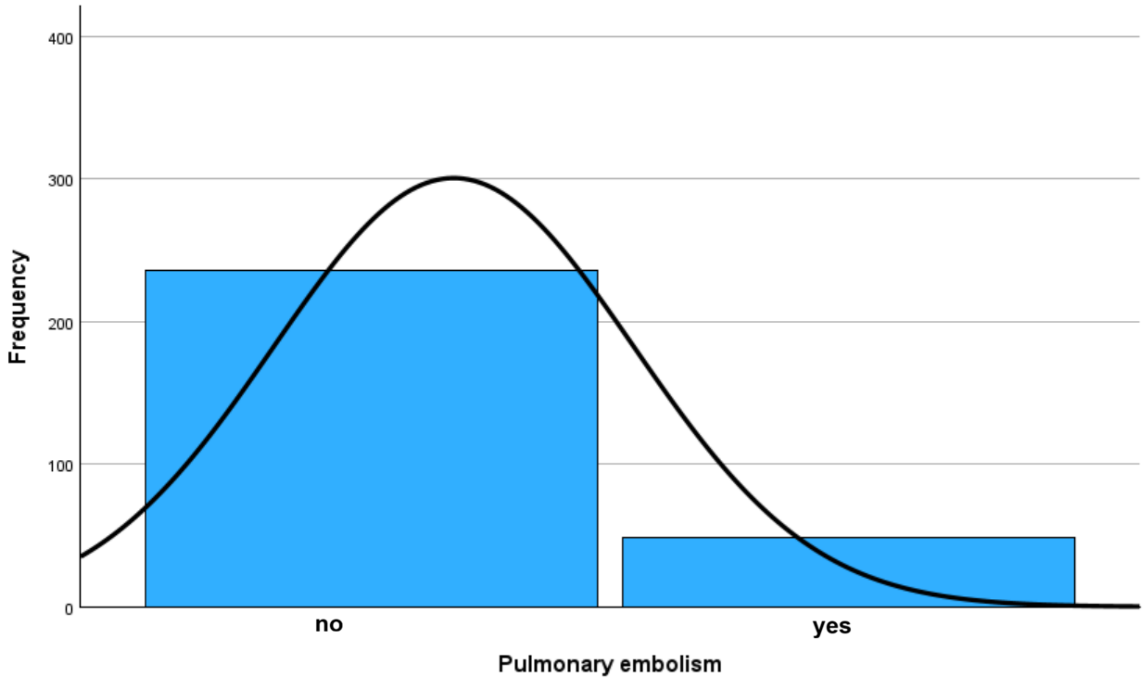

Table S8: Distribution by Heart disease

Heart disease

	N	%
no	211	74.0%
yes	74	26.0%

Figure S7: Histogram of Heart disease

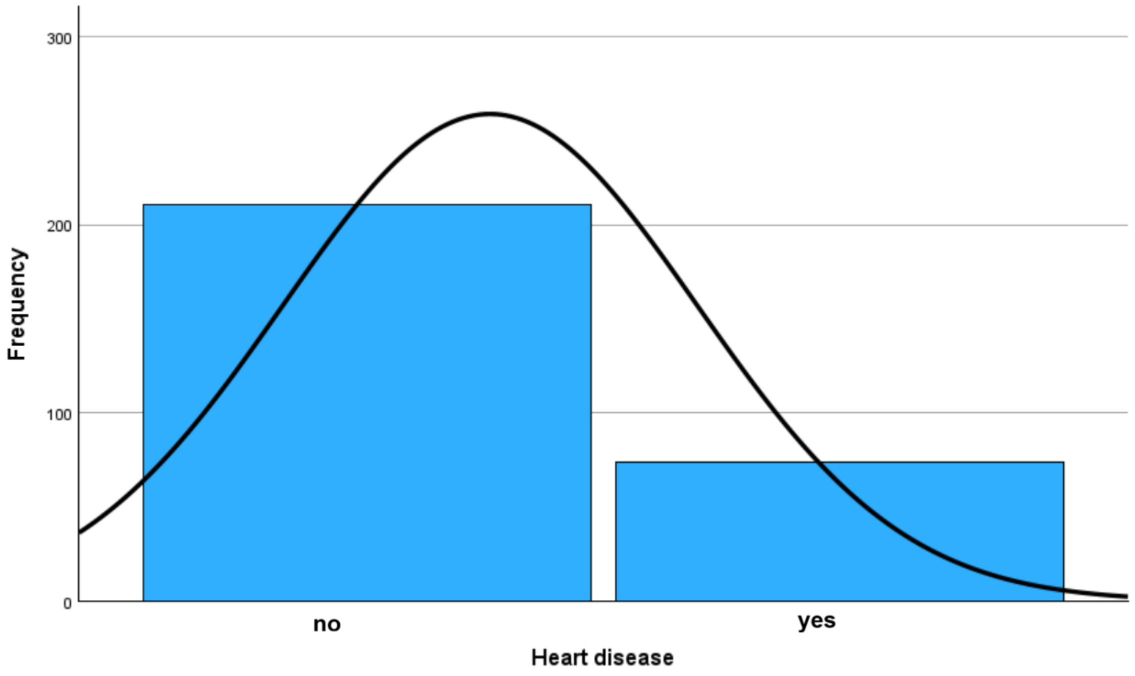

Table S9: Distribution by Cardiac failure

Cardiac failure

	N	%
no	172	60.4%
yes	113	39.6%

Figure S8: Histogram of Cardiac failurie

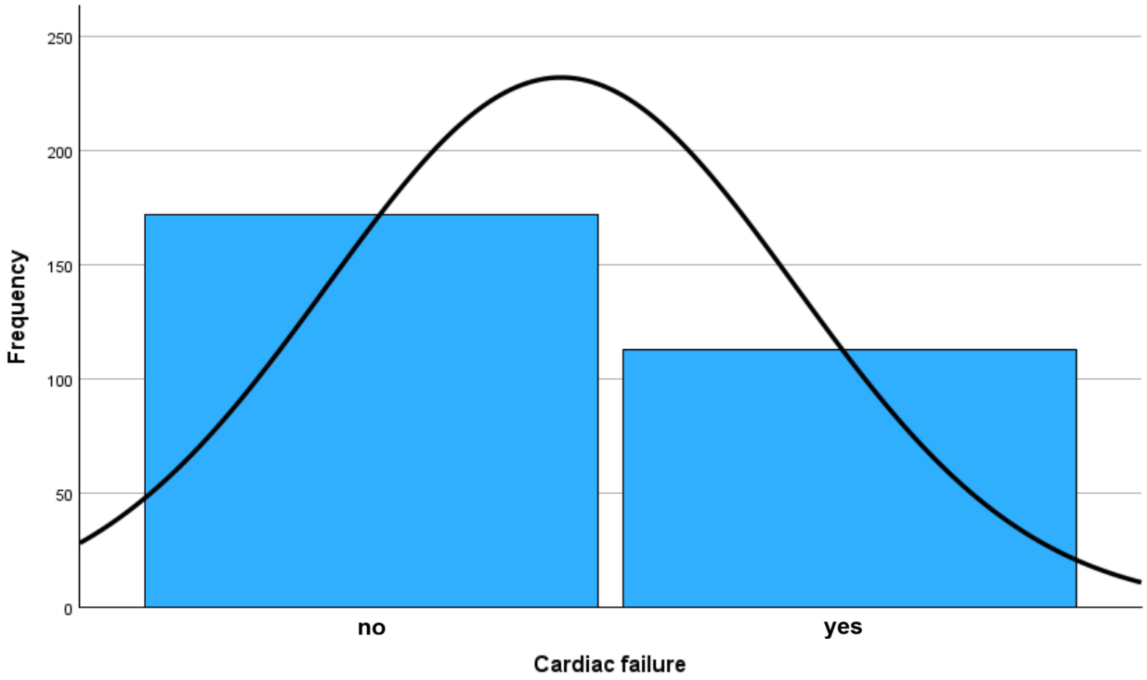

Table S10: Distribution by Effort related fatigue

Effort related fatigue

	N	%
no	102	35.8%
yes	183	64.2%

Figure S9: Histogram of Effort related fatigue

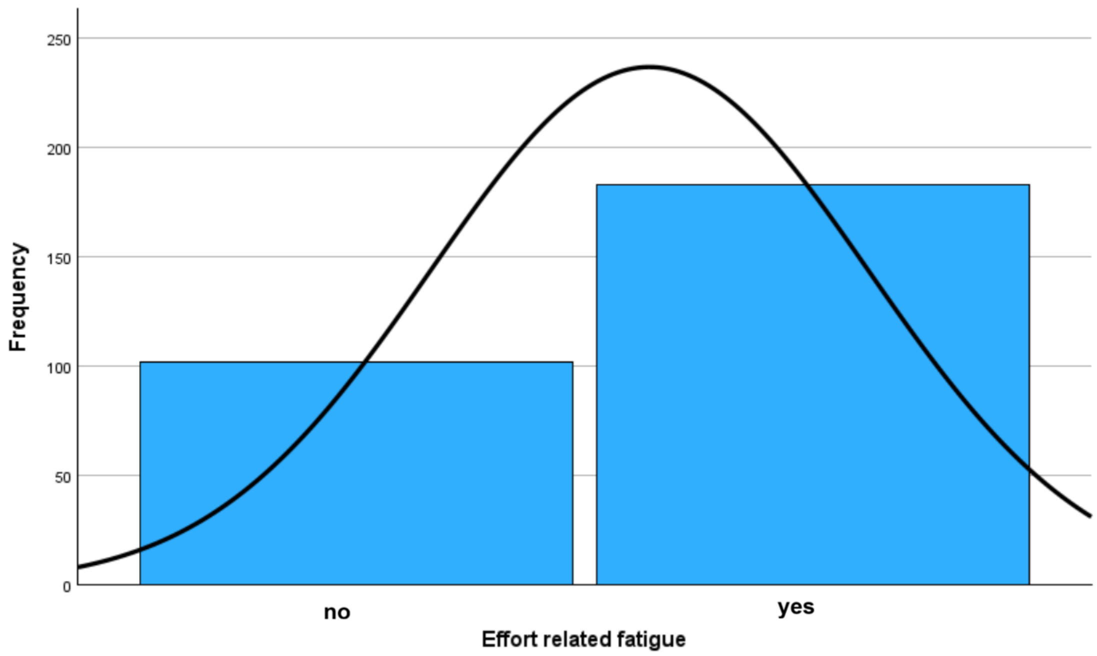

Table S11: Distribution by Diabetes

Diabetes

	N	%
no	143	50.2%
yes	142	49.8%

Figure S10: Histogram of Diabetes

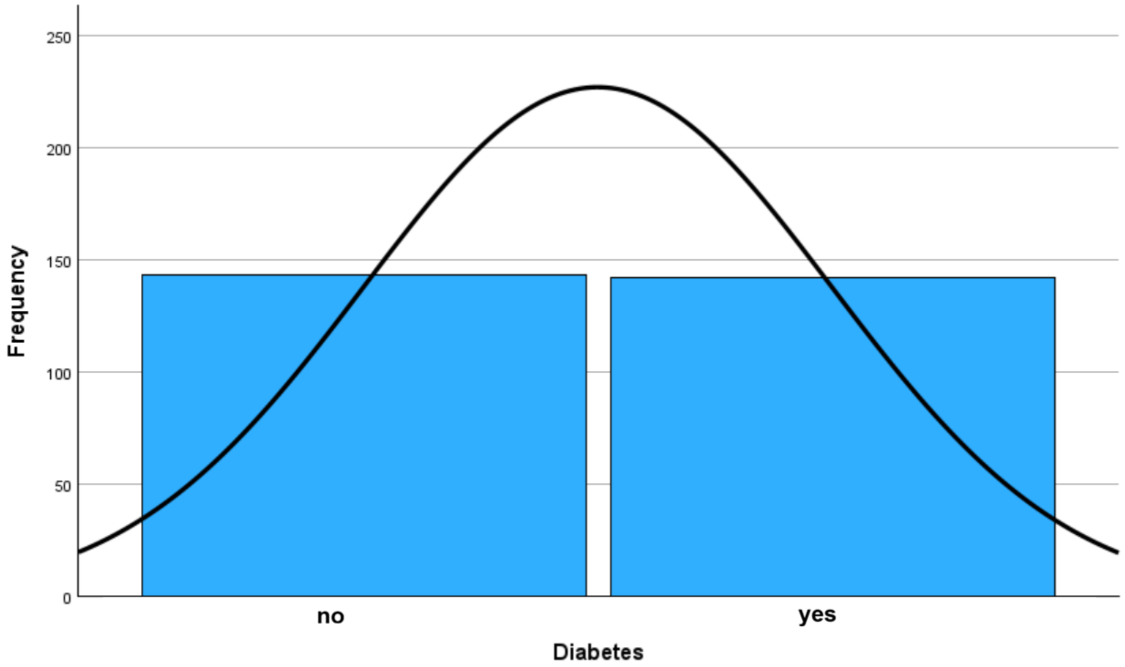

Table S12: Distribution by Atrial fibrillation and/or flutter

Atrial fibrillation and/or flutter

	N	%
no	242	84.9%
yes	43	15.1%

Figure S11: Histogram of Atrial fibrillation and/or flutter

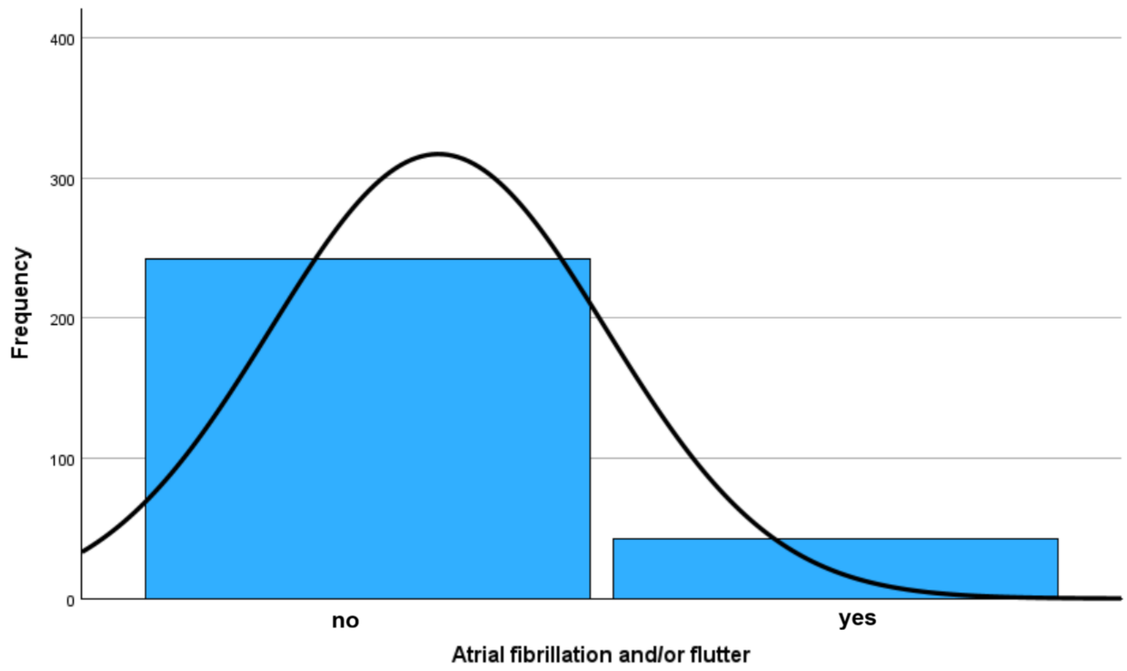

Table S13: Case summaries

Case Summaries[a]

		Vesicular breath sounds	Crackles	Cough	Sputum	Fever	Chills	O2 therapy	CAT	mMRC	MARS	WHO_5
GOLD1	1	yes	no	no	no	no	no	no	10	0	adherent	84%
	2	no	yes	yes	no	no	no	no	20	2	non-adherent	86%
	3	no	no	yes	yes	no	no	no	25	2	adherent	40%
	4	yes	yes	yes	no	no	no	no	26	2	adherent	40%
	5	yes	no	no	no	no	no	no	22	1	adherent	60%
	6	no	no	no	no	no	no	no	28	1	adherent	44%
	7	no	no	no	no	no	no	no	28	1	adherent	24%
	8	no	no	no	no	no	no	yes	30	1	non-adherent	55%
	9	no	no	no	no	no	no	no	27	1	adherent	26%

	10	no	no	no	no	no	no	yes	27	1	adherent	28%
	11	no	no	no	no	no	no	no	18	1	non-adherent	68%
	12	yes	yes	no	no	no	no	no	28	0	adherent	24%
	13	yes	no	no	no	no	no	yes	31	1	non-adherent	52%
	14	no	yes	yes	no	no	no	yes	23	0	adherent	60%
	15	no	yes	no	no	no	yes	yes	26	1	non-adherent	38%
	16	no	no	no	no	no	yes	no	18	1	non-adherent	68%
	17	no	no	no	no	no	no	no	20	1	non-adherent	68%
	18	no	yes	no	no	no	yes	no	10	1	adherent	88%
	19	yes	no	no	no	no	no	no	7	0	adherent	92%
	20	yes	yes	no	no	no	no	yes	22	1	adherent	40%
	21	yes	yes	no	no	no	no	no	31	1	adherent	48%
	22	yes	no	no	yes	yes	no	no	33	1	adherent	20%
	23	yes	no	no	no	no	no	yes	28	0	non-adherent	40%
	24	yes	yes	yes	no	no	no	yes	25	1	adherent	40%
	25	yes	no	no	no	no	no	yes	27	1	adherent	28%
	26	no	no	no	no	no	yes	no	27	1	adherent	60%
	27	no	no	no	no	no	no	yes	20	0	adherent	56%
	28	yes	yes	no	yes	no	no	yes	17	1	non-adherent	52%
	29	yes	yes	no	no	no	yes	no	28	1	non-adherent	60%
	TotalN	29	29	29	29	29	29	29	29	29	29	29
2	1	no	no	yes	yes	no	no	no	30	3	non-adherent	55%
	2	no	no	yes	yes	no	no	no	30	3	non-adherent	52%
	3	no	yes	yes	yes	no	no	no	31	3	non-adherent	52%
	4	no	yes	yes	yes	no	no	no	33	3	non-adherent	50%
	5	no	yes	yes	yes	no	no	no	34	3	non-adherent	48%
	6	no	no	yes	yes	no	no	no	25	3	adherent	40%
	7	no	no	yes	yes	no	no	no	7	1	adherent	92%

8	no	yes	yes	yes	no	no	no	7	1	adherent	92%
9	no	yes	yes	no	no	no	no	7	1	adherent	92%
10	yes	yes	yes	no	no	no	no	7	1	adherent	92%
11	no	no	yes	no	yes	no	no	15	2	non-adherent	80%
12	no	no	yes	yes	no	no	no	10	1	adherent	80%
13	yes	yes	yes	yes	no	no	no	18	2	non-adherent	28%
14	no	yes	yes	yes	no	no	yes	20	2	adherent	44%
15	no	yes	yes	no	no	no	yes	22	2	adherent	44%
16	yes	yes	yes	yes	no	no	no	20	2	adherent	80%
17	no	yes	yes	no	no	no	yes	28	3	non-adherent	60%
18	yes	yes	yes	yes	no	no	no	10	1	adherent	60%
19	no	no	yes	yes	no	no	no	17	2	adherent	56%
20	yes	yes	yes	yes	no	no	no	17	2	adherent	56%
21	yes	yes	yes	no	no	no	no	26	2	adherent	44%
22	no	yes	yes	yes	no	no	no	13	1	adherent	68%
23	no	no	yes	yes	no	no	yes	16	1	adherent	68%
24	yes	yes	yes	no	yes	no	no	8	1	adherent	80%
25	no	no	no	no	no	no	no	8	1	adherent	80%
26	no	yes	no	no	no	no	yes	10	1	adherent	80%
27	no	yes	yes	yes	no	no	yes	10	1	adherent	80%
28	no	yes	yes	no	no	no	no	23	2	non-adherent	60%
29	no	no	yes	yes	no	no	no	18	2	non-adherent	52%
30	no	yes	yes	yes	no	no	no	10	2	adherent	84%
31	no	no	no	no	no	no	no	28	1	adherent	24%
32	no	no	no	no	no	no	no	23	2	adherent	40%
33	no	no	no	no	no	no	yes	26	2	non-adherent	38%
34	no	no	no	no	no	no	no	26	1	non-adherent	36%
35	no	no	no	no	no	no	no	27	1	adherent	28%

36	no	no	no	no	no	no	yes	10	2	adherent	88%
37	no	no	no	no	no	no	no	25	1	non-adherent	68%
38	no	no	no	no	no	no	no	28	2	adherent	24%
39	no	no	no	no	no	no	no	28	2	adherent	24%
40	yes	yes	no	no	no	no	yes	23	1	adherent	60%
41	no	no	no	no	no	no	no	25	1	adherent	50%
42	no	no	no	no	no	no	no	27	2	adherent	26%
43	yes	no	no	no	no	yes	no	21	2	adherent	60%
44	no	no	no	no	no	no	no	7	1	adherent	92%
45	no	no	no	no	no	no	yes	7	1	adherent	92%
46	no	yes	yes	no	no	no	yes	15	2	adherent	32%
47	no	yes	no	no	no	yes	yes	28	2	adherent	52%
48	no	no	no	no	no	no	yes	30	2	non-adherent	60%
49	no	no	no	no	no	no	no	34	1	adherent	72%
50	no	no	no	no	no	no	no	25	2	adherent	52%
51	no	yes	no	no	no	no	yes	25	2	adherent	60%
52	no	no	no	no	no	no	no	24	1	non-adherent	36%
53	no	yes	no	yes	no	yes	yes	26	2	adherent	26%
54	no	no	no	no	no	no	no	27	1	non-adherent	68%
55	no	yes	yes	no	no	no	yes	10	2	adherent	24%
56	no	yes	yes	no	no	no	yes	29	1	adherent	55%
57	yes	yes	yes	no	no	no	no	30	1	adherent	48%
58	yes	yes	no	no	no	no	yes	33	2	adherent	28%
59	yes	no	no	no	no	no	yes	34	1	adherent	40%
60	yes	yes	no	no	no	no	no	25	1	adherent	60%
61	no	no	no	no	no	no	no	27	2	adherent	68%
62	no	yes	no	no	no	yes	yes	21	1	adherent	56%
63	yes	yes	no	no	no	yes	no	10	1	adherent	52%

	64	yes	yes	yes	yes	no	no	no	34	2	non-adherent	52%
	65	no	yes	no	no	no	no	no	28	2	adherent	50%
	66	yes	no	no	no	no	no	no	25	1	non-adherent	40%
	67	yes	yes	no	no	no	no	yes	23	2	adherent	36%
	68	yes	no	no	yes	no	yes	yes	20	2	adherent	26%
	69	yes	yes	yes	no	no	yes	yes	26	2	adherent	68%
	TotalN	69	69	69	69	69	69	69	69	69	69	69
3	1	yes	yes	yes	yes	yes	no	no	22	3	adherent	60%
	2	yes	yes	yes	yes	no	no	no	28	3	adherent	24%
	3	yes	yes	yes	yes	no	no	no	28	3	adherent	24%
	4	yes	yes	yes	yes	no	no	no	28	3	adherent	24%
	5	yes	yes	yes	yes	no	no	no	28	4	adherent	24%
	6	no	no	yes	yes	no	no	no	17	3	adherent	72%
	7	no	no	yes	yes	no	no	no	28	3	non-adherent	28%
	8	no	no	yes	yes	no	no	no	25	3	adherent	40%
	9	no	no	yes	yes	no	no	yes	22	2	adherent	52%
	10	no	yes	no	no	no	no	yes	23	3	adherent	60%
	11	no	no	no	no	no	no	yes	23	3	adherent	60%
	12	no	no	no	no	no	no	no	20	2	adherent	48%
	13	yes	yes	yes	yes	no	no	yes	26	4	non-adherent	38%
	14	yes	yes	yes	yes	no	no	yes	26	4	non-adherent	36%
	15	no	yes	yes	no	no	no	yes	21	3	adherent	60%
	16	no	yes	yes	no	no	no	no	18	2	non-adherent	68%
	17	no	yes	yes	no	no	no	no	10	1	adherent	88%
	18	yes	yes	yes	no	no	no	no	29	4	adherent	56%
	19	no	no	yes	no	no	no	no	15	3	adherent	32%
	20	no	no	yes	yes	no	no	no	22	3	adherent	40%
	21	no	yes	yes	yes	no	no	yes	24	4	adherent	48%

22	no	no	no	no	no	no	no	22	3	adherent	48%
23	yes	yes	yes	yes	no	no	yes	25	3	non-adherent	80%
24	yes	yes	yes	yes	no	no	yes	21	3	adherent	40%
25	no	no	yes	yes	no	no	yes	22	3	adherent	40%
26	no	no	yes	yes	no	no	yes	20	3	adherent	40%
27	yes	yes	yes	yes	no	no	yes	22	3	non-adherent	40%
28	yes	yes	yes	no	no	no	yes	21	3	non-adherent	40%
29	no	no	yes	yes	no	no	yes	35	4	adherent	36%
30	no	no	yes	yes	no	no	yes	35	4	adherent	80%
31	no	no	yes	yes	no	no	yes	20	3	adherent	48%
32	no	yes	yes	yes	no	no	yes	24	3	adherent	48%
33	no	yes	yes	no	no	no	no	26	3	adherent	28%
34	yes	yes	yes	yes	yes	yes	no	37	4	adherent	36%
35	no	yes	yes	no	no	no	no	25	4	adherent	40%
36	no	yes	yes	no	no	no	no	22	4	adherent	40%
37	no	no	no	no	no	no	yes	22	3	adherent	48%
38	no	yes	yes	yes	no	no	no	20	2	adherent	44%
39	no	yes	no	no	no	no	no	20	2	adherent	44%
40	no	yes	yes	yes	no	no	no	21	3	adherent	52%
41	no	yes	yes	yes	no	no	no	16	3	adherent	68%
42	no	yes	yes	yes	no	no	no	22	3	non-adherent	60%
43	no	no	yes	yes	no	no	no	21	3	adherent	64%
44	no	no	yes	yes	no	no	no	22	2	adherent	44%
45	no	no	yes	yes	no	no	yes	15	2	non-adherent	52%
46	yes	no	no	no	no	no	no	31	2	non-adherent	40%
47	yes	no	no	no	no	no	no	33	2	non-adherent	40%
48	no	no	no	no	no	no	yes	34	2	non-adherent	52%
49	yes	yes	yes	no	no	no	yes	34	3	non-adherent	60%

50	no	no	no	no	no	no	no	23	3	adherent	38%
51	no	no	no	no	no	no	no	23	2	adherent	36%
52	no	yes	no	no	no	yes	yes	25	3	adherent	50%
53	yes	yes	no	no	no	no	yes	20	3	adherent	48%
54	no	no	no	no	no	no	no	24	3	adherent	40%
55	yes	no	no	no	no	no	no	7	3	adherent	92%
56	no	no	no	no	no	no	no	15	3	adherent	32%
57	no	no	no	no	no	no	yes	23	3	non-adherent	20%
58	no	no	no	no	no	no	no	25	3	non-adherent	46%
59	no	no	no	no	no	no	no	22	3	adherent	40%
60	no	yes	no	no	no	no	yes	24	2	adherent	48%
61	no	no	no	no	no	no	no	15	3	non-adherent	80%
62	no	no	no	no	no	no	yes	10	2	adherent	80%
63	no	no	no	no	no	no	yes	22	3	non-adherent	68%
64	no	no	no	no	no	no	no	29	3	adherent	56%
65	yes	yes	yes	no	no	no	yes	30	3	adherent	92%
66	no	no	no	no	no	no	no	35	3	adherent	40%
67	no	no	no	no	no	yes	no	28	3	adherent	24%
68	no	no	no	no	no	no	yes	33	3	non-adherent	60%
69	yes	yes	no	no	no	no	no	34	3	non-adherent	48%
70	no	no	no	no	no	no	no	34	3	non-adherent	20%
71	no	no	no	no	no	no	yes	17	3	adherent	72%
72	yes	no	no	no	no	yes	yes	28	3	non-adherent	28%
73	no	no	no	no	no	no	no	25	3	adherent	40%
74	no	no	no	no	no	no	yes	25	3	adherent	40%
75	yes	yes	no	no	no	no	yes	20	3	adherent	48%
76	no	yes	no	no	no	yes	yes	26	3	non-adherent	36%
77	no	no	no	no	no	yes	yes	27	3	adherent	28%

78	no	no	no	no	no	no	yes	27	2	adherent	28%
79	yes	no	no	no	no	no	no	29	3	adherent	56%
80	no	no	no	no	no	no	no	28	3	adherent	24%
81	yes	yes	yes	no	no	no	no	22	3	adherent	60%
82	yes	yes	yes	no	no	no	no	23	3	non-adherent	38%
83	no	yes	yes	no	no	no	yes	26	3	adherent	28%
84	no	no	no	no	no	no	no	21	3	non-adherent	68%
85	no	no	no	no	no	yes	yes	30	3	adherent	20%
86	no	yes	no	no	no	yes	yes	31	3	adherent	72%
87	no	no	no	no	no	no	yes	25	3	adherent	50%
88	yes	no	no	no	no	no	no	22	3	adherent	48%
89	no	yes	no	yes	no	no	yes	25	3	non-adherent	38%
90	no	no	no	no	no	no	no	20	3	adherent	36%
91	yes	yes	no	no	no	no	yes	24	3	adherent	26%
92	yes	yes	no	no	no	no	yes	26	2	adherent	28%
93	no	no	no	no	no	no	no	26	3	non-adherent	60%
94	yes	no	no	no	no	no	yes	27	3	non-adherent	68%
95	no	no	no	no	no	no	no	27	2	adherent	88%
96	no	no	no	no	no	no	yes	20	3	adherent	55%
97	yes	yes	yes	yes	no	no	yes	29	3	adherent	52%
98	no	no	no	no	no	no	no	30	3	adherent	28%
99	no	no	no	no	no	yes	yes	31	3	adherent	40%
100	yes	no	no	no	no	no	yes	33	3	non-adherent	40%
101	yes	no	no	no	no	no	no	34	3	adherent	60%
102	yes	yes	yes	no	no	no	yes	17	3	adherent	60%
103	no	yes	no	no	no	yes	no	23	3	adherent	38%
104	yes	yes	no	no	no	no	no	25	3	adherent	28%
105	yes	yes	yes	no	no	yes	yes	24	3	adherent	60%

	106	yes	yes	no	no	no	no	yes	26	3	non-adherent	68%
	TotalN	106	106	106	106	106	106	106	106	106	106	106
4	1	no	yes	yes	yes	yes	no	yes	34	4	non-adherent	20%
	2	yes	yes	yes	yes	no	no	yes	25	4	adherent	50%
	3	no	yes	no	no	no	no	no	23	3	adherent	40%
	4	no	yes	yes	no	no	no	no	24	3	adherent	40%
	5	no	yes	yes	yes	no	yes	yes	27	4	adherent	28%
	6	no	no	yes	yes	no	yes	yes	27	4	adherent	26%
	7	yes	yes	yes	yes	no	no	yes	27	4	adherent	28%
	8	yes	yes	yes	yes	no	no	no	20	3	non-adherent	68%
	9	no	no	no	no	no	no	no	23	3	non-adherent	20%
	10	no	yes	no	no	no	no	yes	25	4	non-adherent	46%
	11	yes	yes	yes	yes	no	no	no	22	3	non-adherent	84%
	12	no	no	no	no	no	no	yes	29	3	adherent	48%
	13	no	no	no	no	no	no	yes	29	3	adherent	44%
	14	no	yes	yes	yes	no	no	yes	30	4	adherent	42%
	15	no	no	yes	yes	no	no	yes	35	4	adherent	40%
	16	no	no	yes	yes	no	no	yes	35	4	adherent	68%
	17	no	yes	yes	yes	no	no	yes	26	3	adherent	48%
	18	no	no	yes	no	no	no	yes	20	3	adherent	64%
	19	no	no	yes	no	no	no	yes	20	3	adherent	64%
	20	no	no	yes	no	no	no	yes	20	3	adherent	56%
	21	no	no	yes	yes	no	no	yes	18	3	adherent	56%
	22	no	yes	yes	no	no	no	yes	24	4	adherent	56%
	23	no	yes	yes	yes	no	no	yes	24	4	adherent	56%
	24	no	yes	yes	yes	no	no	no	20	4	adherent	52%
	25	no	yes	yes	yes	no	no	no	24	4	adherent	52%
	26	no	yes	yes	yes	no	no	yes	22	4	non-adherent	44%

27	no	no	yes	yes	no	no	no	21	3	adherent	68%
28	no	no	no	no	no	no	no	22	3	adherent	40%
29	no	no	no	no	no	no	no	22	3	adherent	40%
30	yes	yes	no	no	no	no	yes	28	4	adherent	36%
31	yes	yes	yes	yes	no	no	yes	28	4	adherent	36%
32	no	yes	yes	yes	no	no	no	24	3	non-adherent	70%
33	no	no	yes	yes	no	no	no	13	3	non-adherent	60%
34	no	no	yes	yes	no	no	yes	20	3	non-adherent	64%
35	no	no	no	no	no	no	no	28	4	adherent	24%
36	yes	yes	no	no	no	no	no	30	3	non-adherent	52%
37	no	no	no	no	no	no	no	17	3	adherent	60%
38	no	no	no	no	no	no	no	28	4	non-adherent	50%
39	no	no	no	no	no	no	yes	25	3	adherent	48%
40	no	no	no	no	no	no	no	25	3	adherent	40%
41	no	no	no	no	no	no	no	22	3	adherent	40%
42	yes	yes	yes	no	no	no	yes	21	4	adherent	60%
43	no	yes	yes	yes	yes	no	yes	20	3	non-adherent	68%
44	yes	yes	yes	no	no	no	no	29	3	adherent	56%
45	no	no	no	no	no	no	no	7	3	adherent	92%
46	no	no	no	no	no	no	yes	7	4	adherent	92%
47	yes	yes	no	no	no	no	yes	7	4	adherent	92%
48	no	no	no	no	no	no	no	22	3	adherent	48%
49	no	no	no	no	no	no	yes	29	4	adherent	88%
50	no	yes	yes	no	no	no	no	22	4	adherent	55%
51	yes	yes	no	yes	no	no	no	30	4	non-adherent	55%
52	yes	no	no	no	no	yes	yes	30	4	non-adherent	52%
53	no	yes	no	yes	yes	no	no	22	3	adherent	52%
54	no	no	no	no	no	no	yes	23	4	adherent	40%

55	no	yes	no	no	no	yes	yes	24	4	adherent	40%
56	no	no	no	no	no	no	no	7	4	adherent	92%
57	yes	no	no	yes	no	no	yes	23	4	non-adherent	20%
58	no	no	no	no	no	no	yes	25	4	non-adherent	46%
59	no	no	no	no	no	no	no	28	3	adherent	55%
60	no	no	no	no	no	no	no	30	3	non-adherent	52%
61	yes	yes	no	no	no	no	yes	34	3	adherent	28%
62	no	no	no	no	no	no	no	17	4	non-adherent	40%
63	yes	no	no	no	no	no	no	23	4	adherent	50%
64	yes	no	no	no	no	no	no	23	4	adherent	48%
65	yes	no	no	no	no	no	yes	20	3	adherent	40%
66	yes	no	no	no	no	yes	yes	18	4	adherent	88%
67	no	no	no	no	no	no	no	28	3	non-adherent	52%
68	no	no	no	no	no	no	no	28	4	non-adherent	52%
69	no	yes	no	no	no	no	yes	28	4	adherent	60%
70	yes	yes	no	yes	no	no	yes	34	3	adherent	40%
71	no	no	no	no	no	no	no	23	4	adherent	40%
72	yes	no	no	no	no	yes	yes	23	4	non-adherent	40%
73	no	no	no	no	no	no	yes	23	3	adherent	28%
74	no	no	no	no	no	yes	yes	18	4	adherent	24%
75	no	yes	no	no	yes	yes	yes	28	3	non-adherent	60%
76	no	no	no	no	no	no	yes	28	4	non-adherent	48%
77	no	yes	yes	no	no	no	yes	28	4	adherent	20%
78	yes	yes	no	no	no	no	no	30	3	adherent	72%
79	no	no	no	no	no	no	no	25	3	adherent	48%
80	yes	yes	no	no	no	yes	yes	22	4	non-adherent	40%
81	no	yes	no	yes	no	no	no	23	4	adherent	28%
TotalN	81	81	81	81	81	81	81	81	81	81	81

TotalN	285	285	285	285	285	285	285	285	285	285	285

a. Limited to first 500 cases.

Case Summaries[a]

		Vesicular breath sounds	Crackles	Cough	Sputum	Fever	Chills	O2 therapy	CAT	mMRC	MARS	WHO_5
GOLD1	1	yes	no	no	no	no	no	no	10	0	adherent	84%
	2	no	yes	yes	no	no	no	no	20	2	non-adherent	86%
	3	no	no	yes	yes	no	no	no	25	2	adherent	40%
	4	yes	yes	yes	no	no	no	no	26	2	adherent	40%
	5	yes	no	no	no	no	no	no	22	1	adherent	60%
	6	no	no	no	no	no	no	no	28	1	adherent	44%
	7	no	no	no	no	no	no	no	28	1	adherent	24%
	8	no	no	no	no	no	no	yes	30	1	non-adherent	55%
	9	no	no	no	no	no	no	no	27	1	adherent	26%
	10	no	no	no	no	no	no	yes	27	1	adherent	28%
	11	no	no	no	no	no	no	no	18	1	non-adherent	68%
	12	yes	yes	no	no	no	no	no	28	0	adherent	24%
	13	yes	no	no	no	no	no	yes	31	1	non-adherent	52%
	14	no	yes	yes	no	no	no	yes	23	0	adherent	60%
	15	no	yes	no	no	no	yes	yes	26	1	non-adherent	38%
	16	no	no	no	no	no	yes	no	18	1	non-adherent	68%
	17	no	no	no	no	no	no	no	20	1	non-adherent	68%
	18	no	yes	no	no	no	yes	no	10	1	adherent	88%
	19	yes	no	no	no	no	no	no	7	0	adherent	92%
	20	yes	yes	no	no	no	no	yes	22	1	adherent	40%
	21	yes	yes	no	no	no	no	no	31	1	adherent	48%
	22	yes	no	no	yes	yes	no	no	33	1	adherent	20%

	23	yes	no	no	no	no	no	yes	28	0	non-adherent	40%
	24	yes	yes	yes	no	no	no	yes	25	1	adherent	40%
	25	yes	no	no	no	no	no	yes	27	1	adherent	28%
	26	no	no	no	no	no	yes	no	27	1	adherent	60%
	27	no	no	no	no	no	no	yes	20	0	adherent	56%
	28	yes	yes	no	yes	no	no	yes	17	1	non-adherent	52%
	29	yes	yes	no	no	no	yes	no	28	1	non-adherent	60%
	TotalN	29	29	29	29	29	29	29	29	29	29	29
2	1	no	no	yes	yes	no	no	no	30	3	non-adherent	55%
	2	no	no	yes	yes	no	no	no	30	3	non-adherent	52%
	3	no	yes	yes	yes	no	no	no	31	3	non-adherent	52%
	4	no	yes	yes	yes	no	no	no	33	3	non-adherent	50%
	5	no	yes	yes	yes	no	no	no	34	3	non-adherent	48%
	6	no	no	yes	yes	no	no	no	25	3	adherent	40%
	7	no	no	yes	yes	no	no	no	7	1	adherent	92%
	8	no	yes	yes	yes	no	no	no	7	1	adherent	92%
	9	no	yes	yes	no	no	no	no	7	1	adherent	92%
	10	yes	yes	yes	no	no	no	no	7	1	adherent	92%
	11	no	no	yes	no	yes	no	no	15	2	non-adherent	80%
	12	no	no	yes	yes	no	no	no	10	1	adherent	80%
	13	yes	yes	yes	yes	no	no	no	18	2	non-adherent	28%
	14	no	yes	yes	yes	no	no	yes	20	2	adherent	44%
	15	no	yes	yes	no	no	no	yes	22	2	adherent	44%
	16	yes	yes	yes	yes	no	no	no	20	2	adherent	80%
	17	no	yes	yes	no	no	no	yes	28	3	non-adherent	60%
	18	yes	yes	yes	yes	no	no	no	10	1	adherent	60%
	19	no	no	yes	yes	no	no	no	17	2	adherent	56%
	20	yes	yes	yes	yes	no	no	no	17	2	adherent	56%

	21	yes	yes	yes	no	no	no	no	26	2	adherent	44%
	22	no	yes	yes	yes	no	no	no	13	1	adherent	68%
	23	no	no	yes	yes	no	no	yes	16	1	adherent	68%
	24	yes	yes	yes	no	yes	no	no	8	1	adherent	80%
	25	no	no	no	no	no	no	no	8	1	adherent	80%
	26	no	yes	no	no	no	no	yes	10	1	adherent	80%
	27	no	yes	yes	yes	no	no	yes	10	1	adherent	80%
	28	no	yes	yes	no	no	no	no	23	2	non-adherent	60%
	29	no	no	yes	yes	no	no	no	18	2	non-adherent	52%
	30	no	yes	yes	yes	no	no	no	10	2	adherent	84%
	31	no	no	no	no	no	no	no	28	1	adherent	24%
	32	no	no	no	no	no	no	no	23	2	adherent	40%
	33	no	no	no	no	no	no	yes	26	2	non-adherent	38%
	34	no	no	no	no	no	no	no	26	1	non-adherent	36%
	35	no	no	no	no	no	no	no	27	1	adherent	28%
	36	no	no	no	no	no	no	yes	10	2	adherent	88%
	37	no	no	no	no	no	no	no	25	1	non-adherent	68%
	38	no	no	no	no	no	no	no	28	2	adherent	24%
	39	no	no	no	no	no	no	no	28	2	adherent	24%
	40	yes	yes	no	no	no	no	yes	23	1	adherent	60%
	41	no	no	no	no	no	no	no	25	1	adherent	50%
	42	no	no	no	no	no	no	no	27	2	adherent	26%
	43	yes	no	no	no	no	yes	no	21	2	adherent	60%
	44	no	no	no	no	no	no	no	7	1	adherent	92%
	45	no	no	no	no	no	no	yes	7	1	adherent	92%
	46	no	yes	yes	no	no	no	yes	15	2	adherent	32%
	47	no	yes	no	no	no	yes	yes	28	2	adherent	52%
	48	no	no	no	no	no	no	yes	30	2	non-adherent	60%

	49	no	no	no	no	no	no	no	34	1	adherent	72%
	50	no	no	no	no	no	no	no	25	2	adherent	52%
	51	no	yes	no	no	no	no	yes	25	2	adherent	60%
	52	no	no	no	no	no	no	no	24	1	non-adherent	36%
	53	no	yes	no	yes	no	yes	yes	26	2	adherent	26%
	54	no	no	no	no	no	no	no	27	1	non-adherent	68%
	55	no	yes	yes	no	no	no	yes	10	2	adherent	24%
	56	no	yes	yes	no	no	no	yes	29	1	adherent	55%
	57	yes	yes	yes	no	no	no	no	30	1	adherent	48%
	58	yes	yes	no	no	no	no	yes	33	2	adherent	28%
	59	yes	no	no	no	no	no	yes	34	1	adherent	40%
	60	yes	yes	no	no	no	no	no	25	1	adherent	60%
	61	no	no	no	no	no	no	no	27	2	adherent	68%
	62	no	yes	no	no	no	yes	yes	21	1	adherent	56%
	63	yes	yes	no	no	no	yes	no	10	1	adherent	52%
	64	yes	yes	yes	yes	no	no	no	34	2	non-adherent	52%
	65	no	yes	no	no	no	no	no	28	2	adherent	50%
	66	yes	no	no	no	no	no	no	25	1	non-adherent	40%
	67	yes	yes	no	no	no	no	yes	23	2	adherent	36%
	68	yes	no	no	yes	no	yes	yes	20	2	adherent	26%
	69	yes	yes	yes	no	no	yes	yes	26	2	adherent	68%
	TotalN	69	69	69	69	69	69	69	69	69	69	69
3	1	yes	yes	yes	yes	yes	no	no	22	3	adherent	60%
	2	yes	yes	yes	yes	no	no	no	28	3	adherent	24%
	3	yes	yes	yes	yes	no	no	no	28	3	adherent	24%
	4	yes	yes	yes	yes	no	no	no	28	3	adherent	24%
	5	yes	yes	yes	yes	no	no	no	28	4	adherent	24%
	6	no	no	yes	yes	no	no	no	17	3	adherent	72%

	7	no	no	yes	yes	no	no	no	28	3	non-adherent	28%
	8	no	no	yes	yes	no	no	no	25	3	adherent	40%
	9	no	no	yes	yes	no	no	yes	22	2	adherent	52%
	10	no	yes	no	no	no	no	yes	23	3	adherent	60%
	11	no	no	no	no	no	no	yes	23	3	adherent	60%
	12	no	no	no	no	no	no	no	20	2	adherent	48%
	13	yes	yes	yes	yes	no	no	yes	26	4	non-adherent	38%
	14	yes	yes	yes	yes	no	no	yes	26	4	non-adherent	36%
	15	no	yes	yes	no	no	no	yes	21	3	adherent	60%
	16	no	yes	yes	no	no	no	no	18	2	non-adherent	68%
	17	no	yes	yes	no	no	no	no	10	1	adherent	88%
	18	yes	yes	yes	no	no	no	no	29	4	adherent	56%
	19	no	no	yes	no	no	no	no	15	3	adherent	32%
	20	no	no	yes	yes	no	no	no	22	3	adherent	40%
	21	no	yes	yes	yes	no	no	yes	24	4	adherent	48%
	22	no	no	no	no	no	no	no	22	3	adherent	48%
	23	yes	yes	yes	yes	no	no	yes	25	3	non-adherent	80%
	24	yes	yes	yes	yes	no	no	yes	21	3	adherent	40%
	25	no	no	yes	yes	no	no	yes	22	3	adherent	40%
	26	no	no	yes	yes	no	no	yes	20	3	adherent	40%
	27	yes	yes	yes	yes	no	no	yes	22	3	non-adherent	40%
	28	yes	yes	yes	no	no	no	yes	21	3	non-adherent	40%
	29	no	no	yes	yes	no	no	yes	35	4	adherent	36%
	30	no	no	yes	yes	no	no	yes	35	4	adherent	80%
	31	no	no	yes	yes	no	no	yes	20	3	adherent	48%
	32	no	yes	yes	yes	no	no	yes	24	3	adherent	48%
	33	no	yes	yes	no	no	no	no	26	3	adherent	28%
	34	yes	yes	yes	yes	yes	yes	no	37	4	adherent	36%

35	no	yes	yes	no	no	no	no	25	4	adherent	40%
36	no	yes	yes	no	no	no	no	22	4	adherent	40%
37	no	no	no	no	no	no	yes	22	3	adherent	48%
38	no	yes	yes	yes	no	no	no	20	2	adherent	44%
39	no	yes	no	no	no	no	no	20	2	adherent	44%
40	no	yes	yes	yes	no	no	no	21	3	adherent	52%
41	no	yes	yes	yes	no	no	no	16	3	adherent	68%
42	no	yes	yes	yes	no	no	no	22	3	non-adherent	60%
43	no	no	yes	yes	no	no	no	21	3	adherent	64%
44	no	no	yes	yes	no	no	no	22	2	adherent	44%
45	no	no	yes	yes	no	no	yes	15	2	non-adherent	52%
46	yes	no	no	no	no	no	no	31	2	non-adherent	40%
47	yes	no	no	no	no	no	no	33	2	non-adherent	40%
48	no	no	no	no	no	no	yes	34	2	non-adherent	52%
49	yes	yes	yes	no	no	no	yes	34	3	non-adherent	60%
50	no	no	no	no	no	no	no	23	3	adherent	38%
51	no	no	no	no	no	no	no	23	2	adherent	36%
52	no	yes	no	no	no	yes	yes	25	3	adherent	50%
53	yes	yes	no	no	no	no	yes	20	3	adherent	48%
54	no	no	no	no	no	no	no	24	3	adherent	40%
55	yes	no	no	no	no	no	no	7	3	adherent	92%
56	no	no	no	no	no	no	no	15	3	adherent	32%
57	no	no	no	no	no	no	yes	23	3	non-adherent	20%
58	no	no	no	no	no	no	no	25	3	non-adherent	46%
59	no	no	no	no	no	no	no	22	3	adherent	40%
60	no	yes	no	no	no	no	yes	24	2	adherent	48%
61	no	no	no	no	no	no	no	15	3	non-adherent	80%
62	no	no	no	no	no	no	yes	10	2	adherent	80%

63	no	no	no	no	no	no	yes	22	3	non-adherent	68%
64	no	no	no	no	no	no	no	29	3	adherent	56%
65	yes	yes	yes	no	no	no	yes	30	3	adherent	92%
66	no	no	no	no	no	no	no	35	3	adherent	40%
67	no	no	no	no	no	yes	no	28	3	adherent	24%
68	no	no	no	no	no	no	yes	33	3	non-adherent	60%
69	yes	yes	no	no	no	no	no	34	3	non-adherent	48%
70	no	no	no	no	no	no	no	34	3	non-adherent	20%
71	no	no	no	no	no	no	yes	17	3	adherent	72%
72	yes	no	no	no	no	yes	yes	28	3	non-adherent	28%
73	no	no	no	no	no	no	no	25	3	adherent	40%
74	no	no	no	no	no	no	yes	25	3	adherent	40%
75	yes	yes	no	no	no	no	yes	20	3	adherent	48%
76	no	yes	no	no	no	yes	yes	26	3	non-adherent	36%
77	no	no	no	no	no	yes	yes	27	3	adherent	28%
78	no	no	no	no	no	no	yes	27	2	adherent	28%
79	yes	no	no	no	no	no	no	29	3	adherent	56%
80	no	no	no	no	no	no	no	28	3	adherent	24%
81	yes	yes	yes	no	no	no	no	22	3	adherent	60%
82	yes	yes	yes	no	no	no	no	23	3	non-adherent	38%
83	no	yes	yes	no	no	no	yes	26	3	adherent	28%
84	no	no	no	no	no	no	no	21	3	non-adherent	68%
85	no	no	no	no	no	yes	yes	30	3	adherent	20%
86	no	yes	no	no	no	yes	yes	31	3	adherent	72%
87	no	no	no	no	no	no	yes	25	3	adherent	50%
88	yes	no	no	no	no	no	no	22	3	adherent	48%
89	no	yes	no	yes	no	no	yes	25	3	non-adherent	38%
90	no	no	no	no	no	no	no	20	3	adherent	36%

	91	yes	yes	no	no	no	no	yes	24	3	adherent	26%
	92	yes	yes	no	no	no	no	yes	26	2	adherent	28%
	93	no	no	no	no	no	no	no	26	3	non-adherent	60%
	94	yes	no	no	no	no	no	yes	27	3	non-adherent	68%
	95	no	no	no	no	no	no	no	27	2	adherent	88%
	96	no	no	no	no	no	no	yes	20	3	adherent	55%
	97	yes	yes	yes	yes	no	no	yes	29	3	adherent	52%
	98	no	no	no	no	no	no	no	30	3	adherent	28%
	99	no	no	no	no	no	yes	yes	31	3	adherent	40%
	100	yes	no	no	no	no	no	yes	33	3	non-adherent	40%
	101	yes	no	no	no	no	no	no	34	3	adherent	60%
	102	yes	yes	yes	no	no	no	yes	17	3	adherent	60%
	103	no	yes	no	no	no	yes	no	23	3	adherent	38%
	104	yes	yes	no	no	no	no	no	25	3	adherent	28%
	105	yes	yes	yes	no	no	yes	yes	24	3	adherent	60%
	106	yes	yes	no	no	no	no	yes	26	3	non-adherent	68%
	TotalN	106	106	106	106	106	106	106	106	106	106	106
4	1	no	yes	yes	yes	yes	no	yes	34	4	non-adherent	20%
	2	yes	yes	yes	yes	no	no	yes	25	4	adherent	50%
	3	no	yes	no	no	no	no	no	23	3	adherent	40%
	4	no	yes	yes	no	no	no	no	24	3	adherent	40%
	5	no	yes	yes	yes	no	yes	yes	27	4	adherent	28%
	6	no	no	yes	yes	no	yes	yes	27	4	adherent	26%
	7	yes	yes	yes	yes	no	no	yes	27	4	adherent	28%
	8	yes	yes	yes	yes	no	no	no	20	3	non-adherent	68%
	9	no	no	no	no	no	no	no	23	3	non-adherent	20%
	10	no	yes	no	no	no	no	yes	25	4	non-adherent	46%
	11	yes	yes	yes	yes	no	no	no	22	3	non-adherent	84%

12	no	no	no	no	no	no	yes	29	3	adherent	48%
13	no	no	no	no	no	no	yes	29	3	adherent	44%
14	no	yes	yes	yes	no	no	yes	30	4	adherent	42%
15	no	no	yes	yes	no	no	yes	35	4	adherent	40%
16	no	no	yes	yes	no	no	yes	35	4	adherent	68%
17	no	yes	yes	yes	no	no	yes	26	3	adherent	48%
18	no	no	yes	no	no	no	yes	20	3	adherent	64%
19	no	no	yes	no	no	no	yes	20	3	adherent	64%
20	no	no	yes	no	no	no	yes	20	3	adherent	56%
21	no	no	yes	yes	no	no	yes	18	3	adherent	56%
22	no	yes	yes	no	no	no	yes	24	4	adherent	56%
23	no	yes	yes	yes	no	no	yes	24	4	adherent	56%
24	no	yes	yes	yes	no	no	no	20	4	adherent	52%
25	no	yes	yes	yes	no	no	no	24	4	adherent	52%
26	no	yes	yes	yes	no	no	yes	22	4	non-adherent	44%
27	no	no	yes	yes	no	no	no	21	3	adherent	68%
28	no	no	no	no	no	no	no	22	3	adherent	40%
29	no	no	no	no	no	no	no	22	3	adherent	40%
30	yes	yes	no	no	no	no	yes	28	4	adherent	36%
31	yes	yes	yes	yes	no	no	yes	28	4	adherent	36%
32	no	yes	yes	yes	no	no	no	24	3	non-adherent	70%
33	no	no	yes	yes	no	no	no	13	3	non-adherent	60%
34	no	no	yes	yes	no	no	yes	20	3	non-adherent	64%
35	no	no	no	no	no	no	no	28	4	adherent	24%
36	yes	yes	no	no	no	no	no	30	3	non-adherent	52%
37	no	no	no	no	no	no	no	17	3	adherent	60%
38	no	no	no	no	no	no	no	28	4	non-adherent	50%
39	no	no	no	no	no	no	yes	25	3	adherent	48%

40	no	no	no	no	no	no	no	25	3	adherent	40%
41	no	no	no	no	no	no	no	22	3	adherent	40%
42	yes	yes	yes	no	no	no	yes	21	4	adherent	60%
43	no	yes	yes	yes	yes	no	yes	20	3	non-adherent	68%
44	yes	yes	yes	no	no	no	no	29	3	adherent	56%
45	no	no	no	no	no	no	no	7	3	adherent	92%
46	no	no	no	no	no	no	yes	7	4	adherent	92%
47	yes	yes	no	no	no	no	yes	7	4	adherent	92%
48	no	no	no	no	no	no	no	22	3	adherent	48%
49	no	no	no	no	no	no	yes	29	4	adherent	88%
50	no	yes	yes	no	no	no	no	22	4	adherent	55%
51	yes	yes	no	yes	no	no	no	30	4	non-adherent	55%
52	yes	no	no	no	no	yes	yes	30	4	non-adherent	52%
53	no	yes	no	yes	yes	no	no	22	3	adherent	52%
54	no	no	no	no	no	no	yes	23	4	adherent	40%
55	no	yes	no	no	no	yes	yes	24	4	adherent	40%
56	no	no	no	no	no	no	no	7	4	adherent	92%
57	yes	no	no	yes	no	no	yes	23	4	non-adherent	20%
58	no	no	no	no	no	no	yes	25	4	non-adherent	46%
59	no	no	no	no	no	no	no	28	3	adherent	55%
60	no	no	no	no	no	no	no	30	3	non-adherent	52%
61	yes	yes	no	no	no	no	yes	34	3	adherent	28%
62	no	no	no	no	no	no	no	17	4	non-adherent	40%
63	yes	no	no	no	no	no	no	23	4	adherent	50%
64	yes	no	no	no	no	no	no	23	4	adherent	48%
65	yes	no	no	no	no	no	yes	20	3	adherent	40%
66	yes	no	no	no	no	yes	yes	18	4	adherent	88%
67	no	no	no	no	no	no	no	28	3	non-adherent	52%

	68	no	no	no	no	no	no	no	28	4	non-adherent	52%
	69	no	yes	no	no	no	no	yes	28	4	adherent	60%
	70	yes	yes	no	yes	no	no	yes	34	3	adherent	40%
	71	no	no	no	no	no	no	no	23	4	adherent	40%
	72	yes	no	no	no	no	yes	yes	23	4	non-adherent	40%
	73	no	no	no	no	no	no	yes	23	3	adherent	28%
	74	no	no	no	no	no	yes	yes	18	4	adherent	24%
	75	no	yes	no	no	yes	yes	yes	28	3	non-adherent	60%
	76	no	no	no	no	no	no	yes	28	4	non-adherent	48%
	77	no	yes	yes	no	no	no	yes	28	4	adherent	20%
	78	yes	yes	no	no	no	no	no	30	3	adherent	72%
	79	no	no	no	no	no	no	no	25	3	adherent	48%
	80	yes	yes	no	no	no	yes	yes	22	4	non-adherent	40%
	81	no	yes	no	yes	no	no	no	23	4	adherent	28%
	TotalN	81	81	81	81	81	81	81	81	81	81	81
TotalN		285	285	285	285	285	285	285	285	285	285	285

a. Limited to first 500 cases.

Case Summaries[a]

		BP	ILP	PC	AO	PN	PmN	Bron-chitis	Fibro-sis	Mixed alveolar dysfunction	Distal airway obstruction	Reversible airway obstruction	FEV1	FVC	FEV1/FVC
GOLD 1	1	no	no	no	no	yes	yes	no	no	no	yes	no	16.4	54.8	30.00%
	2	no	no	no	no	no	no	no	no	yes	no	no	30.5	45.1	67.70%
	3	yes	no	no	no	no	no	no	no	no	yes	no	32.0	76.0	42.10%
	4	no	yes	no	no	no	no	no	no	yes	yes	no	49.0	59.0	83.00%
	5	yes	yes	yes	no	yes	yes	no	no	yes	yes	no	21.8	42.4	51.50%

6	yes	no	no	no	no	no	no	no	no	yes	no	24.8	69.8	35.50%
7	no	no	no	no	no	no	no	no	no	yes	no	18.1	52.2	34.60%
8	no	no	no	no	no	no	no	no	no	yes	no	72.0	81.6	88.30%
9	yes	no	no	no	yes	yes	yes	no	no	yes	no	22.0	34.6	63.60%
10	yes	no	no	no	no	no	no	no	no	yes	no	48.5	90.1	53.80%
11	no	no	yes	no	yes	no	no	no	no	yes	no	92.6	130.2	71.10%
12	yes	yes	no	no	no	no	no	no	no	yes	no	54.0	67.4	80.10%
13	no	no	no	no	no	no	yes	no	no	yes	no	51.4	64.1	80.20%
14	yes	no	no	no	no	no	no	no	no	yes	no	41.7	73.0	57.20%
15	yes	no	no	no	no	no	no	no	no	yes	no	66.1	98.2	67.30%
16	yes	no	yes	no	yes	no	no	yes	no	yes	no	93.2	123.4	75.50%
17	yes	no	no	no	yes	no	no	no	no	yes	no	50.1	69.0	72.50%
18	yes	yes	no	no	no	no	no	no	no	yes	no	68.6	76.0	90.20%
19	yes	no	no	yes	yes	yes	yes	no	no	yes	no	25.5	42.8	59.50%
20	yes	yes	no	no	no	no	no	no	no	yes	no	42.6	48.8	87.40%
21	yes	no	no	yes	no	no	no	no	no	yes	no	74.1	90.4	82.00%
22	yes	no	no	no	yes	no	no	no	yes	yes	yes	49.3	62.9	78.40%
23	yes	yes	no	no	no	no	no	no	no	yes	no	41.8	52.3	79.80%
24	yes	no	yes	no	yes	no	yes	yes	no	yes	no	80.7	87.9	91.80%
25	yes	no	no	no	no	no	no	no	no	yes	no	71.4	82.2	86.90%
26	yes	no	no	yes	no	no	no	no	no	yes	no	60.2	87.2	69.00%
27	yes	yes	no	no	no	no	no	no	no	yes	no	32.9	58.8	55.90%
28	yes	yes	no	no	yes	yes	no	no	no	yes	no	64.2	85.2	75.30%
29	yes	yes	yes	no	yes	yes	no	no	no	yes	no	44.7	54.6	81.90%
Total N	29	29	29	29	29	29	29	29	29	29	29	29	29	29
Minimum	no	no	no	no	no	no	no	no	no	no	no	16.4	34.6	30.00%
Maximum	yes	yes	yes	yes	yes	yes	yes	yes	yes	yes	yes	93.2	130.2	91.80%

	Mean	.76	.31	.17	.10	.38	.21	.14	.07	.14	.97	.03	49.662	71.034	68.6931%
	Median	1.00	.00	.00	.00	.00	.00	.00	.00	.00	1.00	.00	49.000	69.000	72.5000%
2	1	no	no	no	no	no	no	no	no	no	yes	no	65.0	102.0	63.70%
	2	no	no	no	no	no	no	no	no	no	yes	no	65.0	102.0	63.70%
	3	no	no	no	no	no	no	no	no	no	yes	no	65.0	102.0	63.70%
	4	no	no	no	no	no	no	no	no	no	yes	no	65.0	102.0	63.70%
	5	no	yes	no	no	no	no	no	no	no	yes	no	65.0	102.0	63.70%
	6	yes	no	no	no	no	no	no	no	no	no	no	65.0	102.0	63.70%
	7	yes	no	no	no	no	no	yes	no	no	yes	no	63.6	100.7	63.20%
	8	yes	no	no	no	no	no	yes	no	no	yes	no	51.2	87.0	58.90%
	9	yes	no	no	no	no	no	no	no	yes	yes	no	50.1	66.8	75.00%
	10	yes	no	no	no	yes	yes	no	no	no	no	no	68.3	109.8	62.20%
	11	yes	no	no	no	yes	yes	no	no	yes	yes	no	36.5	81.1	45.00%
	12	yes	no	no	no	no	no	no	yes	yes	yes	no	40.2	80.3	50.10%
	13	no	no	no	no	no	no	no	no	no	yes	no	16.6	42.5	39.00%
	14	yes	no	no	no	yes	yes	no	no	yes	yes	no	29.2	52.0	56.20%
	15	yes	no	no	no	yes	yes	no	no	no	yes	no	35.2	67.0	52.60%
	16	no	no	no	no	yes	yes	no	no	yes	yes	no	47.1	54.1	87.00%
	17	yes	no	no	no	no	no	no	no	no	yes	no	33.9	57.9	58.50%
	18	no	no	yes	no	yes	no	no	yes	yes	yes	no	16.5	27.5	59.80%
	19	yes	no	no	no	no	no	no	no	no	yes	no	37.3	53.5	69.70%
	20	yes	no	no	no	no	no	no	no	no	yes	no	76.4	88.0	86.70%
	21	yes	yes	no	no	no	no	no	no	yes	yes	no	32.6	51.2	63.70%
	22	yes	no	no	no	no	no	no	no	no	no	no	42.2	60.3	70.00%
	23	yes	no	no	no	no	no	no	no	no	yes	no	36.9	55.5	66.40%
	24	yes	no	no	no	yes	yes	no	yes	no	no	no	32.4	75.4	43.00%
	25	yes	no	no	no	no	no	no	no	yes	yes	no	86.7	111.3	77.90%

26	yes	no	no	no	no	no	no	no	yes	yes	no	81.2	105.8	76.80%
27	no	yes	no	no	no	no	no	no	yes	yes	no	57.9	92.3	62.70%
28	yes	no	no	no	no	no	no	no	no	yes	no	43.5	51.4	84.60%
29	yes	no	no	no	no	no	no	no	no	yes	no	36.8	61.4	59.90%
30	yes	no	no	no	no	no	no	no	no	no	no	91.4	108.5	84.20%
31	yes	yes	no	no	no	no	no	no	no	yes	no	71.2	115.0	61.90%
32	yes	yes	no	no	yes	no	yes	no	no	yes	no	35.7	59.6	59.90%
33	yes	no	yes	yes	yes	yes	yes	yes	no	yes	no	38.1	80.0	47.70%
34	yes	yes	no	no	no	no	no	no	no	yes	no	16.5	50.6	32.60%
35	yes	no	no	no	no	no	no	no	no	yes	no	37.7	68.4	55.10%
36	yes	no	no	no	no	no	no	yes	no	yes	no	71.2	85.5	83.20%
37	yes	yes	no	no	no	no	no	no	no	yes	no	44.8	79.7	56.20%
38	yes	no	no	no	no	no	no	no	no	yes	no	97.2	103.3	94.00%
39	yes	yes	no	no	yes	no	yes	no	yes	yes	no	89.4	109.6	81.60%
40	yes	yes	yes	no	yes	yes	no	no	no	yes	no	35.3	65.3	54.10%
41	no	no	yes	no	yes	yes	yes	no	no	yes	no	29.8	51.2	58.20%
42	yes	yes	no	no	no	no	yes	no	no	yes	no	46.8	55.7	84.00%
43	yes	no	no	yes	no	no	no	no	no	yes	no	20.4	63.0	32.50%
44	no	no	no	no	yes	yes	no	no	no	yes	no	80.4	120.3	66.80%
45	yes	no	yes	no	no	no	yes	no	no	yes	no	41.9	70.1	59.70%
46	yes	yes	no	no	yes	no	no	no	no	yes	no	42.9	68.9	62.30%
47	yes	no	no	no	no	no	no	no	no	yes	no	23.2	43.9	52.80%
48	yes	no	no	no	yes	yes	no	no	no	yes	no	55.1	94.9	58.00%
49	yes	yes	yes	no	no	no	no	no	yes	yes	no	27.3	61.4	44.40%
50	no	no	no	no	no	no	no	no	no	yes	no	43.3	57.8	75.00%
51	yes	no	no	no	yes	yes	no	no	no	yes	no	53.4	91.8	58.20%
52	yes	yes	no	no	yes	no	yes	no	yes	yes	no	18.0	50.0	36.00%
53	yes	yes	no	no	no	no	no	no	no	yes	no	68.9	74.9	92.00%

	54	yes	yes	yes	no	no	no	yes	no	no	yes	no	20.3	36.3	55.90%
	55	yes	yes	yes	no	yes	yes	no	no	no	yes	no	16.8	34.5	48.80%
	56	no	yes	no	no	no	no	no	no	no	yes	no	62.0	101.3	61.20%
	57	yes	no	no	no	no	no	no	no	no	yes	no	82.7	99.5	83.10%
	58	yes	no	no	no	yes	no	no	no	no	yes	no	28.1	65.8	42.70%
	59	yes	yes	no	no	no	no	no	no	no	yes	no	64.0	94.2	68.00%
	60	yes	yes	no	no	yes	no	no	no	no	yes	no	23.5	64.6	36.40%
	61	yes	no	no	no	yes	yes	no	no	no	yes	no	16.8	35.4	47.50%
	62	yes	yes	no	no	no	no	no	no	no	yes	no	63.8	71.5	89.10%
	63	yes	yes	no	no	yes	no	yes	no	no	yes	no	17.1	39.5	43.30%
	64	yes	no	no	yes	no	no	no	yes	no	yes	no	86.3	120.4	71.70%
	65	yes	no	yes	no	no	no	no	no	no	yes	no	35.4	58.7	60.40%
	66	yes	no	no	no	no	no	no	no	no	yes	no	15.0	51.1	29.40%
	67	yes	yes	no	no	no	no	no	no	no	yes	no	21.9	43.3	50.60%
	68	no	no	no	no	no	no	no	no	no	yes	no	38.4	77.3	49.70%
	69	yes	no	no	no	yes	no	no	no	no	yes	no	57.0	63.6	89.60%
Total	N	69	69	69	69	69	69	69	69	69	69	69	69	69	69
	Minimum	no	no	no	no	no	no	no	no	no	no	no	15.0	27.5	29.40%
	Maximum	yes	yes	yes	yes	yes	yes	yes	yes	yes	yes	no	97.2	120.4	94.00%
	Mean	.80	.32	.13	.04	.33	.20	.16	.09	.19	.93	.00	47.410	74.365	61.8681%
	Median	1.00	.00	.00	.00	.00	.00	.00	.00	.00	1.00	.00	42.900	68.900	61.2000%
3	1	yes	yes	no	no	no	no	no	no	yes	yes	no	40.0	82.0	48.80%
	2	yes	no	no	no	no	no	no	no	yes	yes	no	40.0	82.0	48.80%
	3	yes	no	no	no	no	no	no	no	yes	yes	no	40.0	82.0	48.80%
	4	yes	no	no	no	no	no	no	no	yes	yes	no	40.0	82.0	48.80%
	5	yes	no	no	no	no	no	no	no	yes	yes	no	40.0	82.0	48.80%
	6	yes	no	no	no	no	no	no	no	no	yes	no	40.0	82.0	48.80%

7	yes	no	no	no	no	no	no	no	no	yes	no	40.0	82.0	48.80%
8	yes	no	no	no	no	no	no	no	no	yes	no	40.0	82.0	48.80%
9	yes	no	no	no	no	no	no	no	no	yes	no	40.0	82.0	48.80%
10	yes	no	no	no	no	no	no	no	yes	yes	no	40.0	82.0	48.80%
11	yes	no	no	no	no	no	no	no	yes	no	no	40.0	82.0	48.80%
12	no	no	no	no	no	no	no	no	no	yes	no	40.0	82.0	48.80%
13	yes	no	no	no	no	no	no	no	yes	yes	no	40.0	82.0	48.80%
14	yes	no	no	no	no	no	no	no	yes	yes	no	40.0	82.0	48.80%
15	yes	no	no	yes	no	no	no	yes	no	yes	no	32.7	73.2	44.70%
16	no	yes	no	no	no	no	no	no	yes	yes	no	48.6	71.4	68.00%
17	no	no	no	no	no	no	no	no	no	yes	no	43.1	67.8	63.60%
18	yes	yes	no	no	no	no	no	no	yes	yes	yes	49.2	86.2	57.00%
19	yes	no	no	no	no	no	no	yes	no	yes	no	59.0	73.9	79.80%
20	yes	no	no	no	no	no	no	no	yes	no	no	48.2	91.1	53.00%
21	yes	no	no	no	yes	yes	no	yes	yes	yes	no	58.3	84.1	69.30%
22	yes	no	no	no	yes	yes	no	no	yes	yes	no	33.1	38.7	85.60%
23	no	no	no	no	yes	no	no	no	yes	no	no	22.0	57.5	38.30%
24	no	yes	no	no	no	no	yes	no	yes	yes	no	35.5	54.1	65.60%
25	yes	no	no	no	no	no	no	no	yes	yes	no	24.7	65.6	37.60%
26	yes	no	no	no	no	no	no	no	yes	yes	no	16.5	34.0	48.60%
27	yes	no	no	no	no	no	no	yes	yes	yes	no	31.6	74.1	42.60%
28	yes	no	no	no	yes	yes	no	yes	yes	yes	no	49.1	61.7	79.50%
29	yes	no	no	no	no	no	no	no	yes	yes	no	37.5	81.9	45.80%
30	yes	no	no	no	no	no	no	no	yes	yes	no	69.9	114.6	61.00%
31	no	no	no	no	no	no	no	no	no	yes	no	63.2	95.1	66.40%
32	no	no	no	no	no	no	no	no	no	yes	no	69.5	89.9	77.40%
33	yes	no	no	no	no	no	no	no	no	yes	no	43.3	77.1	56.20%
34	no	no	no	no	yes	yes	yes	no	yes	yes	no	26.7	44.5	59.90%

35	yes	yes	no	no	no	no	no	no	yes	yes	no	57.0	76.4	74.70%
36	yes	no	no	no	no	no	no	no	yes	yes	no	54.3	64.8	83.90%
37	yes	no	no	no	no	no	no	no	yes	yes	no	25.9	57.9	44.70%
38	no	no	no	no	no	no	no	no	yes	no	no	67.0	80.6	83.10%
39	no	no	no	no	no	no	no	no	yes	yes	no	68.6	89.0	77.00%
40	yes	no	no	no	no	no	no	no	yes	yes	no	73.9	89.7	82.40%
41	yes	no	no	no	no	no	no	no	yes	yes	no	56.9	95.9	59.30%
42	yes	no	no	no	no	no	no	no	yes	yes	no	96.4	118.6	81.30%
43	yes	no	no	no	yes	no	no	no	no	yes	no	60.8	96.7	62.90%
44	no	no	no	no	no	no	no	no	no	no	no	39.3	79.0	49.70%
45	yes	no	no	no	no	no	no	no	no	no	no	20.6	31.7	64.90%
46	yes	no	no	no	yes	no	no	no	no	yes	no	77.2	121.9	63.30%
47	no	no	no	no	no	no	no	yes	no	yes	no	29.3	43.8	67.00%
48	yes	no	no	no	yes	yes	no	no	no	yes	no	58.7	95.6	61.50%
49	no	no	no	yes	yes	no	no	no	yes	yes	no	20.1	31.4	64.10%
50	no	no	no	no	no	no	no	no	no	no	no	29.3	62.0	47.30%
51	yes	yes	no	no	no	no	no	no	no	no	no	29.8	49.9	59.70%
52	no	yes	no	no	yes	yes	no	yes	no	yes	no	33.6	67.3	49.90%
53	yes	yes	no	no	no	no	no	no	no	yes	no	33.1	49.3	67.10%
54	yes	no	no	no	no	no	no	no	no	yes	no	69.9	108.4	64.50%
55	yes	no	no	no	yes	no	yes	no	no	yes	no	29.4	57.5	51.10%
56	no	no	no	no	no	no	no	no	no	yes	no	36.0	64.8	55.50%
57	no	yes	no	no	yes	yes	yes	no	no	yes	no	37.2	45.5	81.70%
58	no	no	no	no	no	no	no	no	no	no	no	21.4	53.5	40.00%
59	yes	no	no	no	no	no	no	no	no	no	no	18.2	27.7	66.00%
60	yes	yes	yes	no	yes	no	no	no	no	yes	no	29.2	68.9	42.40%
61	yes	yes	no	no	yes	yes	yes	no	no	yes	no	18.0	45.4	39.70%
62	no	no	no	no	yes	yes	no	no	no	yes	no	47.0	73.1	64.30%

63	yes	no	no	no	no	no	no	no	no	yes	no	36.5	78.4	46.60%
64	yes	no	yes	no	no	no	no	no	no	no	no	59.3	95.2	62.30%
65	no	no	no	no	no	no	no	yes	no	yes	no	45.9	59.7	76.90%
66	no	no	yes	no	yes	yes	no	no	no	yes	no	64.5	99.1	65.10%
67	no	no	no	no	no	no	no	no	no	yes	no	29.3	68.5	42.80%
68	yes	no	no	no	no	no	no	no	no	yes	no	29.2	40.1	72.90%
69	yes	no	no	no	no	no	no	no	no	yes	no	18.0	35.4	51.00%
70	yes	no	no	no	yes	no	no	no	no	yes	no	36.2	56.5	64.00%
71	yes	no	no	no	no	no	no	no	no	yes	no	17.3	30.5	56.60%
72	yes	yes	no	no	yes	no	no	no	no	yes	no	25.0	31.9	78.20%
73	no	yes	no	no	yes	no	no	no	yes	yes	no	41.0	57.3	71.60%
74	no	yes	no	no	no	no	no	no	no	yes	no	36.3	78.2	46.40%
75	no	no	yes	no	yes	yes	yes	yes	no	yes	no	48.9	72.7	67.20%
76	yes	yes	no	no	no	no	no	no	no	yes	no	18.1	32.0	56.50%
77	no	no	no	no	no	no	yes	no	no	yes	no	46.6	80.2	58.10%
78	yes	no	no	no	yes	yes	no	no	no	yes	no	30.4	61.2	49.60%
79	no	no	no	no	no	no	no	no	no	yes	no	47.0	52.9	88.90%
80	yes	yes	yes	no	yes	no	yes	yes	no	yes	no	40.5	46.2	87.70%
81	yes	yes	no	no	no	no	no	no	no	yes	no	44.4	61.5	72.30%
82	yes	no	no	no	no	no	no	no	no	yes	no	40.6	78.1	51.90%
83	yes	yes	no	no	no	no	no	no	yes	yes	no	88.4	107.5	82.20%
84	yes	no	no	no	no	no	no	no	no	yes	no	29.0	67.2	43.10%
85	yes	no	no	no	no	no	no	no	no	yes	no	53.1	91.4	58.10%
86	yes	no	no	no	no	no	no	no	no	yes	no	23.2	35.2	66.00%
87	yes	no	yes	no	yes	yes	no	no	no	yes	no	21.9	40.7	53.90%
88	no	no	no	no	no	no	no	no	no	yes	no	92.9	137.7	67.50%
89	no	yes	yes	no	yes	yes	no	no	no	yes	no	62.9	72.5	86.80%
90	no	no	yes	no	yes	yes	no	no	no	yes	no	40.0	49.7	80.50%

	91		yes	yes	no	no	no	no	no	no	no	yes	no	86.2	105.5	81.70%
	92		yes	yes	yes	no	no	no	no	no	no	yes	no	93.8	136.7	68.60%
	93		no	no	no	no	yes	no	yes	no	no	yes	no	57.1	99.8	57.20%
	94		no	yes	no	no	no	no	no	no	no	yes	no	31.9	60.2	53.00%
	95		yes	no	no	no	no	no	no	no	no	yes	no	105.7	124.5	84.90%
	96		yes	yes	no	yes	yes	no	no	no	no	yes	no	17.5	29.9	58.70%
	97		yes	yes	yes	no	yes	yes	no	no	no	yes	no	16.1	26.2	61.60%
	98		yes	no	no	no	no	no	no	no	no	yes	no	23.3	61.6	37.80%
	99		yes	yes	no	no	no	no	no	no	no	yes	no	54.7	97.0	56.40%
	100		yes	no	no	no	no	no	no	no	no	yes	no	80.7	115.4	70.00%
	101		yes	no	no	no	no	no	no	no	no	yes	no	76.9	86.8	88.50%
	102		yes	no	no	no	no	no	no	no	no	yes	no	32.9	59.9	55.00%
	103		yes	yes	no	no	no	no	no	no	no	yes	no	45.6	60.9	74.80%
	104		yes	no	no	no	yes	no	no	no	no	yes	no	42.8	53.4	80.10%
	105		yes	yes	no	no	no	no	no	no	no	yes	no	39.6	83.7	47.30%
	106		no	yes	no	no	no	no	no	no	yes	yes	no	40.6	80.1	50.70%
	Total	N	106	106	106	106	106	106	106	106	106	106	106	106	106	106
		Minimum	no	no	no	no	no	no	no	no	no	no	no	16.1	26.2	37.60%
		Maximum	yes	yes	yes	yes	yes	yes	yes	yes	yes	yes	yes	105.7	137.7	88.90%
		Mean	.69	.26	.09	.03	.27	.15	.08	.09	.33	.90	.01	44.072	71.980	60.9726%
		Median	1.00	.00	.00	.00	.00	.00	.00	.00	.00	1.00	.00	40.000	73.550	59.5000%
4	1		no	no	no	no	no	no	no	no	yes	yes	no	22.0	64.0	34.40%
	2		yes	no	no	no	no	no	no	no	yes	yes	no	22.0	64.0	34.40%
	3		no	no	no	no	no	no	no	no	no	yes	no	22.0	64.0	34.40%
	4		no	no	no	no	no	no	no	no	no	yes	no	22.0	64.0	34.40%
	5		no	no	no	no	yes	yes	no	no	yes	yes	no	22.0	64.0	34.40%
	6		no	no	no	no	yes	yes	no	no	yes	yes	no	22.0	64.0	34.40%

7	no	no	no	no	yes	yes	no	no	yes	yes	no	22.0	64.0	34.40%
8	yes	no	no	no	yes	yes	no	no	yes	yes	no	37.7	53.0	71.10%
9	yes	no	no	no	yes	no	no	no	yes	no	no	24.9	69.4	35.90%
10	yes	no	no	no	yes	no	no	no	yes	yes	no	39.3	49.3	79.80%
11	yes	no	no	no	yes	yes	no	no	yes	yes	no	31.3	47.7	65.70%
12	no	no	no	no	no	no	no	no	yes	yes	no	48.4	69.1	70.00%
13	no	no	no	no	no	no	no	no	yes	yes	no	28.8	53.8	53.60%
14	no	no	no	no	no	no	no	no	yes	yes	no	27.0	67.8	39.70%
15	no	no	no	no	no	no	no	no	yes	yes	no	39.6	78.5	50.40%
16	yes	no	no	no	no	no	no	no	yes	yes	no	79.1	91.4	86.60%
17	yes	no	no	no	no	no	yes	no	yes	yes	no	32.9	39.1	84.20%
18	yes	no	no	no	no	no	no	no	yes	yes	no	21.6	31.6	68.40%
19	yes	no	no	no	no	no	no	no	yes	yes	no	17.6	47.0	37.50%
20	yes	no	no	no	no	no	no	no	yes	yes	no	19.1	52.6	36.20%
21	yes	no	no	no	no	no	no	no	yes	yes	no	40.0	78.9	50.70%
22	yes	yes	no	no	yes	no	no	yes	yes	yes	no	37.7	50.2	75.00%
23	no	no	no	no	yes	no	yes	yes	yes	yes	no	49.1	59.0	83.20%
24	yes	no	no	no	no	no	no	no	no	yes	no	16.3	45.4	35.90%
25	yes	no	no	no	no	no	no	no	no	yes	no	41.9	51.9	80.60%
26	yes	no	no	no	yes	yes	no	no	yes	yes	no	60.5	103.9	58.20%
27	yes	no	no	no	no	no	no	no	yes	yes	no	39.9	82.0	48.70%
28	yes	no	no	no	no	no	no	no	no	yes	no	57.8	70.3	82.30%
29	yes	no	no	no	no	no	no	no	no	yes	no	24.6	32.1	76.70%
30	yes	no	yes	no	yes	no	no	no	yes	yes	no	26.7	33.1	80.60%
31	yes	no	yes	no	yes	no	no	no	yes	yes	no	24.1	52.7	45.70%
32	yes	no	no	no	no	no	no	no	yes	yes	no	15.7	27.8	56.50%
33	yes	no	no	no	no	no	no	no	no	no	no	24.2	58.0	41.80%
34	no	no	no	no	no	no	no	no	no	no	no	30.5	64.3	47.40%

35	yes	no	no	no	no	no	yes	no	no	yes	no	70.0	96.3	72.70%
36	yes	yes	no	yes	no	no	no	no	no	yes	no	68.4	85.8	79.70%
37	yes	no	no	no	no	no	no	no	no	no	no	68.7	82.2	83.60%
38	yes	no	no	no	no	no	no	no	no	yes	no	23.1	63.1	36.60%
39	no	no	no	no	yes	yes	yes	no	no	yes	no	23.6	45.1	52.30%
40	yes	no	no	no	yes	yes	no	no	no	yes	no	71.0	112.8	63.00%
41	no	no	no	no	no	no	no	no	no	yes	no	22.2	36.8	60.30%
42	no	no	yes	no	yes	yes	no	no	no	yes	no	91.9	117.2	78.40%
43	yes	yes	yes	no	yes	yes	no	no	yes	yes	yes	84.9	101.3	83.90%
44	yes	no	no	no	no	no	no	no	no	yes	no	25.8	60.5	42.70%
45	no	yes	no	no	no	no	no	no	no	no	no	85.7	105.5	81.30%
46	yes	yes	yes	no	no	no	no	no	no	yes	no	48.2	77.5	62.10%
47	yes	yes	no	no	yes	no	no	no	no	yes	no	44.7	76.0	58.80%
48	yes	no	no	no	no	no	no	no	no	yes	no	19.8	60.4	32.70%
49	no	yes	no	no	yes	no	no	no	no	yes	no	47.3	80.9	58.40%
50	no	no	yes	no	yes	yes	no	no	no	yes	no	71.0	89.2	79.60%
51	yes	no	yes	no	yes	yes	no	no	no	yes	no	83.7	115.9	72.20%
52	yes	yes	yes	no	yes	yes	no	yes	no	yes	no	18.9	51.9	36.40%
53	yes	yes	no	no	no	no	no	no	no	yes	no	37.6	80.6	46.60%
54	yes	no	no	no	yes	no	yes	no	no	yes	no	39.1	80.3	48.70%
55	no	yes	yes	no	yes	yes	no	no	no	yes	no	47.0	55.9	84.10%
56	yes	yes	yes	no	yes	no	no	yes	no	yes	no	25.3	64.1	39.40%
57	yes	no	no	no	yes	yes	no	no	no	yes	no	44.2	84.3	52.50%
58	yes	yes	no	no	no	no	no	no	no	yes	no	31.2	58.1	53.70%
59	yes	no	no	no	yes	yes	yes	no	no	yes	no	31.5	76.0	41.40%
60	yes	yes	no	no	yes	yes	yes	yes	no	yes	no	98.2	113.7	86.30%
61	yes	yes	no	no	no	no	no	no	no	yes	no	26.7	44.7	59.80%
62	yes	yes	yes	no	yes	no	yes	no	no	yes	no	98.8	142.2	69.50%

	63		yes	no	no	no	no	no	no	no	yes	yes	no	55.5	100.0	55.50%
	64		yes	no	no	no	no	no	yes	no	no	yes	no	87.8	108.0	81.30%
	65		yes	yes	no	no	no	no	yes	no	no	yes	no	93.1	100.0	93.10%
	66		no	no	no	no	no	no	no	no	no	yes	no	88.7	95.7	92.70%
	67		yes	no	no	no	yes	yes	no	no	no	yes	no	16.6	54.9	30.20%
	68		yes	yes	no	no	yes	no	yes	no	no	yes	no	72.5	85.4	84.80%
	69		no	no	no	no	no	no	no	no	no	yes	no	85.9	108.1	79.50%
	70		yes	yes	no	no	no	no	no	no	no	no	no	87.8	127.6	68.80%
	71		no	yes	no	no	no	no	yes	no	no	yes	no	18.1	40.6	44.70%
	72		yes	no	no	no	no	no	no	no	no	yes	no	35.4	74.2	47.60%
	73		no	yes	no	no	no	no	no	no	no	yes	no	60.4	101.6	59.40%
	74		yes	no	no	no	no	no	no	no	no	no	no	60.4	72.9	82.80%
	75		yes	no	yes	no	no	no	no	no	no	yes	no	73.6	95.9	76.70%
	76		yes	no	no	no	yes	yes	no	no	no	yes	no	44.5	58.8	75.70%
	77		no	no	no	yes	no	no	no	no	no	yes	no	39.6	71.0	55.70%
	78		no	no	no	no	no	no	no	no	no	yes	no	60.7	91.6	66.20%
	79		yes	no	yes	no	yes	no	no	no	no	yes	no	17.9	62.0	28.80%
	80		yes	yes	no	no	yes	no	no	yes	no	yes	no	31.7	55.1	57.50%
	81		yes	yes	no	no	yes	no	yes	no	no	yes	no	46.2	65.6	70.40%
	Total	N	81	81	81	81	81	81	81	81	81	81	81	81	81	81
		Minimum	no	no	no	no	no	no	no	no	no	no	no	15.7	27.8	28.80%
		Maximum	yes	yes	yes	yes	yes	yes	yes	yes	yes	yes	yes	98.8	142.2	93.10%
		Mean	.69	.27	.16	.02	.42	.23	.16	.07	.35	.91	.01	44.632	72.040	59.8926%
		Median	1.00	.00	.00	.00	.00	.00	.00	.00	.00	1.00	.00	39.100	65.600	58.8000%
Total	N		285	285	285	285	285	285	285	285	285	285	285	285	285	285
	Minimum		no	no	no	no	no	no	no	no	no	no	no	15.0	26.2	28.80%
	Maximum		yes	yes	yes	yes	yes	yes	yes	yes	yes	yes	yes	105.7	142.2	94.00%

Mean	.72	.28	.13	.04	.34	.19	.13	.08	.28	.92	.01	45.608	72.478	61.6681%
Median	1.00	.00	.00	.00	.00	.00	.00	.00	.00	1.00	.00	40.000	69.800	61.0000%

a. Limited to first 500 cases.

Case summaries for comorbidities

Case Summaries[a]

		Respiratory failure	Other_pulmonary_diseases	Pulmonary_embolism	Heart_disease	Cardiac_failure	Effort_related_fatigue	Diabetes	Atrial_fibrilation_flutter
GO 1 LD	1	no	no	no	no	no	no	yes	no
	2	no	no	no	no	no	no	yes	no
	3	no	no	no	no	no	no	no	no
	4	no	no	no	no	no	no	no	no
	5	yes	yes	no	no	no	no	yes	no
	6	yes	yes	no	yes	no	no	yes	no
	7	no	yes	yes	no	no	no	no	no
	8	no	no	no	no	no	no	yes	no
	9	no	no	no	no	no	no	yes	no
	10	no	no	no	no	yes	no	yes	no
	11	no	no	yes	no	yes	no	no	yes
	12	no	no	no	yes	no	no	yes	no
	13	no	no	no	no	yes	no	yes	no
	14	no	no	no	yes	yes	no	no	no
	15	no	no	yes	no	yes	no	no	no
	16	no	no	yes	no	yes	no	yes	yes
	17	no	no	no	yes	yes	no	yes	yes
	18	no	no	no	yes	yes	no	yes	no
	19	no	no	no	no	yes	no	yes	no
	20	no	no	no	no	yes	no	yes	no
	21	no	no	no	no	yes	no	yes	no
	22	no	no	yes	no	yes	no	yes	yes
	23	no	no	no	no	yes	no	yes	no
	24	no	yes	no	yes	yes	no	yes	no
	25	no	no	no	yes	yes	no	yes	yes
	26	no	no	no	no	no	no	no	no
	27	no	no	no	yes	no	no	yes	no

	28	no	no	yes	no	no	no	yes	no
	29	no	no	no	yes	yes	no	yes	yes
	Total N	29	29	29	29	29	29	29	29
2	1	no	no	no	no	no	no	no	no
	2	no	no	no	no	no	no	no	no
	3	no	no	no	no	no	no	no	no
	4	no	no	no	no	no	no	no	no
	5	no	no	no	no	no	no	no	no
	6	no	no	no	yes	yes	yes	no	no
	7	no	no	no	no	yes	no	no	no
	8	no	no	no	no	yes	no	no	no
	9	no	no	yes	no	yes	no	no	no
	10	no	no	no	no	yes	no	no	yes
	11	yes	no	yes	no	no	no	yes	no
	12	yes	yes	no	no	no	no	no	no
	13	no	no	no	no	no	no	no	no
	14	yes	no	no	no	no	no	no	no
	15	yes	no	no	no	no	no	no	no
	16	no	yes	no	no	no	no	no	no
	17	no	no	no	no	no	no	yes	no
	18	no	yes	no	no	no	no	no	no
	19	no	no	no	no	no	no	no	no
	20	no	no	no	no	no	no	no	no
	21	no	no	no	no	no	no	no	no
	22	no	no	no	yes	yes	no	yes	no
	23	no	no	no	yes	yes	no	yes	no
	24	no	yes	no	no	no	no	no	no
	25	yes	yes	no	no	no	no	no	no
	26	yes	yes	no	no	no	no	no	no
	27	yes	no	no	no	no	yes	no	no
	28	no	no	no	no	no	yes	no	no
	29	no	no	no	no	no	yes	no	no
	30	no	no	no	no	no	no	yes	no
	31	yes	no	no	no	no	no	no	yes
	32	yes	yes	yes	no	no	yes	yes	no
	33	yes	yes	yes	no	no	yes	yes	no
	34	no	no	no	no	yes	yes	no	no
	35	no	no	no	no	no	no	no	no
	36	no	no	no	no	no	no	no	no
	37	yes	no	no	yes	yes	no	no	no

	38	yes	no	no	yes	yes	yes	yes	no
	39	no	no	yes	yes	yes	yes	yes	no
	40	yes	no	yes	no	yes	yes	no	no
	41	yes	no	no	yes	yes	yes	yes	no
	42	no	no	yes	yes	yes	yes	yes	no
	43	no	no	no	no	yes	yes	yes	yes
	44	no	no	no	no	no	yes	yes	no
	45	no	no	no	no	yes	yes	yes	yes
	46	no	no	yes	no	no	no	no	no
	47	no	no	no	yes	no	no	no	no
	48	no	no	no	yes	yes	yes	yes	yes
	49	no	no	no	no	yes	no	yes	yes
	50	yes	no	no	no	no	no	yes	no
	51	yes	no	no	yes	yes	yes	no	yes
	52	no	no	yes	yes	yes	no	yes	no
	53	no	no	no	no	no	no	yes	no
	54	no	no	no	no	no	yes	yes	yes
	55	no	no	no	yes	yes	no	yes	no
	56	no	no	no	no	no	no	no	no
	57	no	no	no	no	yes	yes	yes	no
	58	yes	no	no	yes	no	no	no	no
	59	no	no	no	no	yes	yes	yes	no
	60	no	yes	no	no	yes	no	yes	yes
	61	no	yes	no	no	yes	no	yes	no
	62	no	no	yes	yes	yes	yes	no	no
	63	no	no	no	yes	yes	no	yes	no
	64	yes	no	no	yes	yes	no	yes	yes
	65	no	no	yes	no	yes	yes	no	no
	66	no	no	no	yes	no	no	yes	no
	67	yes	no	no	no	yes	yes	yes	no
	68	yes	no	no	no	yes	no	yes	no
	69	yes	no	no	no	no	no	yes	no
	Total N	69	69	69	69	69	69	69	69
3	1	yes	yes	no	yes	no	yes	no	no
	2	yes	yes	no	yes	no	yes	yes	no
	3	yes	yes	no	yes	no	yes	yes	no
	4	yes	yes	no	yes	no	yes	yes	no
	5	yes	yes	no	yes	no	yes	yes	no
	6	no	yes	no	no	no	yes	yes	no
	7	no	no	no	no	yes	yes	no	no

8	no	no	no	yes	yes	yes	no	yes
9	yes	no	no	no	no	yes	no	no
10	yes	yes	no	no	yes	yes	no	no
11	yes	yes	no	no	no	yes	no	no
12	no	no	no	no	no	yes	no	no
13	yes	yes	no	no	no	no	yes	no
14	yes	yes	no	yes	no	no	yes	no
15	no	yes	no	no	no	no	no	no
16	no	no	no	no	no	no	yes	no
17	no	no	no	no	no	no	yes	no
18	no	no	no	no	no	yes	yes	no
19	no	no	no	no	no	yes	no	no
20	yes	no	no	no	no	yes	no	no
21	yes	yes	no	no	no	yes	no	no
22	yes	yes	no	no	no	no	no	no
23	yes	no	no	no	no	yes	yes	no
24	yes	no	yes	no	no	no	no	no
25	yes	no	no	no	no	yes	yes	no
26	yes	no	no	no	no	no	yes	no
27	yes	no	no	no	no	no	yes	no
28	yes	no	yes	no	no	yes	yes	no
29	yes	no	no	yes	yes	yes	no	no
30	yes	no	no	yes	yes	yes	no	no
31	no	yes	no	no	no	no	no	no
32	no	yes	no	no	no	no	no	no
33	yes	yes	no	no	yes	yes	no	no
34	yes	no	no	no	no	yes	no	no
35	no	no	no	yes	yes	yes	yes	no
36	yes	no	yes	yes	yes	yes	yes	no
37	yes	no	no	no	no	yes	no	no
38	yes	yes	no	no	no	no	no	no
39	yes	no	no	no	no	yes	no	no
40	yes	yes	no	no	no	no	yes	no
41	yes	no	no	no	no	yes	no	no
42	yes	no	no	no	no	no	no	no
43	no	yes	no	no	no	no	no	no
44	no	no	no	no	no	yes	no	no
45	yes	no	no	no	yes	yes	no	no
46	no	no	no	no	no	no	yes	no
47	no	no	no	no	no	yes	yes	no
48	yes	yes	no	no	no	no	no	no

49	yes	yes	no	no	no	no	no	no
50	yes	no	no	no	no	no	yes	no
51	yes	yes	no	no	no	yes	no	no
52	yes	yes	yes	no	no	no	yes	no
53	yes	yes	no	yes	no	yes	yes	no
54	yes	yes	no	no	no	no	no	yes
55	yes	no	yes	no	no	yes	yes	no
56	no	yes	no	no	no	no	no	no
57	yes	no	yes	no	no	no	yes	no
58	yes	no	no	no	no	no	no	no
59	yes	no	no	yes	no	yes	no	yes
60	yes	no	no	yes	yes	yes	yes	no
61	yes	no	yes	no	no	no	yes	no
62	yes	no	no	no	no	yes	no	no
63	yes	no	yes	no	no	yes	no	no
64	yes	no	yes	yes	no	yes	no	no
65	yes	yes	no	no	no	yes	no	no
66	yes	no	no	no	no	yes	yes	no
67	yes	no	no	no	no	yes	yes	yes
68	yes	no	no	no	yes	yes	yes	no
69	yes	no	no	no	yes	yes	yes	no
70	yes	no	no	yes	yes	yes	yes	yes
71	yes	no	no	no	no	yes	no	no
72	yes	no	no	yes	no	yes	no	no
73	no	no	yes	no	no	yes	no	no
74	yes	no	yes	no	yes	yes	no	yes
75	yes	no	no	no	no	yes	yes	no
76	yes	no	no	no	yes	yes	no	no
77	yes	no	no	no	no	yes	no	no
78	yes	no	no	yes	yes	yes	yes	yes
79	yes	no	no	no	no	yes	yes	no
80	no	yes	no	yes	yes	yes	yes	yes
81	no	no	no	yes	no	yes	yes	no
82	yes	no	yes	no	yes	yes	yes	yes
83	yes	no	no	no	yes	yes	yes	no
84	yes	yes	no	yes	yes	yes	yes	yes
85	yes	no	no	no	yes	yes	no	yes
86	yes	no	no	no	no	yes	no	no
87	yes	no	yes	yes	yes	yes	yes	no
88	no	no	no	yes	no	yes	yes	no
89	yes	no	yes	no	yes	yes	yes	no

	90	yes	no	yes	yes	no	no	yes	no
	91	yes	yes	yes	yes	yes	yes	yes	no
	92	yes	no	no	no	yes	yes	yes	no
	93	yes	no	no	no	yes	yes	yes	no
	94	yes	no	no	no	no	yes	yes	no
	95	no	yes	no	no	yes	yes	yes	no
	96	yes	no	no	no	no	yes	yes	no
	97	yes	no	no	yes	yes	yes	yes	yes
	98	no	yes	no	no	no	yes	no	no
	99	yes	yes	no	no	yes	yes	no	no
	100	no	yes	no	yes	yes	yes	yes	no
	101	no	no	no	yes	yes	yes	no	yes
	102	yes	no	no	no	no	yes	yes	no
	103	yes	no	no	no	no	yes	no	no
	104	yes	yes	no	no	no	yes	yes	no
	105	no	no	no	yes	yes	yes	yes	yes
	106	yes	no	no	no	yes	yes	yes	no
	Total N	106	106	106	106	106	106	106	106
4	1	yes	yes	yes	no	yes	yes	no	no
	2	yes	yes	no	no	no	yes	no	no
	3	no	yes	no	no	no	yes	no	no
	4	no	yes	no	no	no	yes	no	no
	5	yes	yes	no	no	yes	yes	no	no
	6	yes	yes	no	no	yes	yes	no	no
	7	yes	yes	no	no	yes	yes	yes	no
	8	no	yes	yes	no	yes	yes	yes	yes
	9	yes	yes	no	no	no	yes	no	no
	10	yes	yes	no	no	no	yes	no	no
	11	yes	no	no	no	no	yes	yes	no
	12	yes	no	no	no	no	yes	no	no
	13	yes	no	no	no	no	yes	no	no
	14	yes	no	no	no	no	yes	no	no
	15	yes	yes	no	no	no	yes	no	no
	16	yes	yes	no	no	yes	yes	no	no
	17	yes	yes	no	no	no	yes	no	no
	18	yes	yes	no	no	no	yes	no	no
	19	yes	yes	no	no	no	yes	no	no
	20	yes	yes	no	no	no	yes	no	no
	21	yes	yes	no	no	yes	yes	yes	no
	22	yes	yes	yes	no	no	yes	no	no

23	yes	yes	no	no	no	yes	no	no
24	no	no	no	no	no	yes	no	no
25	no	no	no	no	no	yes	no	no
26	yes	yes	no	no	no	no	no	no
27	yes	yes	no	yes	no	yes	yes	no
28	yes	no	no	no	no	yes	no	no
29	yes	no	no	no	no	yes	no	no
30	yes	no	no	yes	yes	yes	no	no
31	yes	yes	no	yes	yes	yes	no	no
32	yes	yes	no	no	no	yes	no	no
33	yes	yes	yes	no	yes	yes	no	no
34	yes	no	no	no	yes	yes	no	no
35	yes	yes	no	no	no	yes	yes	no
36	yes	yes	no	yes	no	yes	no	no
37	yes	yes	no	no	yes	yes	no	no
38	yes	yes	no	yes	no	yes	no	no
39	yes	yes	yes	no	yes	yes	no	no
40	yes	yes	no	no	no	yes	yes	no
41	yes	yes	no	no	no	yes	yes	no
42	yes	yes	no	no	no	yes	no	no
43	yes	yes	yes	no	no	yes	no	no
44	no	yes	no	yes	yes	yes	yes	no
45	yes	no	no	no	no	yes	no	no
46	yes	yes	no	yes	yes	yes	yes	yes
47	yes	yes	no	no	no	yes	no	no
48	yes	no	no	yes	no	yes	yes	no
49	yes	no	yes	no	no	yes	no	no
50	no	no	no	no	no	yes	no	no
51	yes	no	no	yes	yes	yes	yes	no
52	yes	no	no	yes	yes	yes	yes	yes
53	yes	no	yes	no	no	yes	yes	yes
54	yes	no	no	no	no	yes	no	no
55	yes	yes	yes	no	yes	yes	yes	yes
56	yes	no	yes	yes	yes	yes	yes	no
57	yes	no	no	no	no	yes	yes	no
58	yes	no	no	no	yes	yes	yes	yes
59	yes	no	no	no	yes	yes	yes	no
60	yes	no	no	no	yes	yes	yes	no
61	yes	no	no	yes	yes	yes	no	no
62	yes	no	no	no	yes	yes	yes	yes
63	yes	no	no	no	no	yes	no	no

	64	yes	no	no	yes	yes	yes	yes	yes
	65	yes	no	no	no	no	yes	yes	no
	66	yes	yes	yes	no	no	yes	no	no
	67	no	yes	no	yes	yes	yes	yes	no
	68	yes	yes	yes	yes	yes	yes	yes	no
	69	yes	yes	no	no	no	yes	yes	no
	70	yes	yes	no	no	yes	yes	no	yes
	71	yes	yes	no	no	no	yes	yes	no
	72	yes	yes	no	no	no	yes	no	no
	73	yes	yes	no	no	no	yes	no	no
	74	yes	yes	yes	yes	yes	yes	yes	no
	75	yes	no	yes	no	no	yes	no	yes
	76	no	no	no	yes	yes	yes	yes	yes
	77	yes	no	no	no	yes	yes	yes	yes
	78	yes	no	no	no	no	yes	no	no
	79	yes	yes	yes	no	yes	yes	yes	yes
	80	yes	yes	no	yes	yes	yes	yes	no
	81	yes	yes	yes	no	no	yes	yes	no
	Total N	81	81	81	81	81	81	81	81
Total N		285	285	285	285	285	285	285	285

a. Limited to first 300 cases.

SUPPLEMENTARY MATERIAL 3

Table S1: Case summaries for comorbidities

Case Summaries[a]

		Respiratory failure	Other_pulmonary_diseases	Pulmonary_embolism	Heart_disease	Cardiac_failure	Effort_related_fatigue	Diabetes	Atrial_fibrilation_flutter
GOLD1	1	no	no	no	no	no	no	yes	no
	2	no	no	no	no	no	no	yes	no
	3	no	no	no	no	no	no	no	no
	4	no	no	no	no	no	no	no	no
	5	yes	yes	no	no	no	no	yes	no
	6	yes	yes	no	yes	no	no	yes	no
	7	no	yes	yes	no	no	no	no	no
	8	no	no	no	no	no	no	yes	no
	9	no	no	no	no	no	no	yes	no
	10	no	no	no	no	yes	no	yes	no
	11	no	no	yes	no	yes	no	no	yes
	12	no	no	no	yes	no	no	yes	no
	13	no	no	no	no	yes	no	yes	no
	14	no	no	no	yes	yes	no	no	no
	15	no	no	yes	no	yes	no	no	no
	16	no	no	yes	no	yes	no	yes	yes
	17	no	no	no	yes	yes	no	yes	yes
	18	no	no	no	yes	yes	no	yes	no
	19	no	no	no	no	yes	no	yes	no

	20	no	no	no	no	yes	no	yes	no
	21	no	no	no	no	yes	no	yes	no
	22	no	no	yes	no	yes	no	yes	yes
	23	no	no	no	no	yes	no	yes	no
	24	no	yes	no	yes	yes	no	yes	no
	25	no	no	no	yes	yes	no	yes	yes
	26	no	no	no	no	no	no	no	no
	27	no	no	no	yes	no	no	yes	no
	28	no	no	yes	no	no	no	yes	no
	29	no	no	no	yes	yes	no	yes	yes
	Total N	29	29	29	29	29	29	29	29
2	1	no	no	no	no	no	no	no	no
	2	no	no	no	no	no	no	no	no
	3	no	no	no	no	no	no	no	no
	4	no	no	no	no	no	no	no	no
	5	no	no	no	no	no	no	no	no
	6	no	no	no	yes	yes	yes	no	no
	7	no	no	no	no	yes	no	no	no
	8	no	no	no	no	yes	no	no	no
	9	no	no	yes	no	yes	no	no	no
	10	no	no	no	no	yes	no	no	yes
	11	yes	no	yes	no	no	no	yes	no
	12	yes	yes	no	no	no	no	no	no
	13	no	no	no	no	no	no	no	no
	14	yes	no	no	no	no	no	no	no
	15	yes	no	no	no	no	no	no	no
	16	no	yes	no	no	no	no	no	no
	17	no	no	no	no	no	no	yes	no
	18	no	yes	no	no	no	no	no	no

19	no	no	no	no	no	no	no	no
20	no	no	no	no	no	no	no	no
21	no	no	no	no	no	no	no	no
22	no	no	no	yes	yes	no	yes	no
23	no	no	no	yes	yes	no	yes	no
24	no	yes	no	no	no	no	no	no
25	yes	yes	no	no	no	no	no	no
26	yes	yes	no	no	no	no	no	no
27	yes	no	no	no	no	yes	no	no
28	no	no	no	no	no	yes	no	no
29	no	no	no	no	no	yes	no	no
30	no	no	no	no	no	no	yes	no
31	yes	no	no	no	no	no	no	yes
32	yes	yes	yes	no	no	yes	yes	no
33	yes	yes	yes	no	no	yes	yes	no
34	no	no	no	no	yes	yes	no	no
35	no	no	no	no	no	no	no	no
36	no	no	no	no	no	no	no	no
37	yes	no	no	yes	yes	no	no	no
38	yes	no	no	yes	yes	yes	yes	no
39	no	no	yes	yes	yes	yes	yes	no
40	yes	no	yes	no	yes	yes	no	no
41	yes	no	no	yes	yes	yes	yes	no
42	no	no	yes	yes	yes	yes	yes	no
43	no	no	no	no	yes	yes	yes	yes
44	no	no	no	no	no	yes	yes	no
45	no	no	no	no	yes	yes	yes	yes
46	no	no	yes	no	no	no	no	no
47	no	no	no	yes	no	no	no	no

	48	no	no	no	yes	yes	yes	yes	yes
	49	no	no	no	no	yes	no	yes	yes
	50	yes	no	no	no	no	no	yes	no
	51	yes	no	no	yes	yes	yes	no	yes
	52	no	no	yes	yes	yes	no	yes	no
	53	no	no	no	no	no	no	yes	no
	54	no	no	no	no	no	yes	yes	yes
	55	no	no	no	yes	yes	no	yes	no
	56	no	no	no	no	no	no	no	no
	57	no	no	no	no	yes	yes	yes	no
	58	yes	no	no	yes	no	no	no	no
	59	no	no	no	no	yes	yes	yes	no
	60	no	yes	no	no	yes	no	yes	yes
	61	no	yes	no	no	yes	no	yes	no
	62	no	no	yes	yes	yes	yes	no	no
	63	no	no	no	yes	yes	no	yes	no
	64	yes	no	no	yes	yes	no	yes	yes
	65	no	no	yes	no	yes	yes	no	no
	66	no	no	no	yes	no	no	yes	no
	67	yes	no	no	no	yes	yes	yes	no
	68	yes	no	no	no	yes	no	yes	no
	69	yes	no	no	no	no	no	yes	no
	Total N	69	69	69	69	69	69	69	69
3	1	yes	yes	no	yes	no	yes	no	no
	2	yes	yes	no	yes	no	yes	yes	no
	3	yes	yes	no	yes	no	yes	yes	no
	4	yes	yes	no	yes	no	yes	yes	no
	5	yes	yes	no	yes	no	yes	yes	no
	6	no	yes	no	no	no	yes	yes	no

7	no	no	no	no	yes	yes	no	no
8	no	no	no	yes	yes	yes	no	yes
9	yes	no	no	no	no	yes	no	no
10	yes	yes	no	no	yes	yes	no	no
11	yes	yes	no	no	no	yes	no	no
12	no	no	no	no	no	yes	no	no
13	yes	yes	no	no	no	no	yes	no
14	yes	yes	no	yes	no	no	yes	no
15	no	yes	no	no	no	no	no	no
16	no	no	no	no	no	no	yes	no
17	no	no	no	no	no	no	yes	no
18	no	no	no	no	no	yes	yes	no
19	no	no	no	no	no	yes	no	no
20	yes	no	no	no	no	yes	no	no
21	yes	yes	no	no	no	yes	no	no
22	yes	yes	no	no	no	no	no	no
23	yes	no	no	no	no	yes	yes	no
24	yes	no	yes	no	no	no	no	no
25	yes	no	no	no	no	yes	yes	no
26	yes	no	no	no	no	no	yes	no
27	yes	no	no	no	no	no	yes	no
28	yes	no	yes	no	no	yes	yes	no
29	yes	no	no	yes	yes	yes	no	no
30	yes	no	no	yes	yes	yes	no	no
31	no	yes	no	no	no	no	no	no
32	no	yes	no	no	no	no	no	no
33	yes	yes	no	no	yes	yes	no	no
34	yes	no	no	no	no	yes	no	no
35	no	no	no	yes	yes	yes	yes	no

36	yes	no	yes	yes	yes	yes	yes	no
37	yes	no	no	no	no	yes	no	no
38	yes	yes	no	no	no	no	no	no
39	yes	no	no	no	no	yes	no	no
40	yes	yes	no	no	no	no	yes	no
41	yes	no	no	no	no	yes	no	no
42	yes	no	no	no	no	no	no	no
43	no	yes	no	no	no	no	no	no
44	no	no	no	no	no	yes	no	no
45	yes	no	no	no	yes	yes	no	no
46	no	no	no	no	no	no	yes	no
47	no	no	no	no	no	yes	yes	no
48	yes	yes	no	no	no	no	no	no
49	yes	yes	no	no	no	no	no	no
50	yes	no	no	no	no	no	yes	no
51	yes	yes	no	no	no	yes	no	no
52	yes	yes	yes	no	no	no	yes	no
53	yes	yes	no	yes	no	yes	yes	no
54	yes	yes	no	no	no	no	no	yes
55	yes	no	yes	no	no	yes	yes	no
56	no	yes	no	no	no	no	no	no
57	yes	no	yes	no	no	no	yes	no
58	yes	no	no	no	no	no	no	no
59	yes	no	no	yes	no	yes	no	yes
60	yes	no	no	yes	yes	yes	yes	no
61	yes	no	yes	no	no	no	yes	no
62	yes	no	no	no	no	yes	no	no
63	yes	no	yes	no	no	yes	no	no
64	yes	no	yes	yes	no	yes	no	no

65	yes	yes	no	no	no	yes	no	no
66	yes	no	no	no	no	yes	yes	no
67	yes	no	no	no	no	yes	yes	yes
68	yes	no	no	no	yes	yes	yes	no
69	yes	no	no	no	yes	yes	yes	no
70	yes	no	no	yes	yes	yes	yes	yes
71	yes	no	no	no	no	yes	no	no
72	yes	no	no	yes	no	yes	no	no
73	no	no	yes	no	no	yes	no	no
74	yes	no	yes	no	yes	yes	no	yes
75	yes	no	no	no	no	yes	yes	no
76	yes	no	no	no	yes	yes	no	no
77	yes	no	no	no	no	yes	no	no
78	yes	no	no	yes	yes	yes	yes	yes
79	yes	no	no	no	no	yes	yes	no
80	no	yes	no	yes	yes	yes	yes	yes
81	no	no	no	yes	no	yes	yes	no
82	yes	no	yes	no	yes	yes	yes	yes
83	yes	no	no	no	yes	yes	yes	no
84	yes	yes	no	yes	yes	yes	yes	yes
85	yes	no	no	no	yes	yes	no	yes
86	yes	no	no	no	no	yes	no	no
87	yes	no	yes	yes	yes	yes	yes	no
88	no	no	no	yes	no	yes	yes	no
89	yes	no	yes	no	yes	yes	yes	no
90	yes	no	yes	yes	no	no	yes	no
91	yes	yes	yes	yes	yes	yes	yes	no
92	yes	no	no	no	yes	yes	yes	no
93	yes	no	no	no	yes	yes	yes	no

	94	yes	no	no	no	no	yes	yes	no
	95	no	yes	no	no	yes	yes	yes	no
	96	yes	no	no	no	no	yes	yes	no
	97	yes	no	no	yes	yes	yes	yes	yes
	98	no	yes	no	no	no	yes	no	no
	99	yes	yes	no	no	yes	yes	no	no
	100	no	yes	no	yes	yes	yes	yes	no
	101	no	no	no	yes	yes	yes	no	yes
	102	yes	no	no	no	no	yes	yes	no
	103	yes	no	no	no	no	yes	no	no
	104	yes	yes	no	no	no	yes	yes	no
	105	no	no	no	yes	yes	yes	yes	yes
	106	yes	no	no	no	yes	yes	yes	no
	Total N	106	106	106	106	106	106	106	106
4	1	yes	yes	yes	no	yes	yes	no	no
	2	yes	yes	no	no	no	yes	no	no
	3	no	yes	no	no	no	yes	no	no
	4	no	yes	no	no	no	yes	no	no
	5	yes	yes	no	no	yes	yes	no	no
	6	yes	yes	no	no	yes	yes	no	no
	7	yes	yes	no	no	yes	yes	yes	no
	8	no	yes	yes	no	yes	yes	yes	yes
	9	yes	yes	no	no	no	yes	no	no
	10	yes	yes	no	no	no	yes	no	no
	11	yes	no	no	no	no	yes	yes	no
	12	yes	no	no	no	no	yes	no	no
	13	yes	no	no	no	no	yes	no	no
	14	yes	no	no	no	no	yes	no	no
	15	yes	yes	no	no	no	yes	no	no

16	yes	yes	no	no	yes	yes	no	no
17	yes	yes	no	no	no	yes	no	no
18	yes	yes	no	no	no	yes	no	no
19	yes	yes	no	no	no	yes	no	no
20	yes	yes	no	no	no	yes	no	no
21	yes	yes	no	no	yes	yes	yes	no
22	yes	yes	yes	no	no	yes	no	no
23	yes	yes	no	no	no	yes	no	no
24	no	no	no	no	no	yes	no	no
25	no	no	no	no	no	yes	no	no
26	yes	yes	no	no	no	no	no	no
27	yes	yes	no	yes	no	yes	yes	no
28	yes	no	no	no	no	yes	no	no
29	yes	no	no	no	no	yes	no	no
30	yes	no	no	yes	yes	yes	no	no
31	yes	yes	no	yes	yes	yes	no	no
32	yes	yes	no	no	no	yes	no	no
33	yes	yes	yes	no	yes	yes	no	no
34	yes	no	no	no	yes	yes	no	no
35	yes	yes	no	no	no	yes	yes	no
36	yes	yes	no	yes	no	yes	no	no
37	yes	yes	no	no	yes	yes	no	no
38	yes	yes	no	yes	no	yes	no	no
39	yes	yes	yes	no	yes	yes	no	no
40	yes	yes	no	no	no	yes	yes	no
41	yes	yes	no	no	no	yes	yes	no
42	yes	yes	no	no	no	yes	no	no
43	yes	yes	yes	no	no	yes	no	no
44	no	yes	no	yes	yes	yes	yes	no

45	yes	no	no	no	no	yes	no	no
46	yes	yes	no	yes	yes	yes	yes	yes
47	yes	yes	no	no	no	yes	no	no
48	yes	no	no	yes	no	yes	yes	no
49	yes	no	yes	no	no	yes	no	no
50	no	no	no	no	no	yes	no	no
51	yes	no	no	yes	yes	yes	yes	no
52	yes	no	no	yes	yes	yes	yes	yes
53	yes	no	yes	no	no	yes	yes	yes
54	yes	no	no	no	no	yes	no	no
55	yes	yes	yes	no	yes	yes	yes	yes
56	yes	no	yes	yes	yes	yes	yes	no
57	yes	no	no	no	no	yes	yes	no
58	yes	no	no	no	yes	yes	yes	yes
59	yes	no	no	no	yes	yes	yes	no
60	yes	no	no	no	yes	yes	yes	no
61	yes	no	no	yes	yes	yes	no	no
62	yes	no	no	no	yes	yes	yes	yes
63	yes	no	no	no	no	yes	no	no
64	yes	no	no	yes	yes	yes	yes	yes
65	yes	no	no	no	no	yes	yes	no
66	yes	yes	yes	no	no	yes	no	no
67	no	yes	no	yes	yes	yes	yes	no
68	yes	yes	yes	yes	yes	yes	yes	no
69	yes	yes	no	no	no	yes	yes	no
70	yes	yes	no	no	yes	yes	no	yes
71	yes	yes	no	no	no	yes	yes	no
72	yes	yes	no	no	no	yes	no	no
73	yes	yes	no	no	no	yes	no	no

		74	yes	yes	yes	yes	yes	yes	yes	no
		75	yes	no	yes	no	no	yes	no	yes
		76	no	no	no	yes	yes	yes	yes	yes
		77	yes	no	no	no	yes	yes	yes	yes
		78	yes	no	no	no	no	yes	no	no
		79	yes	yes	yes	no	yes	yes	yes	yes
		80	yes	yes	no	yes	yes	yes	yes	no
		81	yes	yes	yes	no	no	yes	yes	no
		Total N	81	81	81	81	81	81	81	81
	Total N		285	285	285	285	285	285	285	285

a. Limited to first 300 cases.

Table S2: Case summaries for treatment

Case Summaries[a]

			Ceftriaxone	Meropenem	Cefuroxime	Moxifloxacin	Metoprolol	Salbutamol	Amlodipine	Acetylcysteine	Hydrocortisone	Dexamethasone	Furosemide	Aminophylline	Omeprazole	Pantoprazole	Ascorbic_acid	Calcium_gluconate	Heparin	Apixaban	Enoxaparin	Atorvastatin	Nitroglycerin
GOLD	1	1	yes	no	no	no	no	yes	no	yes	yes	no	no	no	yes	no	no	no	no	no	no	no	no
		2	no	no	no	no	no	yes	no	no	no	no	no	no	no	no	no	no	no	no	no	no	no
		3	yes	no	no	no	no	yes	no	yes	yes	no	no	no	yes	no	no	no	no	no	no	no	no
		4	yes	no	no	no	no	no	no	no	no	no	no	no	no	no	no	no	no	no	no	no	no
		5	no	no	no	no	no	no	no	no	no	no	no	no	no	no	no	no	no	no	no	no	no

6	yes	no	no	no	no	no	yes	no	yes	yes	yes	no	yes	no	no	no	no	no	no	yes	no
7	no	no	no	no	no	no	no	no	no	no	no	no	no	no	no	no	no	no	no	no	no
8	yes	no	no	no	no	no	yes	yes	no	no	yes	no	yes	no	no	no	no	no	no	no	no
9	no	no	no	no	no	yes	no	no	no	no	no	no	no	no	no	no	no	no	no	no	no
10	no	no	no	no	no	no	no	no	no	no	no	no	no	no	no	no	no	no	no	no	no
11	yes	no	no	yes	yes	no	no	yes	no	yes	no	no	yes	no	no	no	no	yes	no	yes	no
12	no	no	no	no	no	no	no	yes	no	yes	yes	no	no	no	no	no	no	no	yes	no	no
13	yes	no	no	no	no	no	no	no	no	no	no	no	no	no	no	no	no	no	no	no	no
14	yes	no	no	no	no	yes	yes	yes	no	yes	yes	no	yes	no	no	no	no	no	no	no	no
15	no	no	no	yes	no	no	yes	no	no	yes	yes	no	no	no	no	no	no	no	no	no	yes
16	no	no	no	no	no	no	yes	no	no	yes	no	no	no	no	no	no	no	no	no	no	yes
17	no	no	no	no	no	no	no	yes	yes	yes	no	no	yes	no	no	no	yes	no	no	no	no
18	yes	no	no	no	no	yes	yes	no	yes	no	no	no	yes	no	no	no	no	no	no	no	no
19	yes	no	yes	yes	no	no	yes	yes	no	yes	yes	yes	no	no	no	no	no	no	no	yes	no
20	no	no	no	no	no	no	yes	yes	no	yes	yes	yes	yes	no	no	no	no	no	no	no	no
21	yes	no	no	yes	yes	yes	yes	no	no	yes	no	no	yes	no	no	no	no	no	yes	yes	no
22	no	no	no	no	no	no	yes	yes	yes	yes	no	no	yes	no	no	no	no	no	no	yes	no
23	yes	no	no	no	no	no	yes	yes	no	no	yes	no	no	no	no	no	yes	no	no	no	no
24	yes	no	no	yes	no	yes	yes	yes	yes	yes	no	yes	yes	no	no	no	no	no	yes	no	no
25	no	no	no	yes	yes	no	no	no	no	yes	yes	no	yes	no	no	no	yes	no	yes	yes	no
26	no	no	yes	no	yes	no	yes	yes	no	yes	yes	no	yes	yes	yes	yes	no	yes	no	yes	no

	27	yes	no	no	no	yes	yes	yes	yes	no	yes	yes	no	yes	no	no	no	no	yes	no	yes	no
	28	yes	no	no	no	yes	yes	yes	yes	yes	yes	no	no	yes	no	no	yes	no	yes	no	no	no
	29	yes	no	no	no	no	yes	yes	yes	yes	yes	no	yes	no	no	yes	no	no	no	no	no	yes
	Total N	29	29	29	29	29	29	29	29	29	29	29	29	29	29	29	29	29	29	29	29	29
2	1	yes	no	no	no	no	yes	no	no	yes	no	no	no	yes	no	no	no	no	no	no	no	no
	2	yes	no	no	no	no	yes	no	no	yes	no	no	no	yes	no	no	no	no	no	no	no	no
	3	yes	no	no	no	no	no	no	no	yes	no	no	no	no	no	no	no	no	no	no	no	no
	4	yes	no	no	no	no	no	no	no	yes	no	no	no	yes	no	no	no	no	no	no	no	no
	5	yes	no	no	no	no	no	no	no	yes	no	no	no	yes	no	no	no	no	no	no	no	no
	6	no	no	no	no	no	no	no	no	no	no	no	no	no	no	no	no	no	no	no	no	no
	7	yes	no	no	no	no	no	no	no	yes	no	no	no	yes	no	no	no	no	no	no	no	no
	8	yes	no	no	no	no	no	no	no	yes	no	no	no	yes	no	no	no	no	no	no	no	no
	9	no	no	no	no	no	no	no	no	no	no	no	no	yes	no	no	no	no	no	no	no	no
	10	no	no	no	no	no	no	no	no	no	no	no	no	no	no	no	no	no	no	no	no	no
	11	yes	no	no	no	no	no	no	no	no	no	no	no	no	no	no	no	no	no	no	no	no
	12	yes	no	no	no	no	yes	no	yes	yes	no	no	no	yes	no	no	no	no	no	no	no	no
	13	yes	no	no	no	no	no	no	no	no	no	no	no	no	no	no	no	no	no	no	no	no
	14	yes	no	no	no	no	no	no	no	no	no	no	no	no	no	no	no	no	no	no	no	no
	15	yes	no	no	no	no	no	no	no	no	no	no	no	no	no	no	no	no	no	no	no	no
	16	yes	no	no	no	no	yes	no	yes	yes	no	no	no	yes	no	no	no	yes	no	no	no	no

17	yes	no	no	no	no	no	no	no	no	no	no	no	no	no	no	no	no	no	no	no	no
18	yes	no	no	no	no	no	no	no	no	no	no	no	yes	no	no	no	no	no	no	no	no
19	yes	no	no	no	no	no	no	no	yes	no	no	no	no	no	no	no	no	no	no	no	yes
20	yes	no	no	no	no	no	no	yes	yes	yes	no	no	yes	no	no	no	no	no	no	no	no
21	no	no	no	no	no	no	no	no	no	no	no	no	no	no	no	no	no	no	no	no	no
22	no	no	no	no	no	no	no	no	no	no	no	no	no	no	no	no	no	no	no	no	no
23	yes	no	no	no	no	yes	no	no	no	no	no	no	yes	no	no	no	no	no	no	no	no
24	no	no	no	no	no	no	no	no	no	no	no	no	no	no	no	no	no	no	no	no	no
25	yes	no	no	no	no	no	no	no	no	no	no	no	no	no	no	no	no	no	no	no	no
26	yes	no	no	no	no	no	no	no	no	no	no	no	no	no	no	no	no	no	no	no	no
27	yes	no	no	no	no	no	no	no	no	no	no	no	no	no	no	no	no	no	no	no	no
28	yes	no	no	no	no	no	no	no	no	no	no	no	no	no	no	no	no	no	no	no	no
29	yes	no	no	no	no	no	no	yes	yes	no	no	no	yes	no	no	no	no	no	no	no	no
30	no	no	no	no	no	yes	no	yes	no	no	no	no	yes	no	no	no	no	no	no	no	no
31	no	no	no	no	no	no	no	no	no	no	no	no	no	no	no	no	no	no	no	no	no
32	yes	no	no	no	no	no	no	yes	yes	yes	yes	no	yes	no	no	no	no	no	no	no	no
33	yes	no	no	no	no	no	no	yes	no	no	no	no	no	no	no	no	no	no	no	no	no
34	yes	no	no	no	no	no	yes	yes	yes	yes	yes	no	yes	yes	no	no	no	no	no	no	no
35	no	no	no	no	no	no	no	no	no	no	no	no	no	no	no	no	no	no	no	no	no
36	no	no	no	no	no	no	no	no	no	no	no	no	no	no	no	no	no	no	no	no	yes
37	yes	no	no	no	yes	yes	no	yes	yes	yes	yes	no	no	no	no	no	no	no	yes	no	no

38	yes	no	no	no	no	no	yes	yes	no	yes	yes	no	yes	no	no	no	no	no	no	no	no
39	no	yes	no	no	no	no	yes	no	yes	yes	no	no	yes	no	yes	no	no	no	no	no	no
40	no	no	no	no	no	no	no	no	no	no	no	no	no	no	no	no	no	no	no	no	no
41	no	yes	no	no	no	no	no	no	no	no	no	no	no	no	no	no	no	no	no	no	no
42	yes	no	no	no	no	no	yes	yes	no	yes	no	no	yes	no	no	no	no	no	no	no	no
43	no	no	no	no	yes	no	no	yes	no	yes	yes	no	yes	no	no	no	no	no	no	no	yes
44	no	no	no	no	no	no	no	no	no	no	no	no	no	no	no	no	no	no	no	no	no
45	no	no	no	no	no	no	no	no	no	no	no	yes	no	no	no	no	no	no	no	no	no
46	no	no	no	no	no	no	yes	yes	yes	no	no	no	yes	no	no	no	no	no	no	no	no
47	yes	no	no	no	no	yes	yes	yes	yes	yes	yes	no	yes	no	no	no	no	no	no	no	yes
48	no	no	no	no	no	no	yes	no	yes	no	yes	no	yes	no	no	yes	ye s	yes	no	no	no
49	yes	no	no	no	yes	no	yes	yes	no	yes	no	no	yes	no	yes	no	no	yes	no	no	no
50	no	no	no	no	no	no	no	yes	no	no	no	no	yes	no	no	no	no	no	no	no	yes
51	no	no	no	no	no	no	yes	yes	yes	yes	yes	yes	yes	no	no	no	no	yes	no	no	no
52	no	yes	no	no	no	no	yes	no	yes	yes	no	no	yes	no	yes	no	no	no	no	no	no
53	no	no	no	no	yes	no	no	yes	no	no	no	no	yes	no	no	no	no	no	no	no	no
54	no	no	no	yes	yes	no	yes	yes	yes	yes	yes	no	yes	yes	no	yes	no	yes	no	yes	no
55	yes	no	no	no	no	no	yes	yes	no	no	yes	yes	yes	no	no	no	no	no	no	no	yes
56	no	no	no	yes	no	no	no	yes	no	no	no	no	no	no	no	no	no	no	no	no	no
57	yes	no	no	no	no	no	yes	no	no	yes	no	no	yes	no	no	no	no	no	no	no	no
58	yes	no	no	yes	no	no	yes	yes	no	no	yes	no	yes	no	no	no	no	no	no	no	yes

	59	no	no	no	no	yes	no	yes	yes	no	yes	yes	yes	yes	no	no	no	no	yes	no	no	yes
	60	yes	no	yes	no	yes	no	yes	no	yes	yes	yes	no	yes	no	no	no	no	yes	yes	yes	no
	61	no	no	no	no	yes	no	yes	yes	yes	yes	yes	no	yes	no	no	no	no	yes	yes	no	no
	62	no	no	no	no	no	no	no	yes	no	yes	yes	no	yes	no	no	no	no	yes	no	no	yes
	63	yes	no	no	no	no	no	no	yes	yes	no	no	yes	no	yes	no	no	yes	no	no	no	no
	64	no	no	no	no	yes	no	yes	yes	yes	yes	yes	no	yes	no	yes	no	yes	yes	no	no	no
	65	yes	no	no	no	no	no	yes	yes	no	yes	no	yes	yes	no	no	no	no	no	no	no	no
	66	no	no	no	no	no	no	no	no	no	no	no	no	no	no	no	no	no	no	no	no	yes
	67	yes	no	no	yes	yes	yes	yes	yes	no	yes	yes	no	yes	no	yes	no	no	yes	yes	no	no
	68	no	no	no	no	yes	no	yes	yes	no	no	yes	yes	yes	no	no	no	no	yes	no	no	no
	69	yes	no	no	no	no	yes	yes	yes	yes	yes	yes	no	yes	no	no	no	no	no	no	yes	no
	Total N	69	69	69	69	69	69	69	69	69	69	69	69	69	69	69	69	69	69	69	69	69
3	1	yes	no	no	no	no	yes	no	no	yes	no	no	no	yes	no	no	no	no	no	no	no	no
	2	yes	no	no	no	no	yes	no	no	yes	no	yes	no	yes	no	no	no	no	no	no	no	no
	3	no	no	no	no	no	yes	no	no	yes	no	yes	no	yes	no	no	no	no	no	no	no	no
	4	no	no	no	no	no	no	no	yes	yes	no	yes	no	yes	no	no	no	no	no	no	no	no
	5	yes	no	no	no	no	yes	no	no	yes	yes	yes	no	yes	no	no	no	no	no	no	no	no
	6	no	no	no	no	no	yes	no	yes	yes	no	no	no	yes	no	no	no	no	no	no	no	no
	7	yes	no	no	no	no	no	no	yes	yes	no	no	no	yes	no	no	no	no	no	no	no	no
	8	yes	no	no	no	no	no	no	yes	yes	no	no	no	yes	no	no	no	no	no	no	no	no

9	yes	no	no	no	no	yes	no	no	yes	no	no	no	no	no	no	no	no	no	no	no	no
10	yes	no	no	no	no	no	no	no	yes	no	no	no	no	no	no	no	no	no	no	no	no
11	no	no	no	no	no	no	no	no	yes	no	no	no	no	no	no	no	no	no	no	no	no
12	yes	no	no	no	no	yes	no	yes	yes	no	no	no	no	no	no	no	no	no	no	no	no
13	yes	no	no	no	no	yes	no	yes	yes	no	no	no	no	no	no	no	no	no	no	no	no
14	yes	no	no	no	no	no	no	yes	yes	no	no	no	no	no	no	no	no	no	no	no	no
15	no	no	no	no	no	yes	no	no	yes	no	no	no	yes	no	no	no	no	no	no	no	no
16	no	no	no	no	no	no	no	yes	yes	no	no	no	yes	no	no	no	no	no	no	no	no
17	yes	no	no	no	no	no	no	no	no	no	no	no	no	no	no	no	no	no	no	no	no
18	yes	no	no	no	no	no	no	no	no	no	no	no	no	no	no	no	no	no	no	no	no
19	yes	no	no	no	no	no	no	no	no	no	no	no	no	no	no	no	no	no	no	no	no
20	yes	no	no	no	no	yes	no	no	no	no	yes	no	yes	no	no	no	no	no	no	no	no
21	yes	no	no	no	no	yes	no	no	no	yes	no	no	yes	no	no	no	no	no	no	no	no
22	yes	no	no	no	no	no	no	no	no	no	no	no	no	no	no	no	no	no	no	no	no
23	no	no	no	no	no	no	no	no	no	no	no	no	no	no	no	no	no	no	no	no	no
24	yes	no	no	no	no	yes	no	no	no	no	no	no	no	no	no	no	no	no	no	no	no
25	yes	no	no	no	no	yes	no	yes	yes	no	yes	no	no	no	no	no	no	no	no	no	no
26	yes	no	no	no	no	no	no	no	no	no	no	no	no	no	no	no	no	no	no	no	no
27	yes	no	no	no	no	no	no	no	no	no	no	no	no	no	no	no	no	no	no	no	no
28	yes	yes	no	no	no	yes	no	yes	no	yes	no	no	yes	no	no	no	yes	no	no	no	no
29	yes	no	no	no	no	no	no	no	no	no	no	no	no	no	no	no	no	no	no	no	no

30	yes	no	no	no	no	no	no	no	no	no	no	no	no	no	no	no	no	no	no	no	no
31	no	no	no	no	no	yes	no	yes	yes	no	no	no	no	no	no	no	no	no	no	no	no
32	yes	no	no	no	no	yes	no	yes	yes	yes	no	no	yes	no	no	no	yes	no	no	no	no
33	no	no	no	no	no	no	no	no	yes	no	no	no	yes	no	no	no	no	no	no	no	no
34	yes	no	no	no	no	no	no	no	no	yes	no	no	yes	no	no	no	no	no	no	no	no
35	yes	no	no	no	no	no	no	no	no	no	no	no	no	no	no	no	no	no	no	no	no
36	yes	no	no	no	no	no	no	no	no	no	no	no	no	no	no	no	no	no	no	no	no
37	yes	no	no	no	no	no	no	no	no	no	no	no	no	no	no	no	no	no	no	no	no
38	no	no	no	no	no	no	no	no	no	no	no	no	no	no	no	no	no	no	no	no	no
39	yes	no	no	no	no	no	no	no	no	no	no	no	no	no	no	no	no	no	no	no	no
40	yes	no	no	no	no	yes	no	yes	yes	yes	no	no	yes	no	no	no	no	no	no	no	no
41	yes	no	no	no	no	no	no	yes	yes	yes	no	no	no	no	no	no	no	no	no	no	no
42	no	no	no	no	no	yes	no	yes	no	no	no	no	yes	no	no	no	no	no	no	no	no
43	no	no	no	no	no	yes	no	no	no	no	no	no	no	no	no	no	no	no	no	no	no
44	yes	no	no	no	no	yes	no	no	no	no	no	no	no	no	no	no	no	no	no	no	no
45	no	no	no	no	no	yes	no	no	yes	yes	no	no	no	no	no	no	no	no	no	no	no
46	no	no	no	no	no	no	no	no	no	no	no	no	no	no	no	no	no	no	no	no	no
47	no	no	no	no	no	no	no	no	no	no	no	no	no	no	no	no	no	no	no	no	no
48	no	no	no	no	no	no	no	yes	yes	yes	yes	no	yes	no	yes	no	no	no	no	no	no
49	no	no	yes	no	no	no	no	no	no	no	no	no	no	no	no	no	no	no	no	no	no
50	no	no	no	no	no	no	no	no	no	no	no	no	no	no	no	no	no	no	no	no	yes

51	yes	no	no	yes	no	no	no	no	no	no	yes	yes	no	no	no	no	no	yes	no	yes	no
52	yes	no	yes	no	no	no	yes	no	yes	yes	yes	no	yes	no	no	yes	no	no	no	yes	yes
53	yes	no	no	no	yes	no	no	yes	no	no	no	yes	yes	no	no	no	no	no	no	yes	no
54	yes	no	no	no	no	no	yes	yes	yes	yes	yes	no	yes	no	no	no	no	no	no	no	no
55	yes	no	no	no	no	yes	no	yes	no	no	yes	no	yes	no	no	no	no	no	no	no	no
56	no	no	no	no	no	no	no	no	yes	no	no	no	yes	no	no	no	no	no	no	no	no
57	yes	no	no	no	no	no	no	yes	yes	yes	yes	no	no	no	no	no	no	no	no	no	no
58	no	no	no	no	no	no	no	no	no	no	no	no	no	no	no	no	no	no	no	no	no
59	no	no	no	no	no	no	no	no	no	no	no	no	no	no	no	no	no	no	no	no	no
60	yes	no	no	yes	no	yes	no	no	no	yes	no	no	yes	no	no	no	no	no	no	yes	no
61	no	no	no	no	no	no	no	no	no	no	no	no	no	no	no	no	no	no	no	no	no
62	no	no	no	no	no	no	no	no	no	no	no	no	no	no	no	no	no	no	no	no	no
63	no	no	no	no	no	yes	no	no	no	no	no	no	no	no	no	no	no	no	no	no	no
64	no	no	no	no	no	no	no	no	no	no	no	no	no	no	no	no	no	no	no	no	no
65	yes	no	no	no	no	no	no	yes	no	no	yes	no	yes	no	no	no	no	no	no	no	yes
66	no	no	no	no	no	no	no	no	no	no	no	no	no	no	no	no	no	no	no	no	no
67	no	no	no	no	no	no	yes	yes	no	yes	no	no	yes	no	no	no	no	yes	no	no	no
68	no	no	no	no	no	no	no	yes	no	yes	no	no	yes	yes	yes	no	no	no	no	no	no
69	no	no	no	no	no	no	no	yes	yes	no	no	no	yes	no	yes	no	no	no	no	no	no
70	no	no	no	no	no	no	no	yes	yes	yes	no	no	yes	no	no	no	yes	no	no	no	no
71	yes	no	no	no	yes	no	no	yes	yes	yes	no	yes	yes	no	no	yes	no	no	no	no	no

72	no	no	yes	no	yes	no	yes	yes	no	yes	yes	no	yes	no	no	no	no	yes	no	no	no
73	yes	no	no	yes	yes	no	yes	yes	yes	yes	yes	no	yes	no	yes	no	no	yes	no	no	no
74	no	no	no	yes	no	yes	yes	yes	no	yes	yes	no	yes	no	no	no	no	no	no	no	no
75	yes	yes	no	yes	no	no	yes	no	yes	no	yes	no	yes	no	no	no	no	no	no	no	no
76	no	no	yes	yes	yes	yes	yes	no	no	yes	yes	no	yes	no	yes	no	no	yes	yes	no	yes
77	yes	no	no	no	no	no	yes	yes	no	yes	no	no	yes	no	no	no	no	no	no	yes	no
78	no	no	no	no	no	no	yes	no	yes	no	yes	no	yes	no	no	yes	yes	yes	no	no	no
79	no	no	no	no	no	no	no	no	no	yes	no	no	no	no	no	no	no	yes	no	no	yes
80	yes	no	no	no	no	no	yes	yes	no	yes	yes	no	yes	no	yes	no	no	yes	no	no	no
81	yes	no	no	yes	no	yes	yes	yes	yes	yes	yes	yes	yes	no	no	no	no	yes	no	no	yes
82	no	no	no	no	no	no	no	no	no	no	no	no	no	no	no	no	no	no	no	no	no
83	no	no	yes	yes	no	yes	no	yes	no	yes	yes	no	yes	no	no	no	no	yes	yes	no	yes
84	yes	no	no	no	no	no	no	yes	yes	no	yes	no	yes	no	no	no	no	no	no	no	no
85	yes	no	yes	no	yes	yes	yes	yes	yes	yes	yes	yes	yes	no	no	no	no	yes	no	no	no
86	no	no	no	no	no	no	yes	yes	no	yes	yes	no	yes	no	no	no	no	no	no	no	no
87	yes	no	no	no	no	no	yes	no	yes	no	yes	no	yes	no	no	no	no	no	no	no	no
88	yes	no	no	no	yes	no	no	yes	no	yes	yes	yes	no	no	yes	no	no	yes	no	no	no
89	yes	no	no	no	yes	no	yes	no	yes	yes	yes	no	yes	no	no	no	no	yes	no	no	no
90	yes	no	no	no	no	no	yes	yes	yes	yes	no	no	yes	no	no	no	no	no	no	no	no
91	yes	no	no	no	no	no	no	no	yes	yes	no	no	yes	no	no	no	no	no	no	no	no
92	yes	no	no	no	no	no	no	no	no	yes	yes	yes	yes	no	no	no	no	no	yes	no	yes

	93	yes	no	no	no	yes	yes	yes	yes	yes	no	yes	no	yes	no	no	yes	no	yes	yes	no	no
	94	no	no	no	no	no	no	no	no	no	no	no	no	no	no	no	no	no	no	no	no	yes
	95	no	no	no	yes	no	no	yes	no	no	no	no	no	yes	no	no	no	no	no	no	no	no
	96	no	no	no	no	yes	no	yes	yes	yes	yes	yes	no	yes	no	yes	no	no	yes	no	no	no
	97	yes	no	no	no	no	no	yes	yes	yes	no	no	no	yes	no	no	no	no	yes	no	no	no
	98	yes	no	no	no	no	no	no	no	no	no	no	no	no	no	no	no	no	no	no	no	no
	99	yes	no	no	no	yes	no	yes	yes	no	yes	yes	no	yes	no	no	no	no	yes	no	no	no
	100	yes	no	no	no	no	no	yes	yes	no	yes	yes	yes	yes	no	no	no	no	yes	no	no	no
	101	no	no	no	no	no	no	no	yes	no	no	no	no	yes	no	no	no	no	yes	no	no	no
	102	no	no	no	no	yes	no	yes	yes	no	no	yes	no	yes	no	no	no	no	no	no	no	no
	103	no	no	no	no	yes	no	yes	yes	no	yes	no	no	yes	no	no	no	no	yes	no	no	yes
	104	yes	no	no	no	no	yes	no	yes	no	yes	no	yes	yes	no	no	no	no	no	yes	yes	no
	105	yes	no	no	yes	no	no	no	yes	no	yes	no	no	yes	no	no	no	yes	yes	yes	no	yes
	106	no	no	no	yes	no	no	no	no	yes	yes	yes	no	yes	no	no	no	no	yes	no	no	yes
	Total N	106	106	106	106	106	106	106	106	106	106	106	106	106	106	106	106	106	106	106	106	106
4	1	yes	no	no	no	no	yes	no	yes	no	no	no	no	no	no	no	no	no	no	no	no	no
	2	no	no	no	no	no	no	no	no	yes	no	no	no	no	no	no	no	no	no	no	no	no
	3	no	no	no	no	no	no	no	no	no	no	no	no	no	no	no	no	yes	no	no	no	no
	4	no	no	no	no	no	no	no	yes	yes	no	no	no	no	no	no	no	yes	no	no	no	no

5	yes	no	no	no	no	yes	no	yes	yes	no	no	no	yes	no	no	no	no	no	no	no	no
6	yes	no	no	no	no	no	no	yes	no	yes	no	no	yes	no	no	no	no	no	no	no	no
7	yes	no	no	no	no	no	no	yes	yes	no	no	no	yes	no	no	no	no	no	no	no	no
8	no	no	no	no	no	no	no	yes	yes	no	no	no	yes	no	no	no	no	no	no	no	no
9	yes	no	no	no	no	yes	no	no	yes	no	no	no	no	no	no	no	no	no	no	no	no
10	no	no	no	no	no	no	no	yes	yes	no	no	no	no	no	no	no	no	no	no	no	no
11	yes	no	no	no	no	no	no	no	no	no	no	no	no	no	no	no	no	no	no	no	no
12	no	no	no	no	no	yes	no	no	yes	no	no	no	yes	no	no	no	no	no	no	no	no
13	no	no	no	no	no	yes	no	no	yes	no	no	no	yes	no	no	no	no	no	no	no	no
14	yes	no	no	no	no	no	no	no	yes	no	no	no	yes	no	no	no	no	no	no	no	no
15	yes	no	no	no	no	no	no	no	yes	no	no	no	no	no	no	no	no	no	no	no	no
16	yes	no	no	no	no	no	no	no	no	no	no	no	no	no	no	no	no	no	no	no	no
17	yes	no	no	no	no	no	no	no	no	no	no	no	no	no	no	no	no	no	no	no	no
18	yes	no	no	no	no	yes	no	yes	yes	no	no	no	yes	no	no	no	no	no	no	no	no
19	yes	no	no	no	no	yes	no	yes	yes	no	no	no	yes	no	no	no	no	no	no	no	no
20	yes	no	no	no	no	yes	no	yes	yes	no	no	no	yes	no	no	no	no	no	no	no	no
21	yes	no	no	no	no	no	no	yes	yes	yes	no	no	yes	no	no	no	no	no	no	no	no
22	yes	no	no	no	no	yes	no	yes	yes	yes	no	no	yes	no	no	no	yes	no	no	no	no
23	yes	no	no	no	no	yes	no	yes	yes	yes	no	no	yes	no	no	no	yes	no	no	no	no
24	yes	no	no	no	no	no	no	no	no	no	no	no	no	no	no	no	no	no	no	no	no
25	yes	no	no	no	no	no	no	no	no	no	no	no	no	no	no	no	no	no	no	no	no

26	no	no	no	no	no	yes	no	yes	yes	yes	no	no	yes	no	no	no	no	no	no	no	no
27	yes	no	no	no	no	no	no	no	no	no	no	no	no	no	no	no	no	no	no	no	no
28	yes	no	no	no	no	yes	no	no	yes	no	no	no	yes	no	no	no	no	no	no	no	no
29	yes	no	no	no	no	yes	no	no	yes	no	no	no	yes	no	no	no	no	no	no	no	no
30	yes	no	no	no	no	no	no	no	no	no	no	no	no	no	no	no	no	no	no	no	no
31	yes	no	no	no	no	no	no	no	no	no	no	no	no	no	no	no	no	no	no	no	no
32	yes	no	no	no	no	yes	no	yes	yes	yes	no	no	yes	no	no	no	no	no	no	no	no
33	yes	no	no	no	no	yes	no	yes	yes	no	no	no	yes	no	no	no	no	no	no	no	no
34	no	no	no	no	no	yes	no	no	yes	no	no	no	no	no	no	no	no	no	no	no	no
35	no	no	no	no	no	no	no	no	no	no	no	no	no	no	no	no	no	no	no	no	no
36	yes	no	yes	no	yes	no	yes	yes	yes	no	no	no	yes	no	yes	yes	no	yes	no	no	no
37	no	no	no	no	no	no	no	no	no	no	no	no	no	no	no	no	no	no	no	no	no
38	yes	no	no	no	no	no	no	no	yes	yes	no	no	yes	no	yes	yes	no	no	no	no	no
39	yes	no	no	no	no	no	yes	yes	no	yes	yes	no	no	no	no	no	no	no	no	no	no
40	no	no	no	no	no	yes	no	no	no	no	no	no	no	no	no	no	no	no	no	no	no
41	yes	no	yes	no	no	no	yes	yes	yes	no	yes	no	yes	no	no	no	no	no	no	no	no
42	yes	no	no	no	yes	no	yes	yes	no	no	yes	no	yes	no	no	yes	no	no	no	no	no
43	yes	no	yes	no	no	no	no	yes	yes	no	yes	no	yes	no	no	no	no	no	no	no	no
44	no	no	no	no	no	no	no	no	no	no	no	no	no	no	no	no	no	no	no	no	no
45	no	no	no	no	no	no	no	no	no	no	no	no	no	no	no	no	no	no	no	no	no
46	no	no	no	no	no	no	no	no	no	no	no	no	no	no	no	no	no	no	no	no	no

47	yes	no	no	no	yes	yes	no	no	no	yes	yes	no	no	no	no	no	no	no	no	no	yes
48	yes	no	no	no	no	yes	yes	yes	yes	yes	yes	yes	yes	no	no	no	no	no	no	yes	no
49	no	no	yes	no	no	no	no	yes	yes	no	yes	no	yes	no	no	no	no	no	no	no	no
50	no	no	no	no	no	no	no	no	no	no	no	no	no	no	no	no	no	no	no	no	no
51	yes	no	yes	yes	no	yes	yes	no	yes	yes	no	no	yes	no	no	no	no	yes	no	yes	no
52	yes	no	no	no	no	no	yes	no	no	yes	yes	yes	yes	no	no	no	no	no	no	no	yes
53	yes	no	no	yes	no	yes	no	yes	yes	yes	yes	no	yes	no	no	no	yes	yes	no	no	yes
54	yes	no	no	no	no	no	yes	yes	yes	no	yes	no	yes	no	no	no	no	no	no	no	no
55	yes	no	yes	no	no	no	yes	yes	yes	yes	no	no	yes	no	yes	no	no	yes	no	no	no
56	no	no	no	no	no	no	no	no	no	no	no	no	no	no	no	no	no	no	no	no	no
57	yes	no	no	yes	no	no	yes	no	no	no	yes	no	yes	no	no	no	no	no	no	no	no
58	yes	yes	yes	yes	yes	no	yes	yes	yes	yes	no	no	yes	no	yes	no	no	no	no	no	no
59	yes	no	no	yes	no	yes	no	yes	yes	no	yes	no	yes	yes	no	no	no	no	no	no	no
60	yes	no	no	no	yes	yes	yes	yes	no	yes	yes	no	yes	yes	no	no	no	yes	no	no	no
61	no	no	yes	no	no	yes	yes	no	no	no	yes	no	yes	no	no	no	no	no	no	no	yes
62	no	no	no	no	no	no	no	yes	no	yes	no	no	no	no	no	no	no	no	no	no	no
63	no	no	no	no	no	no	no	no	no	no	no	no	yes	no	no	no	no	no	no	no	yes
64	no	no	no	no	no	no	no	yes	no	yes	yes	yes	no	no	no	no	no	no	no	no	no
65	yes	no	yes	yes	no	yes	yes	yes	yes	yes	no	yes	yes	no	no	no	no	yes	no	no	no
66	no	no	no	yes	no	no	yes	no	no	yes	no	no	yes	no	no	no	no	no	yes	no	yes
67	no	no	no	no	no	yes	no	no	no	yes	no	no	yes	no	no	no	no	no	no	no	no

	68		yes	no	no	no	no	no	yes	yes	no	no	no	no	yes	no	no	no	no	no	yes	no	no
	69		no	no	no	no	no	no	no	no	no	no	no	no	yes	no	no	no	no	no	no	no	yes
	70		yes	yes	yes	yes	no	no	no	yes	no	yes	no	no	yes	no	no	no	no	yes	no	no	no
	71		no	no	no	no	no	no	no	no	no	no	no	no	no	no	no	no	no	no	no	no	yes
	72		no	no	no	no	no	no	yes	yes	no	yes	yes	no	yes	no	no	no	no	no	no	no	no
	73		yes	no	no	no	yes	no	yes	yes	no	no	no	yes	yes	no	no	no	no	no	no	no	yes
	74		no	no	no	no	no	no	no	no	no	no	no	no	no	no	no	no	no	no	no	no	no
	75		no	no	no	no	no	yes	yes	yes	yes	yes	no	no	yes	no	no	no	no	yes	no	no	yes
	76		no	no	no	no	no	no	yes	no	yes	no	yes	no	yes	no	no	yes	yes	yes	no	no	no
	77		no	no	no	no	no	no	no	yes	no	yes	no	no	no	no	no	no	no	no	no	no	no
	78		yes	no	no	no	yes	no	no	yes	no	yes	yes	no	yes	no	no	no	no	no	no	no	no
	79		yes	yes	no	no	no	no	no	no	no	yes	no	no	yes	no	no	no	no	no	no	no	no
	80		yes	no	no	no	no	no	no	no	no	yes	no	yes	yes	no	no	yes	no	no	no	no	yes
	81		yes	no	no	no	no	yes	yes	yes	no	yes	yes	no	yes	no	no	no	no	yes	no	no	yes
	Total	N	81	81	81	81	81	81	81	81	81	81	81	81	81	81	81	81	81	81	81	81	81
Total	N		285	285	285	285	285	285	285	285	285	285	285	285	285	285	285	285	285	285	285	285	285

a. Limited to first 300 cases.

SUPPLEMENTARY MATERIAL 4

Regression, Interaction and Assumptions analysis

Multivariate and interaction analysis

Table S1: Case Processing Summary

Case Processing Summary

Unweighted Cases[a]		N	Percent
Selected Cases	Included in Analysis	285	100.0
	Missing Cases	0	.0
	Total	285	100.0
Unselected Cases		0	.0
Total		285	100.0

a. If weight is in effect, see classification table for the total number of cases.

Table S2: Dependent Variable Encoding

Dependent Variable Encoding

Original Value	Internal Value
non-adherent	0
adherent	1

Table S3: Categorical Variables Codings

Categorical Variables Codings

		Frequency	Parameter coding (1)
Sex	male	217	.000
	female	68	1.000

Table S4: Classification Table[a,b]

Block 0: Beginning Block

Classification Table[a,b]

	Observed		Predicted MARS non-adherent	adherent	Percentage Correct
Step 0	MARS	non-adherent	0	81	.0
		adherent	0	204	100.0
	Overall Percentage				71.6

a. Constant is included in the model.

b. The cut value is .500

Table S5: Variables in the Equation

Variables in the Equation

		B	S.E.	Wald	df	Sig.	Exp(B)
Step 0	Constant	.924	.131	49.466	1	<.001	2.519

Table S6: Variables not in the Equation

Variables not in the Equation

			Score	df	Sig.
Step 0	Variables	WHO5_c	.056	1	.813
		GOLD_c	.016	1	.898
		WHO5xGOLD	.234	1	.629
		Age	.157	1	.692
		Sex(1)	.010	1	.920
		FEV1FVC	.515	1	.473
		mMRC	.671	1	.413
	Overall Statistics		4.555	7	.714

Table S7: Omnibus Tests of Model Coefficients

Block 1: Method = Enter

Omnibus Tests of Model Coefficients

		Chi-square	df	Sig.
Step 1	Step	4.596	7	.709
	Block	4.596	7	.709
	Model	4.596	7	.709

Table S8: Model Summary

Model Summary

Step	-2 Log likelihood	Cox & Snell R Square	Nagelkerke R Square
1	335.629[a]	.016	.023

a. Estimation terminated at iteration number 4 because parameter estimates changed by less than .001.

Table S9: Hosmer and Lemeshow Test

Hosmer and Lemeshow Test

Step	Chi-square	df	Sig.
1	7.741	8	.459

Table S10: Contingency Table for Hosmer and Lemeshow Test

Contingency Table for Hosmer and Lemeshow Test

		MARS = non-adherent		MARS = adherent		
		Observed	Expected	Observed	Expected	Total
Step 1	1	15	11.270	14	17.730	29
	2	6	9.964	23	19.036	29
	3	11	9.340	18	19.660	29
	4	8	8.825	21	20.175	29
	5	8	8.365	21	20.635	29
	6	7	8.183	23	21.817	30
	7	5	7.503	24	21.497	29
	8	7	6.931	22	22.069	29
	9	8	6.289	21	22.711	29
	10	6	4.329	17	18.671	23

Table S11: Classification Table[a]

Classification Table[a]

			Predicted		
			MARS		
	Observed		non-adherent	adherent	Percentage Correct
Step 1	MARS	non-adherent	0	81	.0
		adherent	0	204	100.0
	Overall Percentage				71.6

a. The cut value is .500

Table S12: Variables in the Equation

Variables in the Equation

		B	S.E.	Wald	df	Sig.	Exp(B)	95% C.I.for EXP(B) Lower	Upper
Step 1[a]	WHO5_c	-.005	.008	.482	1	.488	.995	.980	1.010
	GOLD_c	.447	.262	2.897	1	.089	1.563	.935	2.614
	WHO5xGOLD	.004	.007	.284	1	.594	1.004	.989	1.019
	Age	.007	.014	.265	1	.606	1.007	.979	1.036
	Sex(1)	.057	.315	.032	1	.857	1.058	.571	1.960
	FEV1FVC	.007	.008	.667	1	.414	1.007	.990	1.024
	mMRC	-.450	.240	3.512	1	.061	.638	.399	1.021
	Constant	1.180	1.185	.991	1	.320	3.254		

a. Variable(s) entered on step 1: WHO5_c, GOLD_c, WHO5xGOLD, Age, Sex, FEV1FVC, mMRC.

Model assumptions

Table S13: Model Summary[b]

Model Summary[b]

Model	R	R Square	Adjusted R Square	Std. Error of the Estimate
1	.241[a]	.058	.038	.936

a. Predictors: (Constant), Sex, MARS, Age, FEV1FVC, WHO_5, CAT
b. Dependent Variable: GOLD

Table S14: ANOVA

ANOVA[a]

Model		Sum of Squares	df	Mean Square	F	Sig.
1	Regression	15.064	6	2.511	2.866	.010[b]
	Residual	243.512	278	.876		
	Total	258.575	284			

a. Dependent Variable: GOLD
b. Predictors: (Constant), Sex, MARS, Age, FEV1FVC, WHO_5, CAT

Table S15: Collinearity Diagnostics[a]

Collinearity Diagnostics[a]

Model	Dimension	Eigenvalue	Condition Index	Variance Proportions (Constant)	CAT	MARS	WHO_5	FEV1FVC	Age	Sex
1	1	6.405	1.000	.00	.00	.01	.00	.00	.00	.00
	2	.265	4.914	.00	.01	.88	.01	.00	.00	.01
	3	.146	6.634	.00	.13	.01	.35	.00	.00	.00
	4	.102	7.939	.00	.01	.00	.01	.10	.00	.79
	5	.051	11.248	.00	.19	.01	.16	.78	.01	.11
	6	.026	15.831	.02	.36	.05	.35	.08	.48	.01
	7	.007	30.579	.98	.30	.05	.13	.03	.51	.08

a. Dependent Variable: GOLD

Table S16: Residuals Statistics[a]

Residuals Statistics[a]

	Minimum	Maximum	Mean	Std. Deviation	N
Predicted Value	2.07	3.56	2.84	.230	285
Residual	-2.201	1.589	.000	.926	285
Std. Predicted Value	-3.337	3.116	.000	1.000	285
Std. Residual	-2.352	1.698	.000	.989	285

a. Dependent Variable: GOLD

Figure S1: Scatterplot – Dependent Variable: GOLD

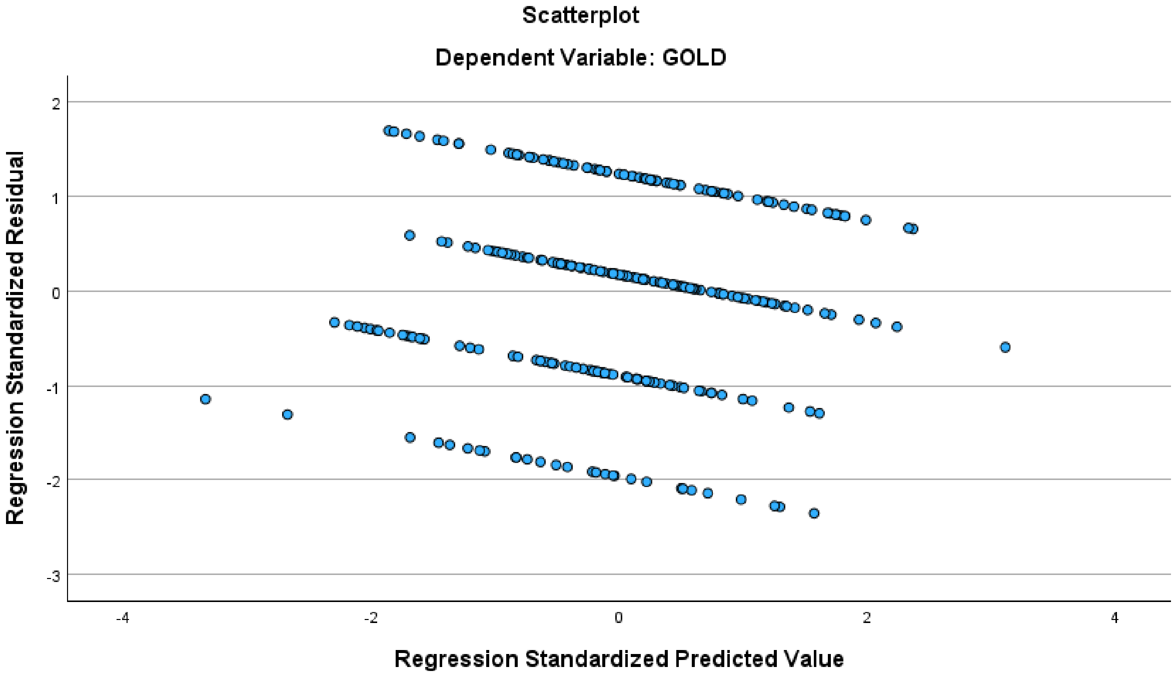

Table S17: Variables in the Equation

Variables in the Equation

		B	S.E.	Wald	df	Sig.	Exp(B)	95% C.I.for EXP(B)	
								Lower	Upper
Step 1[a]	WHO5_c	-.005	.008	.482	1	.488	.995	.980	1.010
	GOLD_c	.447	.262	2.897	1	.089	1.563	.935	2.614
	WHO5xGOLD	.004	.007	.284	1	.594	1.004	.989	1.019
	Age	.007	.014	.265	1	.606	1.007	.979	1.036
	Sex(1)	.057	.315	.032	1	.857	1.058	.571	1.960
	FEV1FVC	.007	.008	.667	1	.414	1.007	.990	1.024
	mMRC	-.450	.240	3.512	1	.061	.638	.399	1.021
	Constant	1.180	1.185	.991	1	.320	3.254		

a. Variable(s) entered on step 1: WHO5_c, GOLD_c, WHO5xGOLD, Age, Sex, FEV1FVC, mMRC.

Table S18: Coefficients[a]

Coefficients[a]

Model	Unstandardized Coefficients	Standardized Coefficients	t	Sig.	95.0% Confidence Interval for B	Collinearity Statistics

		B	Std. Error	Beta			Lower Bound	Upper Bound	Tolerance	VIF
1	(Constant)	4.183	.590		7.085	<.001	3.021	5.345		
	CAT	.012	.010	.086	1.226	.221	-.007	.032	.690	1.450
	MARS	.070	.127	.033	.551	.582	-.179	.319	.942	1.061
	WHO_5	.000	.004	-.006	-.088	.930	-.007	.007	.702	1.425
	FEV1FVC	-.007	.003	-.112	-1.899	.059	-.014	.000	.967	1.034
	Age	-.017	.006	-.173	-2.913	.004	-.029	-.006	.963	1.038
	Sex	-.095	.131	-.042	-.722	.471	-.353	.164	.983	1.017

a. Dependent Variable: GOLD

Table S19. Collinearity diagnostics for the multivariable linear regression model with GOLD stage as the dependent variable.

Collinearity Diagnostics[a]

Model	Dimension	Eigenvalue	Condition Index	Variance Proportions						
				(Constant)	CAT	MARS	WHO_5	FEV1FVC	Age	Sex
1	1	6.405	1.000	.00	.00	.01	.00	.00	.00	.00
	2	.265	4.914	.00	.01	.88	.01	.00	.00	.01
	3	.146	6.634	.00	.13	.01	.35	.00	.00	.00
	4	.102	7.939	.00	.01	.00	.01	.10	.00	.79
	5	.051	11.248	.00	.19	.01	.16	.78	.01	.11
	6	.026	15.831	.02	.36	.05	.35	.08	.48	.01
	7	.007	30.579	.98	.30	.05	.13	.03	.51	.08

a. Dependent Variable: GOLD
